# Synthesis, antimicrobial and cytotoxicity evaluation of new cholesterol congeners

**DOI:** 10.3762/bjoc.11.208

**Published:** 2015-10-16

**Authors:** Mohamed Ramadan El Sayed Aly, Hosam Ali Saad, Shams Hashim Abdel-Hafez

**Affiliations:** 1 Chemistry Department, Faculty of Science, Taif University, 21974-Hawyah-Taif, Kingdom of Saudi Arabia; 2 Chemistry Department, Faculty of Applied Science, Port Said University, 42522-Port Said, Egypt; 3 Chemistry Department, Faculty of Science, Zagazig University, Zagazig, 44511, Egypt; 4 Chemistry Department, Faculty of Science, Assuit University, 71516-Assuit, Egypt

**Keywords:** antimicrobial, chalcone, cholesterol, cytotoxicity, glycoside, triazole

## Abstract

3β-Azidocholest-5-ene (**3**) and (3β)-3-(prop-2-yn-1-yloxy)cholest-5-ene (**10**) were prepared as substrates to synthesize a variety of three-motif pharmacophoric conjugates through CuAAC. Basically, these conjugates included cholesterol and 1,2,3-triazole moieties, while the third, the pharmacophore, was either a chalcone, a lipophilic residue or a carbohydrate tag. These compounds were successfully prepared in good yields and characterized by NMR, MS and IR spectroscopic techniques. Chalcone conjugate **6c** showed the best antimicrobial activity, while the lactoside conjugate **27** showed the best cytotoxic effect in vitro.

## Introduction

Cholest-5-en-3β-ol (cholesterol, **1**) is an amphiphilic-like steroidal constituent of eukaryotic cell membranes. It acts as fluidity buffer and it is essential for membrane integrity and permeability. Besides, it is a substrate for the biosynthesis of steroidal hormones, bile acids and vitamin D. Pathological accumulation of oxygenated cholesterol (oxysterol) metabolites contributes to the prognosis of major chronic diseases. Cholesterol is completely absent in prokaryotic organisms [1–3].

**Figure 1:**
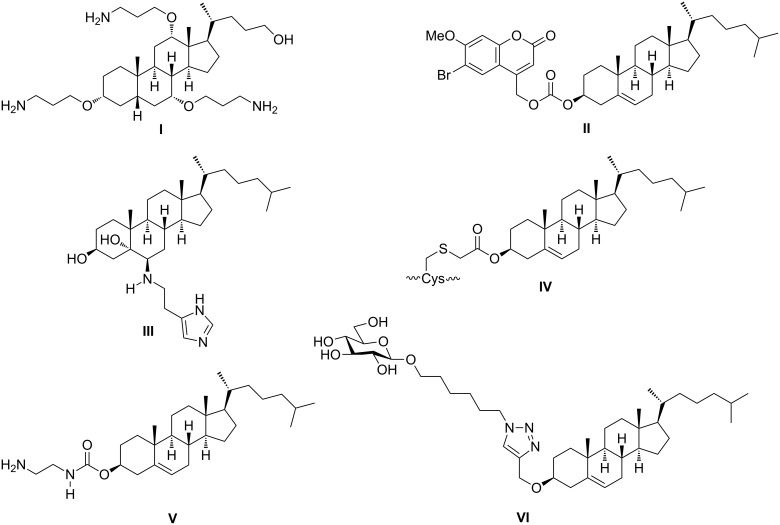
Structure of ceragenin (CSA-8) and selected cholesterol conjugates.

Cholesterol gives eukaryotic membranes sufficient mechanical stiffness against cationic selective antimicrobials (CSAs) such as antimicrobial peptides (AMPs) [4] and ceragenins **I** (Figure 1) [5]. These CSAs selectively bind to the over expressed negatively charged peripheral phospholipids on the internal bacterial cell membranes. Following membrane association, deformation occurs causing bilayer destabilization and cell lysis [6]. According to this mechanism, synthetic polycarbonates arising from organocatalytic ring-opening polymerization of cholesterol monomers were reported to create self-assemblies possessing high interior charge density and wide spectrum antimicrobial activity [6]. Interestingly, the causative vector of human gastritis and peptic ulcer *Helicobacter pylori* is known to elevate serum cholesterol levels in infected patients. This bacterial strain elevates the serum cholesterol levels and involves a specific enzyme known as cholesterol-α-glucosyltransferase to glycosylate cholesterol via α-glycosidic linkage and incorporates it into its cytoplasmic membrane. In this way it boosts resistance to host immune defense and antibiotics as well [5,7].

In another case, eukaryotic cell membranes are supported by a membrane-associated cholesterol efflux regulatory protein (CERP). This protein, also known as ABCA1, is a major regulator of cellular cholesterol [8]. Synthetic BODIPY–cholesterol conjugates were reported as probes for visualization of intracellular cholesterol pools and for monitoring cholesterol efflux from cells to extracellular receptors [9]. ABCA1 plays an inevitable role in the resistance against tumorgenesis through depletion of cholesterol from cells under cancer threat, where cancer onset requires elevated intracellular cholesterol levels to build new membranes [10]. This emerging propensity of nascent cancer cells for cholesterol uptake is an attractive target to use cholesterol as vehicle to increase the bioavailability of anticancer drugs. Thus, SuberAniloHydroxamic acid–cholesterol conjugates (SAHA–cholesterol) [11], cholesterol-based charged liposomes encaging doxorubicin [12] or curcumin [13] showed higher activity compared with the native drugs. Synthetic coumarin-caged cholesterol derivatives, for instance **II**, were triggered to release bioactive coumarines by photolysis at 350 nm [14]. Dendrogenin A (DDA, **III**) is a natural metabolite in healthy mammals. It arises from conjugation of 5,6α-epoxycholesterol (5,6α-EC) with histamine. In vitro studies showed that DDA induced tumor cell re-differentiation and death. This explains why it is down-regulated during carcinogenesis and opens the door for nucleophilic addition of amines to 5,6α-EC as a new lead for developing potential anticancer drugs [15]. Apart from antimicrobial and antiproliferative activities of cholesterol derivatives, other pharmacologic activities were reported for them. Thus, cholesterol-conjugated C-peptides, for example **IV**, are potent inhibitors of the Ebola virus glycoprotein-mediated cell entry [16], while cholesterol-derived amines exhibited a strong antiviral activity against the influenza A virus (IFV). These amines were able to disrupt the cholesterol-rich lipid envelope and inactivate viral invasion [17]. Cholesterol-based hydrazones exhibited insecticidal activity against the larval stage of *Mythimna separate* (Walker) [18]. Cholesterol–carbamate conjugates, for instance (3β)-cholest-5-en-3-yl (2-aminoethyl)carbamate (**V**), were used to prepare nontoxic unilamellar vesicles as nanocarriers for gene delivery into Neuro2A cells, which are involved in neurodegenerative diseases [19]. Also, the involvement of cholesterol metal ion complexes in Alzheimer’s disease was reviewed [20]. Cholesterol glycosides are known for their immunostimulant activities [21]. Finally, the ability of cholesterol derivatives to self-assembly and gelation as supramolecular gels was reviewed [22]. They are beneficially applicable in materials science, reaction media, sensing and responsive materials, energy supply, biomedicine, and tissue engineering [23].

In light of this emerging propensity of cholesterol-based architectures to assimilate a plenty of pharmacological activities, cholesterol was propargylated, then reacted with azido-modified quinoline and glucopyranosyl derivatives as part of a previous study [24] to discover new antimicrobial and cytotoxic lead structures. Cholesterol conjugate **VI** (Figure 1) arose from this consideration to be more active than ampicillin against the Gram-negative bacterial strain *Escherichia coli* (ATCC 11775) and the Gram-positive bacterial strain *Staphylococcus aureus* (ATTC 12600), while its antifungal activities against the filamentous fungal strain *Aspergillus flavus* (Link) and the yeast forming fungal strain *Candida albicans* (ATCC 7102) were moderate compared with amphotericin B in vitro. In the cytotoxicity study, this derivative was the most cytotoxic one against the prostate cancer PC3 cell line but it was 2.3 fold less active than doxorubicin in vitro. Therefore, this article describes the synthesis of analogues of **VI** with different lipid, glycon and chalcone [25–26] tags to assay and evaluate their in vitro antimicrobial and cytotoxic activities against the above mentioned microbial organisms and the prostate cancer PC3 cell line. It is worth mentioning that the bacterial [27–28] and fungal [29–30] strains in this consideration were elected as they represent the main microbial classes for our in vitro antimicrobial evaluation. On the other hand, prostate cancer was considered because it is highly prevalent malignancy and on the second place in the list of cancer-related deaths due to its high metastatic potential [31].

## Results and Discussion

### Chemistry

**Scheme 1:**
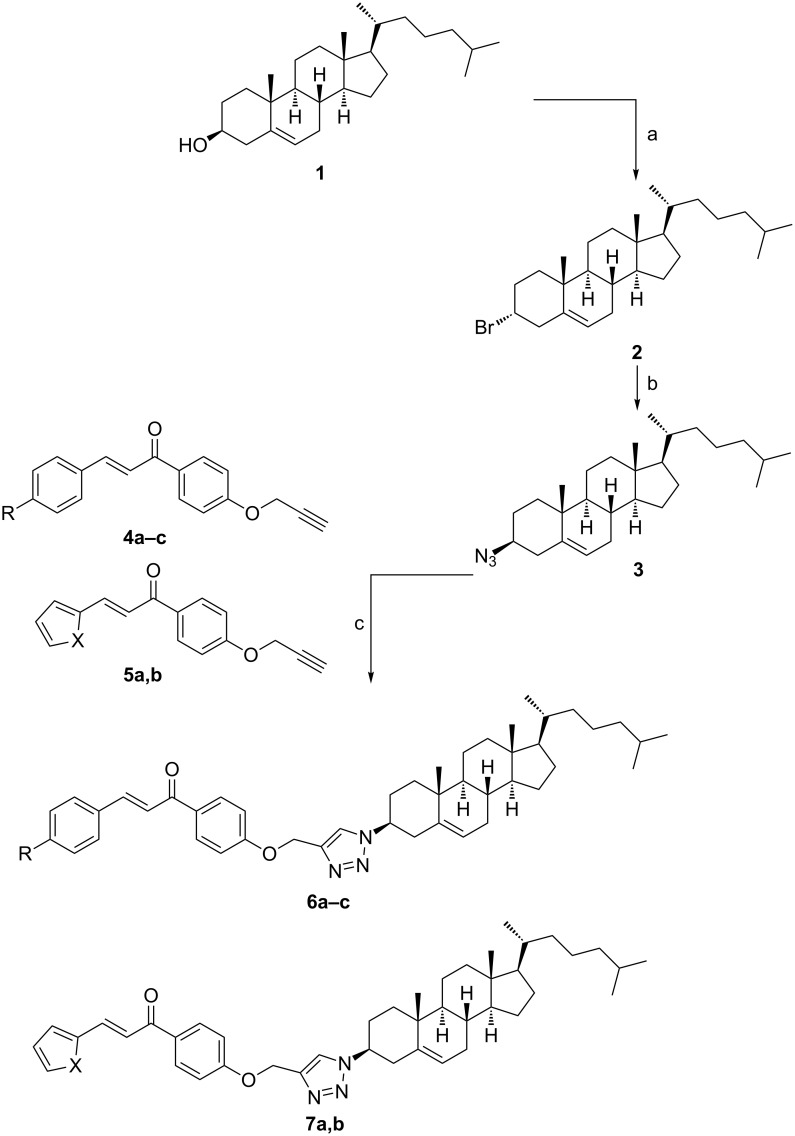
Reagents and conditions: (a) CBr_4_, PPh_3_, DCM (74%); (b) NaN_3_, DMF, 100 °C (63%); (c) CuSO_4_·5H_2_O, L-ascorbic acid (L-AsAc), THF/H_2_O [**6a**, R = H (40%); **6b**, R = OMe (41%); **6c**, R = NMe_2_ (68%); **7a**, X = O (47%); **7b**, X = S (60%)].

Cholest-5-en-3β-ol (**1**) was activated as bromide in very good yield under Appel conditions [32], which means treatment with CBr_4_/PPh_3_ to afford 3α-bromocholest-5-ene **2** due to inversion of the configuration at the C-3 carbon (Scheme 1). The O–H_str_ band of **1** disappeared upon this step. An S_N_2 substitution of the bromine atom of compound **2** with the N_3_ group was ensued by refluxing with NaN_3_ in dry DMF to afford 3β-azidocholest-5-ene (**3**) after inversion of the configuration again at the C-3 carbon. The product could be isolated in good yield and the IR spectrum showed the N_3_ stretching as medium band at 2081 cm^−1^. Other methods for related syntheses were reported in [33–34].

The target cholesterol–chalcone conjugates **6a–c** and **7a,b** were prepared by reacting 3β-azidocholest-5-ene (**3**) with propargylated chalcones **4a–c** and **5a**,**b** [24] under CuAAC conditions [35]. The reactions proceeded fairly in gently refluxing THF/H_2_O mixture containing L-ascorbic acid as reducing agent and a catalytic amount of CuSO_4_·5H_2_O.

The ^13^C NMR spectra of this series showed the C=O signal at δ values within the range of 187–188 ppm, while the ^1^H NMR spectra showed the *trans* configuration of the enone moiety due to the high coupling constant of *J*_α,β_ 15.6 Hz, with the β-proton being more deshielded than the α-proton. The OCH_2_ signal was clearly observed in all derivatives at δ ≈ 5.30 ppm. On the other hand, the olefinic H-6 of cholesterol was observed at δ ≈ 5.40 ppm. The ^1^H NMR spectra of these compounds also showed the CH_3_-25 and CH_3_-26 signals of cholesterol as doublets at δ ≈ 0.86 and 0.87 ppm with a coupling constant of *J* = 3.0 Hz. The CH_3_-21 was observed as doublet nearby the previous signals, while the CH_3_-18 singlet was the most shielded at δ ≈ 0.85 ppm in all spectra. These five ^1^H NMR signals seemed to be a NMR identity fingerprint region of cholesterol. All these spectral data, besides the recorded mass peaks at *m*/*z* values corresponding to the exact molecular weight of each derivative supported these azide–alkyne cycloadditions.

**Scheme 2:**
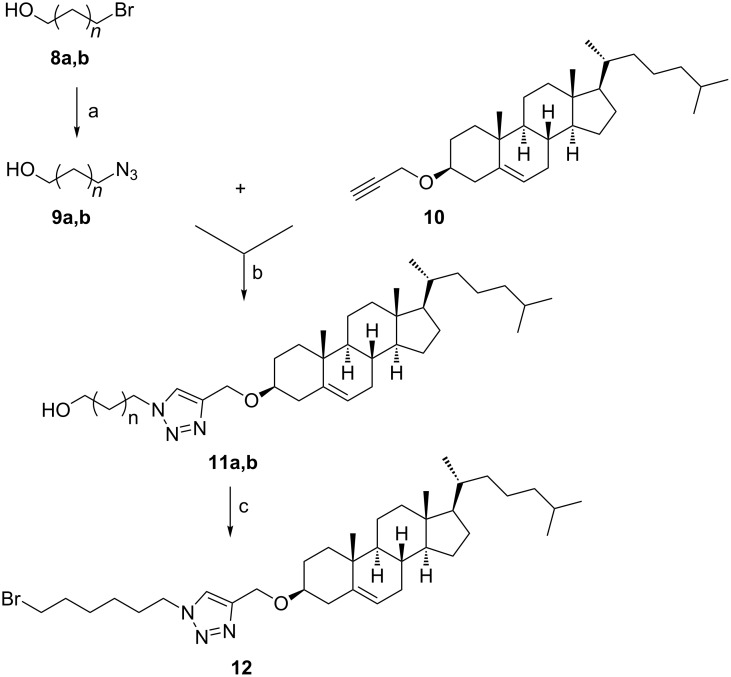
Reagents and conditions: (a) NaN_3_, DMF, 100 °C (**9b**, 47%); (b) CuSO_4_·5H_2_O, L-AsAc, THF/H_2_O [**11a**, *n* = 4 (90%); **11b**, *n* = 9 (67%)]; (c) CBr_4_, PPh_3_, DCM (59%).

**Scheme 3:**
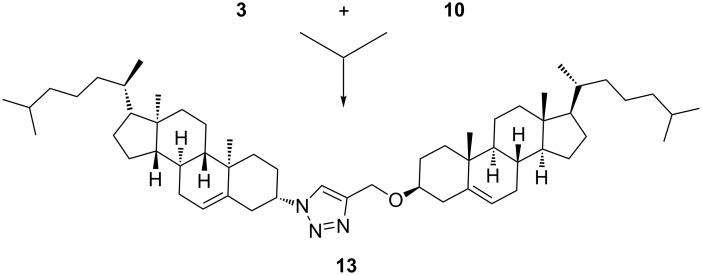
Reagents and conditions: CuSO_4_·5H_2_O, L-AsAc, THF/H_2_O (96%).

The second set of cholesterol conjugates (Scheme 2 and Scheme 3) was prepared by CuAAC of (3β)-3-(prop-2-yn-1-yloxy)cholest-5-ene (**10**) with azidoalcanols **9a**,**b** [24] and 3β-azidocholest-5-ene (**3**). These investigations aimed to address whether the terminal surface recognition glycon tag was necessary to stimulate the biological activity of triazolocholesterol [24] or just an alternative unique OH group, as in conjugates **11a**,**b**, or even without it as in derivatives **12** and **13**, can retain its activity. Particularly, hydroxyalkyl-1,2,3-triazoles were reported as valuable pharmacophores [36].

The products were isolated in good yields and the H-5 signal of triazole (^1^H NMR) could be observed as a singlet at δ ≈ 7.5 ppm. Compound **11a** was further converted into the corresponding bromo derivative **12** in good yield under the same conditions used to prepare compound **2**. This step aimed to have an alkylating probe that might target nucleic acids or proteins in the tested biological systems.

The 1,2,3-triazole-bridged bicholesterol **13** (Scheme 3) was prepared in excellent yield. The H-5 signal of triazole (^1^H NMR) also was observed as singlet at δ = 7.78 ppm confirming the cycloaddition of derivatives **3** and **10**.

**Scheme 4:**
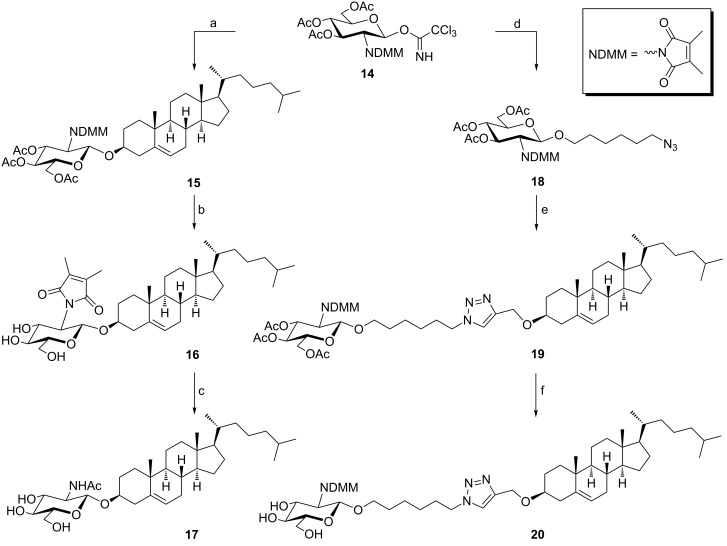
Reagents and conditions: (a) **1**, TMSOTF, CH_3_CN, rt (74%); (b) NaOMe, MeOH (84%); (c) NaOH; HCl (pH 5); Ac_2_O/Pyr; NaOMe/MeOH (37%); (d) **9a**, TMSOTf, DCM (71%); (e) **10**, CuSO_4_·5H_2_O, L-AsAc, THF/H_2_O (67%); (f) NaOMe, MeOH (75%).

D-Glucosamine is an essential constituent of many naturally occurring oligosaccharides such as bacterial and fungal cell walls. Mainly, it is available as *N*-acetylglucosamine in β-glycosidic linkages (β-D-Glc*N*Ac) [37]. Chitinases are special enzymes involved in processing this valuable metabolite. Therefore, triazolocholesterol–glucopyranosylamine conjugates **16**, **17** and **20** (Scheme 4) were prepared to compare the pharmacological effects of the modification of the D-glucopyranose moiety in **VI** (Figure 1) as glucosamine in different forms and with a hexyl spacer. Retaining the dimethylmaleoyl (DMM) group in targets **16** and **20** was based on the finding that NDMM-protected phosphatidylcholine showed better antiproliferative activity than its natural hydrochloride congener [38].

Thus, to prepare these targets glucosyl donor **14** [39] was coupled with cholest-5-en-3β-ol (**1**) as glycosyl acceptor in the presence of catalytic TMSOTf as promoter to afford **15** in 74% yield. The large anomeric coupling constant (*J*_1,2_ = 8.4 Hz) of the pyranoside moiety at δ = 5.30 ppm ensured the β-configuration of this glycoside.

Deacetylation of intermediate **15** under Zémplen conditions, i.e., catalytic NaOMe in MeOH [40], safely afforded the target conjugate **16** in 84% yield without affecting the DMM group. Despite, the two C=O groups could not be seen with certain at δ ≈ 174.00 ppm (^13^C NMR), the two maleimide CH_3_ groups were observed at δ = 8.80 ppm as a proof of structure.

Substitution of the DMM group with an acetyl group was performed under standard conditions, i.e., treatment with NaOH for ring opening [39], HCl at pH 5 for amide cleavage, peracetylation and then *O*-deacetylation. Under these conditions, compound **17** was prepared in 37% yield. The C=O signal (^13^C NMR) was observed at δ = 183.10 ppm with concurrent disappearance of the maleimide CH_3_ signal. A peak was observed at *m*/*z* value corresponding to the exact molecular mass.

Conjugate **20** was prepared under similar conditions as employed for the synthesis of compound **17**. Thus, coupling of azidohexanol **9a** with trichloroacetimidate **14** afforded the intermediate β-glycoside **18** (*J*_1,2_ = 8.4 Hz at δ = 5.18 ppm) in 71% yield. CuAAC of derivative **18** with compound **10** afforded compound **19** in 67% yield. The H-5 proton of the triazole moeity was observed at δ = 7.49 ppm which confirms a successful cycloaddition step. Deacetylation of **19** afforded target spacer linked conjugate **20** in 75% yield. Unlike compound **16**, the two C=O ^13^C NMR signals of conjugate **20** were clearly observed at δ = 174.28 ppm.

**Scheme 5:**
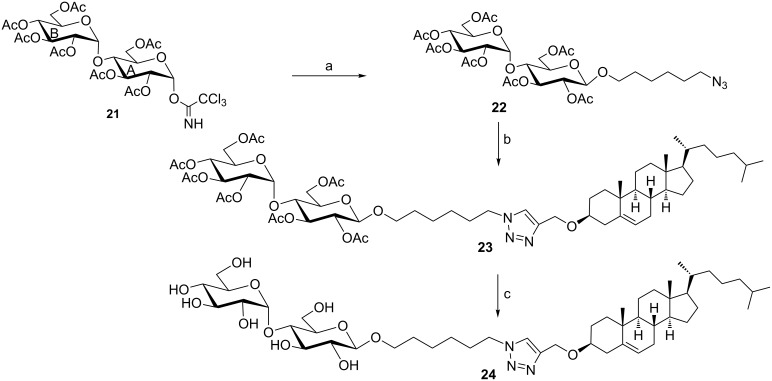
Reagents and conditions: (a) **9a**, TMSOTF, DCM, rt (19%); (b) **10**, CuSO_4_·5H_2_O, L-AsAc, THF/H_2_O (62%); (c) NaOMe/MeOH (78%). A: Ring A of the maltose moiety, B: Ring B of the maltose moiety.

Then, attention was given to prepare conjugate **24** (Scheme 5). This is to investigate the pharmacological effects of the maltose tag compared with glucose as previously investigated in the case of **VI** [24]. Thus, coupling of glycosyl donor **21** [41] with acceptor **9a** afforded maltoside **22** in low yield (Scheme 5). CuAAC of substrate **22** with **10** yielded derivative **23** in 62% yield. The ^1^H NMR showed that the B ring of the maltose moiety was α-configurated at the glycosidic center (H-1_B_ at δ = 5.40 ppm, *J*_1,2_ = 4.2 Hz (see Scheme 5 compound **21** for the assignment of rings A and B of the maltose moiety) and the A ring β-configurated (H-1_A_ at δ = 4.49 ppm, *J*_1,2_ = 7.8 Hz). The traizole H-5 was observed as singlet at δ = 7.52 ppm, while the cholesterol CH_3_ groups fingerprint signals were observed in the upfield region of the spectrum.

**Scheme 6:**
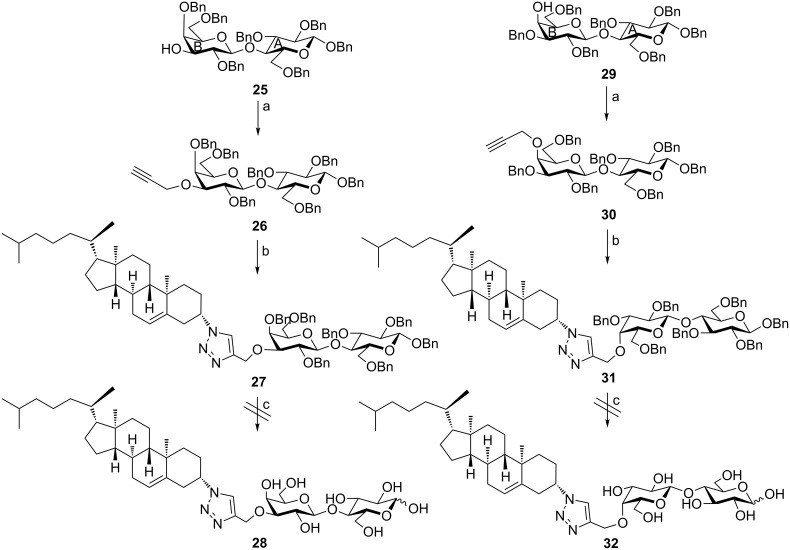
Reagents & conditions: (a) Propargyl bromide, NaH, Et_2_O/DMF (quant. for both **26** and **30**); (b) **3**, CuSO_4_·5H_2_O, L-AsAc, THF/H_2_O (89% for **27** and 74% for **31**); (c) H_2_, Pd/C 10%, MeOH. A: Ring A of the lactose moiety, B: Ring B of the lactose moiety.

Deacetylation of compound **23** smoothly afforded the target conjugate **24** in 78% yield. Finally, compound **28** (Scheme 6) was attempted to be prepared to investigate the cytotoxicity of a lactose scaffold with a cholesterol moiety at the C-3 carbon of the B ring of the lactose. This is because chemically modified 3β-lactosides were emerged as potential galectin-3 inhibitors. Galectin-3 is a member of the protein family known as galectins and this subfamily is believed to be involved in tumorigenesis [42–43]. To this endeavor, 3β-*O*-propargylated lactose derivative **26** was reacted with compound **3** to afford intermediate triazole bridged conjugate **27** in 89% yield. This intermediate was attempted to be debenzylated by treatment with H_2_ and Pd/C 10% but the benzyl groups could not be removed under these conditions.

For comparison reasons, derivative **31**, that has the cholesterol moiety at the C-4 carbon of the B ring of the lactose moiety, was prepared by CuAAC of substrate **30** with **3** to afford intermediate **31** in 74% yield. This intermediate resisted reductive debenzylation under the same conditions and compound **32** also could not be obtained [44–45].

**Scheme 7:**
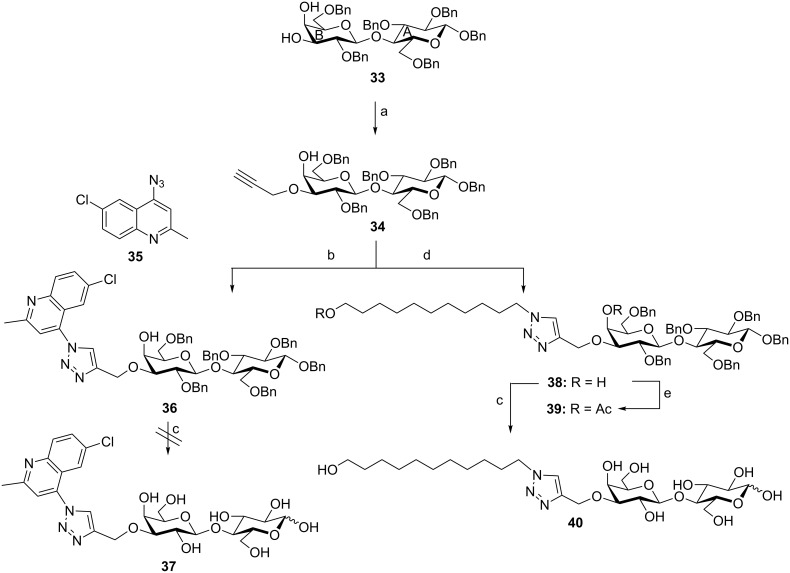
Reagents and conditions: (a) Bu_2_SnO, MeOH; propargyl bromide, TBAI, Tol (92%); (b) CuSO_4_·5H_2_O, L-AsAc, THF/H_2_O (76%); (c) H_2_, Pd/C 10%, MeOH (0% for **37** and 62% for **40**); (d) **9b**, CuSO_4_·5H_2_O, L-AsAc, THF/H_2_O (71%); (e) Ac_2_O/Pyr (90%). A: Ring A of the lactose moiety, B: Ring B of the lactose moiety.

To investigate, whether the triazole or the cholesterol entities are not compatible with these debenzylation conditions, probes **36** and **38** (Scheme 7) were prepared by CuAAC of derivative **34** with compounds **35** and **9b**, respectively [24].

Both conjugates were prepared in very good yields. Probe **38** was acetylated to confirm the regioselectivity at the C-4 position of the lactose B ring [46].

The ^1^H NMR of compound **39**, thus, showed the H-4 proton of the lactose B ring discriminated at δ = 5.46 ppm with a very small coupling constant due to a weak equatorial–diaxial coupling. Compounds **36** and **38** were subjected to reductive debenzylation. While, compound **38** could be debenzylated in excellent yield, quinoline derivative **36** could not be debenzylated even in the presence of Pd(OH)_2_.

Consequently, it might be concluded that bulky substituents such as cholesterol and quinoline hindered the complete reductive debenzylation, at least under the described conditions.

### Biology

**Figure 2:**
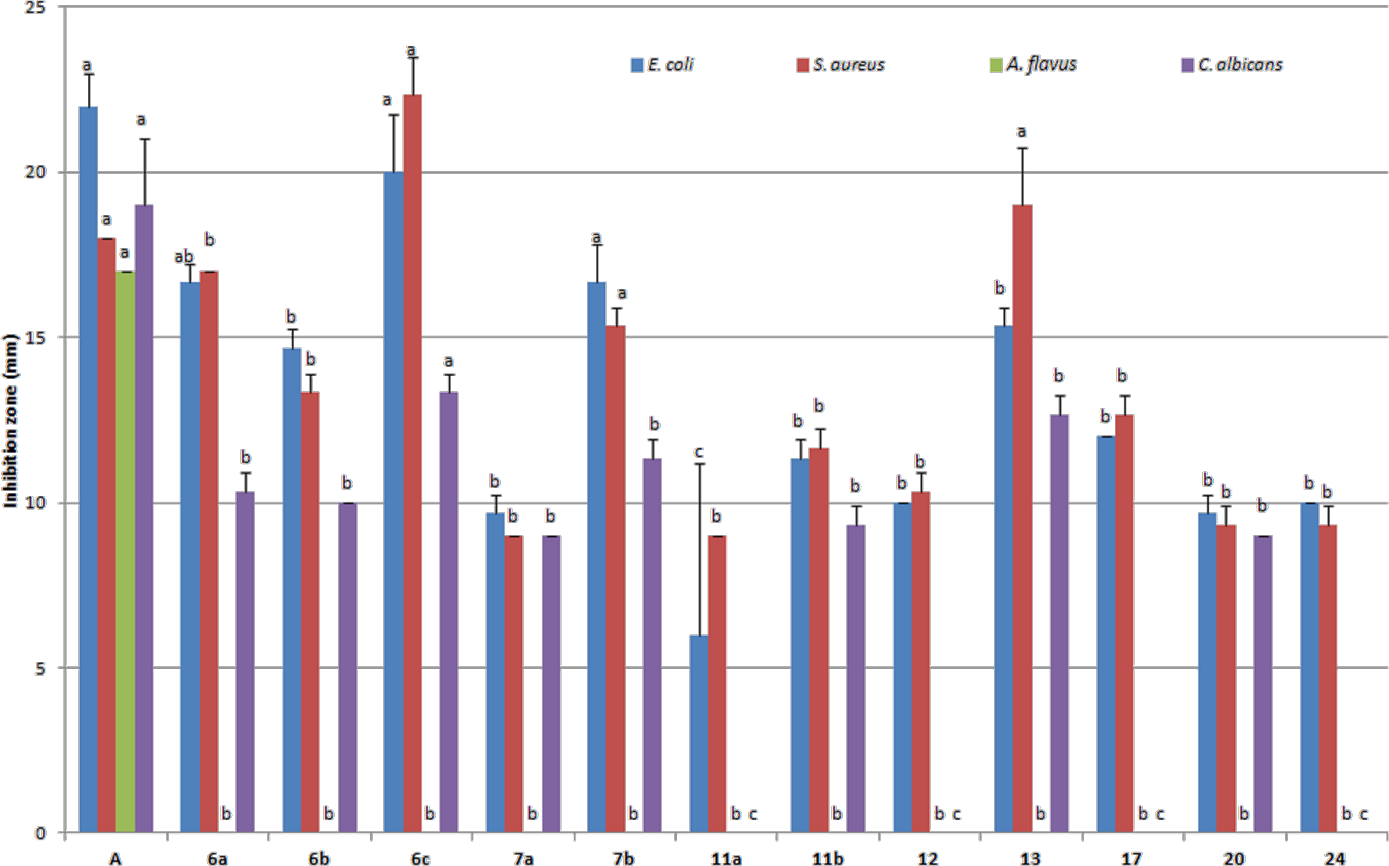
In vitro antimicrobial activity of some new cholesterol derivatives against *E.coli*, *S. aureus*. *A. flavus* and *C. albicans*. Ampicillin was used as positive control (**A**) in the case of *E. coil* and *S. aureus*, while, amphotericin B was used in the case of *A. flavus* and *C. albicans*. Different letters on the column for each parameter varied significantly at *p* ≤ 0.05. These characters (a, b, etc) above the columns denote to statistical variances. Two or more columns with different vertical values but specified with the same character mean that their variation is not significant, i.e., they are equally active.

The antibacterial activity of selected newly synthesized cholesterol conjugates was evaluated in vitro against *E.coli* (ATCC 11775) and *S. aureus* (ATTC 12600) according to the Kirby–Bauer disc diffusion method (Figure 2) [47].

The results shown in Figure 2 revealed that the chalcone modified cholesterol derivatives **6a**,**c** and **7b** were the most potent derivatives against *E. coli*. They were as active as ampicillin and insignificantly varied with each other. Thus, the chalcones possessing unsubstituted phenyl, and *p*-dimethylaminophenyl as well as 2-thienyl alternatives were more active than other congeners against *E. coli.* Despite, derivatives **6b**, **7a**, **11b**, **12**, **13**, **17**, **20** and **24** varied significantly with the control, they insignificantly varied with each other and they were 37–64% less active than ampicillin. Compound **11a**, that is modified with a C11 lipid tail, was the least active cholesterol derivative, thus, it was 73% less active than the control.

On the other hand, derivatives **6c**, **7b** and the bicholesterol **13** were the most active cholesterols against *S. aureus.* All these derivatives insignificantly varied with the control (ampicillin). All other derivatives varied significantly with the control without significant variation among each other. They were 28–56% less active than the control. Therefore, cholesterol–chalcone conjugation could afford derivatives that were as active as ampicillin. However, conjugate **VI** from the previous investigation was still more active than these conjugates [24].

The antifungal activity of selected newly synthesized chalcone conjugates was evaluated in vitro against *A. flavus* (Link) and *Candida albicans* (ATCC 7102) similarly according to the Kirby–Bauer disc diffusion method (Figure 2).

Although, the series was inactive against *A. flavus*, they showed some promising antifungal results against *C. albicans*. As shown in Figure 2, only chalcone **6c** was as active as the control (amphotericin B). Cholesterols **6a**,**b**, **7a**,**b**, **11b**, **13** and **20** significantly varied with the control and they were by 50–71% less active, while the cholesterol derivatives **11a**, **12**, **17** and **24** were entirely inactive.

Therefore, clicked cholesterol–chalcone conjugates could afford, at least, one derivative of promising anticandidal activity which was even better than **VI**.

**Figure 3:**
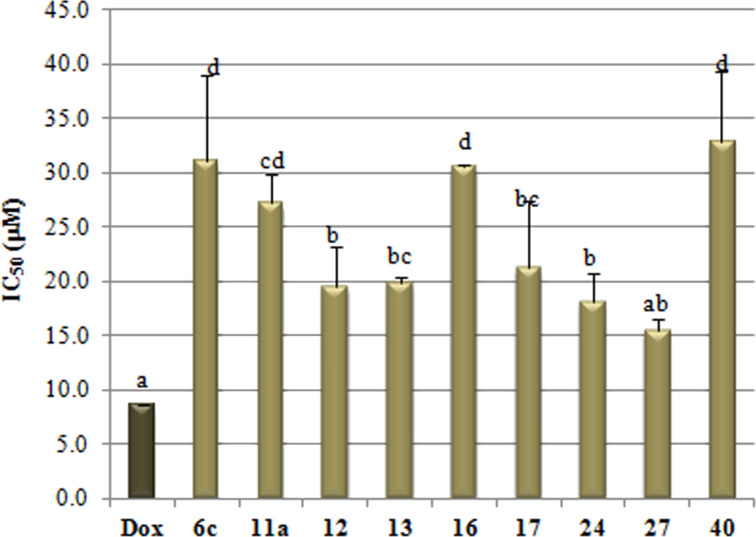
Cytotoxicity effect of some new cholesterol derivatives on the PC3 cell line. Doxorubicin (Dox) was used as positive control. Different letters on the column varied significantly at *p* ≤ 0.05.

A group of target cholesterols were screened in vitro as cytotoxic agents against the human prostate cancer cell line PC3 using the sulforhodamine B colorimetric (SRB) assay and doxorubicin as positive control (IC_50_ = 8.8 μM) (Figure 3) [48].

As shown in Figure 3, cholesterol–lactoside conjugate **27** afforded the best cytotoxicity among this series of compounds without significant variation with the control. While, its analogue hydroxyundecyl analogue **40** was the least cytotoxic conjugate (IC_50_ = 33.5 μM). Thus, this variation showed a potential cytotoxic effect for a cholesterol residue attached to the carbon C-3 of the B ring of the lactose scaffold. On the other hand, modified cholesterols with a chalcone residue (**6c**), a hydroxyhexyl arm (**11a**), and NDMM protected glucosamine tag (**16**) showed low cytotoxicity as triazole **40**. These conjugates showed IC_50_ values of 31.2, 27.1 and 30.3 μM, respectively and they varied insignificantly with each other. Finally, modified cholesterols with a bromohexyl arm (**12**), a Glc*N*Ac residue (**17**), a maltoside tag (**24**) and even the triazole bridged bicholesterol (**13**) showed medium cytotoxic effects within the range of 18.3–21.5 μM and they varied insignificantly with each other.

## Conclusion

In conclusion, cholesterol was successfully converted into 3β-azidocholest-5-ene (**3**) in good yield. This key intermediate, besides 3β-(prop-2-yn-1-yloxy)cholest-5-ene (**10**) were involved in a series of CuAAC reactions to afford a set of new modified cholesterols. The chalcone–triazole–cholesterol derivative **6c** emerged as the most promising antimicrobial probe in this study. It was as active as the controls against *E. coli*, *S. aureus* and *C. albicans*. The cholesterol–triazole–lactoside congener **27** displayed the best in vitro cyctotoxic effect against the prostate cancer PC3 cell line and it showed an activity close to that of the positive control doxorubicin.

## Supporting Information

File 1 Experimental section.
