# Supplementary material for: Synthesis, antimicrobial and cytotoxicity evaluation of new cholesterol congeners
Source: Beilstein J Org Chem. 2015 Oct 16;11:1922–32. doi: 10.3762/bjoc.11.208 (PMC4661006; doi:10.3762/bjoc.11.208)
Supplement: File 1 — Experimental section. [file Beilstein_J_Org_Chem-11-1922-s001.pdf]

Supporting Information

for

Synthesis, antimicrobial and cytotoxicity evaluation of new cholesterol congeners

Mohamed Ramadan El Sayed Aly*[,1,2§], Hosam Ali Saad[1,3] and Shams Hashim Abdel-Hafez[1,4]

Address: [1]Chemistry Department, Faculty of Science, Taif University, 21974-Hawyah-Taif, Kingdom of Saudi Arabia, [2]Chemistry Department, Faculty of Applied Science, Port Said University, 42522-Port Said, Egypt, [3]Chemistry Department, Faculty of Science, Zagazig University, Zagazig, 44511, Egypt and [4]Chemistry Department, Faculty of Science, Assuit University, 71516-Assuit, Egypt

Email: Mohamed Ramadan El Sayed Aly*- mrea34@hotmail.com

*Corresponding author

[§]Tel.: +966 (0)540731939; +20 (0)100 507 3049

Experimental section

Chemistry

Melting points were determined on Electrothermal apparatus and are uncorrected. Flash chromatography was carried out on silica gel (Baker, 30–60 µm). TLC Monitoring tests were carried out using plastic sheets precoated with silica gel 60 F_{245} (layer thickness 0.2 mm) purchased from Merck. Spots were visualized by their fluorescence under UV−lamp (λ = 245 and 366 nm) or staining with iodine vapor, 15% H_2SO_4, $KMnO_4$, or Ce(IV)SO_4 in H_2SO_4. NMR spectra were recorded on Bruker 600 MHz spectrometer, Central Laboratory, King Abd El Aziz University, Jeddah, Saudi Arabia. IR−spectra were recorded on ATR−Alpha FT−IR spectrophotometer 400−4000 cm^{-1}. Mass spectra were recorded on GCMS−QP 1000Ex Shimadzu spectrometers in the Microanalysis Unit at Cairo University.

3α-Bromocholest-5-ene (2)

A mixture of **1** (5.0 g, 12.9 mmol), PPh_3 (4.8 g, 18.3 mmol) in DCM (50 mL) was stirred at ambient temperature, while CBr_4 (5.0 g, 15.0 mmol) was added portionwise and stirring was continued overnight. The mixture was evaporated in vacuo and the residue was purified by flash chromatography (petroleum ether) to afford **2** (4.3 g, 74%) as colorless crystals. R_f 0.67 (petroleum ether); Mp. 72 ºC; ^{1}H NMR (600 MHz, $CDCl_3$): δ 5.92 (dd, 1H, *J* 1.8, 9.6 Hz), 5.59 (dd, 1H, *J* 5.4, 7.2 Hz), 5.39 (t, 1H, *J* 2.4, 4.8 Hz), 2.21–2.14 (m, 2H), 2.11, 2.08 (2t, 1 H, *J* 10.8, 5.4 Hz), 2.03, 2.01 (2t, 1H, *J* 3.6, 6.6 Hz), 1.87−1.81 (m, 1H), 1.80 (dd, 1H, *J* 4.8, 12.6 Hz), 1.69−1.48 (m, 6H), 1.45−1.24 (m, 5H), 1.20–0.98 (m, 10H), 0.95 (s, 3H, CH_3–19), 0.91 (d, 3H, $J_{20,21}$ 6.6 Hz, CH_3–21), 0.87, 0.86 (2d, 6H, $J_{25,26} = J_{25,27}$ 3.0 Hz, CH_3–26, CH_3–27), 0.70 (s, 3H, CH_3–18); ^{13}C NMR (150 MHz, $CDCl_3$): δ 141.48 (C−5_{Chol}), 128.98, 125.05, 123.18, 56.96, 56.15, 48.39, 42.46, 39.81, 39.52, 36.19, 35.81, 35.19, 33.78, 31.78, 31.77, 28.25, 28.02, 24.19, 23.83, 23.05, 22.83, 22.57, 20.96, 18.79, 18.71 (25 C), 11.98 (CH_3–18); EI–MS (*m/z*, %) for $C_{27}H_{45}Br$ (448.27); 449.40 (M+1, 2%), 370.40 (3%), 369.40 (17%), 368.40 (53%).

3β-Azidocholest-5-ene (3)

A mixture of **2** (4.3 g, 9.5 mmol) and NaN_3 (3.0 g, 46.1 mmol) in DMF (25 mL) was stirred at 90−100 ºC for 48h then diluted with H_2O (25 mL). The mixture was extracted with ethyl acetate (3 × 50 mL), dried over Na_2SO_4 and evaporated in vacuo. The residue was purified by flash chromatography (petroleum ether) to afford **3** (2.47 g, 63%) as colorless crystals. R_f 0.26 (petroleum ether); Mp. 98 ºC; IR (ύ, cm^{-1}): 2081 ($N_{3\ str}$); ^{1}H NMR (600 MHz, $CDCl_3$): δ 5.39 (t, 1H, *J* 2.3, 4.8 Hz, H−6_{Chol}), 3.87 (1H, *J* 3.0, 6.0 Hz, H−3_{Chol}), 2.51 (2t, 1H, *J* 2.4, 4.8 Hz), 2.20, 2.17 (2t, 1H, *J* 2.4, 4.8 Hz), 2.03, 2.00 (2t, 1H, *J* 3.0, 3.6, 6.6, 7.2 Hz), 1.99–1.94 (m, 1H), 1.92–1.80 (m, 1H), 1.78, 1.76 (2t, 1H, *J* 3.0, 3.6, 6.6, 7.2 Hz), 1.74–1.66 (m, 1H), 1.68–1.55 (m, 4H), 1.54–1.50 (m, 1H), 1.46–1.33 (m, 5H), 1.27–1.22 (m, 1H), 1.19–0.96 (m, 10H), 1.00 (s, 3H, CH_3–19), 0.91 (d, 3H, $J_{20,21}$ 6.6 Hz, CH_3–21), 0.87, 0.86 (2d, 6H, $J_{25,26} = J_{25,27}$ 2.4 Hz, CH_3–26, CH_3–27), 0.68 (s, 3H, CH_3–18); ^{13}C NMR (150 MHz, $CDCl_3$): δ 138.07 (C−5_{Chol}), 123.17 (C−6_{Chol}), 58.28, 56.58, 56.09, 50.09, 42.30, 39.71, 39.52, 37.09, 36.18, 36.06,

35.81, 33.62, 31.82, 31.77, 28.23, 28.02, 26.09, 24.26, 23.84, 22.84, 22.57, 20.72, 18.99, 18.71 (24 C), 11.86 (CH_3–18); EI–MS (*m/z*, %) for $C_{27}H_{45}N_3$ (411.36); 412.40 (M+1, 2%), 411.40 (M, 4%), 393.40 (2%), 383.40 (49%), 368.40 (78%);

General procedure for the CuAAC reactions.

A mixture of the relevant azido derivative (0.5 mmol), the alkyne (0.45 mmol), $CuSO_4 \cdot 5H_2O$ (0.2 mmol) and L-ascorbic acid (1.4 mol) in THF/H_2O (5:1, 6 mL) was stirred under gentle reflux. The mixture was evaporated in vacuo after 3h and the residue was purified.

(2*E*)-1-[4-({1-[(3β)-Cholest-5-en-3-yl]-1*H*-1,2,3-triazol-4-yl}methoxy)phenyl]-3-phenylprop-2-en-1-one (6a)

Yield: (40%) as colorless powder after flash chromatography using (toluene:ethyl acetate, 12:1);. R_f 0.18 (toluene:ethyl acetate, 12:1); Mp 174–175 ºC; ^{1}H NMR (600 MHz, $CDCl_3$): δ 8.04 (d, 2H, *J* 9.0 Hz, Ar), 7.85 (s, 1H, H–5_{Triaz}), 7.81 (d, 1H, $J_{\alpha,\beta}$ 15.6 Hz, C*H*=CHCO), 7.67–7.64 (m, 2H, Ar), 7.54 (d, 1H, $J_{\alpha,\beta}$ 15.6 Hz, CH=C*H*CO), 7.42–7.37 (m, 3H, Ar), 7.07 (d, 2H, *J* 8.4 Hz, Ar), 5.45 (br. s, 1H, H–6_{Chol}), 5.31 (s, 2H, OCH_2), 4.93 (br.s, 1H), 2.95 (d, 1H, *J* 13.8 Hz), 2.49 (d, 1H, *J* 15.6 Hz), 2.22–2.13 (m, 2H), 2.01–1.97 (m, 2H), 1.85–1.78 (m, 1H), 1.67–1.39 (m, 6H), 1.31–1.21 (m, 4H), 1.11–0.90 (m, 14H), 0.87 (d, 3H, $J_{20,21}$ 6.0 Hz, CH_3–21), 0.86, 0.85 (2d, 6H, $J_{25,26}$ = $J_{25,27}$ 3.0 Hz, CH_3–26, CH_3–27), 0.66 (s, 3H, CH_3–18); ^{13}C NMR (150 MHz, $CDCl_3$): δ 188.40 (C=O), 162.01 (*C*H=CHCO), 144.10, 142.24, 137.91, 135.01, 131.43, 130.79, 130.37, 128.93, 128.38, 124.53, 122.64, 121.61, 114.72 (12 C–Ar, C–4_{Triaz}, C–5_{Chol}, C–5_{Triaz}, C–6_{Chol}, CH=*C*HCO), 62.21, 56.71, 56.52, 56.10, 49.97, 42.24, 39.50, 39.47, 37.05, 36.13, 35.83, 35.41, 32.62, 31.87, 31.62, 28.21, 27.98, 27.18, 24.19, 23.97, 22.82, 22.56, 20.58, 19.25, 18.62 (25 C), 11.81 (CH_3–18); EI–MS (*m/z*, %) for $C_{45}H_{59}N_3O_2$ (673.46): 675.70 (M+2, 4%), 674.70 (M+1, 15%), 673.6 (M, 31%), 663.50 (3%), 422.45 (100%).

(2*E*)-1-[4-({1-[(3β)-Cholest-5-en-3-yl]-1*H*-1,2,3-triazol-4-yl}methoxy)phenyl]-3-(4-methoxyphenyl)prop-2-en-1-one (6b)

Yield: (41%) as colorless powder after flash chromatography using (toluene:ethyl acetate, 7:1). R_f 0.25 (toluene:ethyl acetate, 7:1); Mp. 178–179 ºC; ^{1}H NMR (600 MHz, $CDCl_3$): δ 8.40 (d, 2H, *J* 8.4 Hz, Ar), 7.84 (s, 1H, H–5_{Triaz}), 7.78 (d, 1H, $J_{\alpha,\beta}$ 15.6 Hz, *C*H=CHCO), 7.59 (d, 2H, *J* 8.4 Hz, Ar), 7.42 (d, 1H, $J_{\alpha,\beta}$ 15.6 Hz, CH=*C*HCO), 7.06 (d, 2H, *J* 8.4 Hz, Ar), 6.92 (d, 2H, *J* 8.4 Hz, Ar), 5.44 (d, 1H, *J* 4.2 Hz, H–6_{Chol}), 5.30 (s, 2H, OCH_2), 4.92 (d, 1H, *J* 2.4 Hz), 3.85 (s, 3H, OCH_3), 2.97–2.94 (m, 1H), 2.49 (d, 1H, *J* 16.2 Hz), 2.21–2.19 (d, 1H, *J* 13.8 Hz), 2.16, 2.13, 2.12 (3 t, 1H, *J* 7.2, 3.6 Hz), 2.01–1.97 (m, 2H), 1.84–1.77 (m, 2H), 1.67–1.65 (m, 1H), 1.58–1.300.88 (m, 6H), 1.33–1.20 (m, 5H), 1.12–1.01 (m, 9H), 0.99–0.91 (m, 2H), 0.87 (d, 3H, $J_{20,21}$ 6.0 Hz, CH_3–21), 0.86, 0.85 (2d, 6H, $J_{25,26}$ = $J_{25,27}$ 3.0 Hz, CH_3–26, CH_3–27), 0.65 (s, 3H, CH_3–18); ^{13}C NMR (150 MHz, $CDCl_3$): δ 188.44 (C=O), 161.83, 161.53, 143.94, 142.31, 137.91, 131.71, 130.67, 130.14, 127.76, 124.54, 122.63, 119.28, 114.65, 114.38 (12 C–Ar, C–4_{Triaz}, C–5_{Chol}, C–5_{Triaz}, C–6_{Chol}, *C*H=*C*HCO), 62.21, 56.71, 56.51, 56.09, 55.39, 49.97, 42.25, 39.05, 39.47, 37.05, 36.13, 35.84, 35.41, 32.62, 31.86, 31.62, 28.21, 27.99, 27.18, 24.20, 24.00, 22.82, 22.57, 20.58, 19.25, 18.62 (26 C), 11.82 (CH_3–18); EI–MS (*m/z*, %) for $C_{46}H_{61}N_3O_3$ (703.47): 705.60 (M+2, 7%), 704.65 (M+1, 23%), 703.65 (M, 41%), 688.40 (3%), 422.45 (100%).

(2*E*)-1-[4-({1-[(3β)-Cholest-5-en-3-yl]-1*H*-1,2,3-triazol-4-yl}methoxy)phenyl]-3-[4-(dimethylamino)phenyl]prop-2-en-1-one (6c)

Yield: (68%) as fluorescent green mass after flash chromatography (toluene:ethyl acetate, 7:1). R_f 0.19 (toluene:ethyl acetate, 7:1); Mp. 168 ºC; ^{1}H NMR (600 MHz, $CDCl_3$): δ 8.01 (d, 2H, *J* 8.4 Hz, Ar), 7.82 (s, 1H, H–5_{Triaz}), 7.77 (d, 1H, $J_{\alpha,\beta}$ 15.6 Hz, *C*H=CHCO), 7.53 (d, 2H, *J* 9.0 Hz, Ar), 7.33 (d, 1H, $J_{\alpha,\beta}$ 15.6 Hz, CH=*C*HCO), 7.04 (d, 2H, *J* 9.0 Hz, Ar), 6.67 (d, 2H, *J* 9.0 Hz, Ar), 5.43 (br.s, 1H, *J* 4.8 Hz, H–6_{Chol}), 5.28 (s, 2H, OCH_2), 4.91 (br.d, 1H, *J* 2.4 Hz), 3.03 (s, 6H, NMe_2), 2.95 (s, 1H), 2.48 (d, 1H, *J* 16.2 Hz), 2.18–2.09 (m, 2H), 2.00–1.95 (m, 2H), 1.84–1.79 (m, 1H), 1.67–1.63 (m, 1H), 1.56–1.39 (m, 5H), 1.32–1.21 (m, 4H), 1.11–0.88 (m, 14H), 0.86 (d, 3H, $J_{20,21}$ 6.6 Hz, CH_3–21),

0.85, 0.84 (2d, 6H, $J_{25,26}$ = $J_{25,27}$ 3.0 Hz, CH_3–26, CH_3–27), 0.64 (s, 3H, CH_3–18); ^{13}C NMR (150 MHz, $CDCl_3$): δ 188.64 (C=O), 161.55 (*C*H=CHCO), 151.95, 145.12, 137.93, 132.27, 130.54, 130.33, 124.59, 122.85, 122.63, 116.45, 114.56, 111.86 (12 C–Ar, C–4_{Triaz}, C–5_{Chol}, C–5_{Triaz}, C–6_{Chol}, CH=*C*HCO), 62.22, 56.74, 56.51, 56.10, 49.98, 42.28, 40.17, 39.51, 37.09, 36.15, 35.88, 35..45, 32.65, 31.89, 31.67, 28.25, 28.03, 27.22, 24.24, 24.05, 22.85, 22.60, 20.61, 19.28, 18.65 (27 C), 11.84 (CH_3–18); EI–MS (*m/z*, %) for $C_{47}H_{64}N_4O_2$ (716.50): 717.40 (M+1, 13%), 716.40 (M, 19%), 703.40 (28%), 422.40 (88%).

(2*E*)-1-[4-({1-[(3β)-Cholest-5-en-3-yl]-1*H*-1,2,3-triazol-4-yl}methoxy)phenyl]-3-(2-furyl)prop-2-en-1-one (7a)

Yield: (47%) as yellow crystals upon flash chromatography (toluene:ethyl acetate, 4:1). R_f 0.33 (toluene:ethyl acetate, 4:1); Mp. 162–164 ºC; ^{1}H NMR (600 MHz, $CDCl_3$): δ 8.04 (d, 2H, *J* 9.0 Hz, H–3_{Ar}, H–5_{Ar}), 7.84 (s, 1H, H–5_{Triaz}), 7.58 (d, 1H, $J_{\alpha,\beta}$ 15.0 Hz, C*H*=CHCO), 7.52 (d, 1H, $J_{4,5}$ 1.8 Hz, H–5_{Fur}), 7.46 (d, 1H, $J_{\alpha,\beta}$ 15.0 Hz, CH=C*H*CO), 7.06 (d, 2H, *J* 9.0 Hz, H–2_{Ar}, H–6_{Ar}), 6.70 (d, 1H, $J_{3,4}$ 3.0 Hz, H–3_{Fur}), 6.15 (dd, 1H, $J_{3,4}$ 3.0, $J_{4,5}$ 1.8 Hz, H–4_{Fur}), 5.44 (br.d, 1H, *J* 4.8 Hz, H–6_{Chol}), 5.31 (s, 2H, OCH_2), 4.93 (br.d, 1H, *J* 3.0 Hz), 2.97–2.94 (m, 1H), 2.51–2.48 (m, 1H), 2.21–2.19 (m, 1H), 2.16–2.10 (3t, 1H, *J* 3.6, 4.2 Hz), 2.05–1.95 (m, 2H), 1.85–1.79 (m, 1H), 1.68–1.65 (m, 2H), 1.60–1.39 (m, 5H), 1.37–1.21 (m, 5H), 1.16–1.00 (m, 10H), 0.99–0.82 (m, 2H), 0.88 (d, 3H, $J_{20,21}$ 7.8 Hz, CH_3–21), 0.87, 0.86 (2d, 6H, $J_{25,26}$ = $J_{25,27}$ 3.0 Hz, CH_3–26, CH_3–27), 0.66 (s, 3H, CH_3–18); ^{13}C NMR (150 MHz, $CDCl_3$): δ 187.85 (C=O), 161.98 (*C*H=CHCO), 151.81, 144.72, 142.30, 137.92, 131.45, 130.73, 130.11, 124.56, 122.62, 119.02, 115.91, 114.70, 112.61 (6 C–Ar, 4 C–Fur, CH=*C*HCO, C–4_{Triaz}, C–5_{Chol}, C–5_{Triaz}, C–6_{Triaz}), 62.22, 56.72, 56.52, 56.09, 49.99, 42.26, 39.51, 39.49, 37.07, 36.14, 35.85, 35.42, 32.62, 31.87, 31.63, 28.21, 28.00, 27.20, 24.20, 23.99, 22.83, 22.57, 20.58, 19.26, 18.63 (25 C), 11.83 (CH_3–18); EI–MS (*m/z*, %) for $C_{43}H_{57}N_3O_3$ (663.44): 665.60 (M+2, 4%), 664.60 (M+1, 15%), 663.60 (M, 29%), 449.50 (10%), 422.50 (100%).

(2*E*)-1-[4-({1-[(3β)-Cholest-5-en-3-yl]-1*H*-1,2,3-triazol-4-yl}methoxy)phenyl]-3-(2-thienyl)prop-2-en-1-one (7b)

Yield: (60%) as yellowish powder upon flash chromatography (toluene:ethyl acetate, 6:1). R_f 0.35 (toluene:ethyl acetate, 4:1); Mp 172–174 ºC; ^{1}H NMR (600 MHz, $CDCl_3$): δ 8.02 (d, 2H, J 9.0 Hz, H–3_{Ar}, H–5_{Ar}), 7.93 (d, 1H, $J_{\alpha,\beta}$ 15.6 Hz, C*H*=CHCO), 7.84 (s, 1H, H–5_{Triaz}), 7.40 (d, 1H, $J_{4,5}$ 4.8 Hz, H–5_{Thioph}), 7.35 (d, 1H, $J_{\alpha,\beta}$ 15.6 Hz, CH=C*H*CO), 7.34 (d, 1H, $J_{3,4}$ 3.6 Hz, H–3_{Thioph}), 7.08 (dd, 1H, $J_{3,4}$ 3.6, $J_{4,5}$ 4.8 Hz, H–4_{Thioph}), 7.07 (d, 2H, J 9.0 Hz, H–2_{Ar}, H–6_{Ar}), 5.44 (br.d, 1H, J 4.8 Hz, H–6_{Chol}), 5.31 (s, 2H, OCH_2), 4.93 (br.d, 1H, J 2.4 Hz), 2.95 (br.dd, 1H, J 2.4, 16.2 Hz), 2.49 (br.d, 1H, J 15.6 Hz), 2.24–2.18 (m, 1H), 2.16, 2.14, 2.11 (3t, 1H, J 3.6, 7.2 Hz), 2.33–1.96 (m, 2H), 1.85–1.79 (m, 1H), 1.75–1.70 (m, 1H), 1.66 (br.d, 1H, J 13.2 Hz), 1.58–1.38 (m, 6H), 1.37–1.28 (m, 3H), 1.27–1.20 (m, 1H), 1.14–1.01 (m, 9H), 0.98–0.82 (m, 3H), 0.87 (d, 3H, $J_{20,21}$ 6.6 Hz, CH_3–21), 0.87, 0.86 (2d, 6H, $J_{25,26}$ = $J_{25,27}$ 3.0 Hz, CH_3–26, CH_3–27), 0.66 (s, 3H, CH_3–18); ^{13}C NMR (150 MHz, $CDCl_3$): δ 187.80 (C=O), 161.99 (*C*H=CHCO), 140.55, 137.91, 136.55, 131.86, 131.35, 130.69, 128.50, 128.30, 124.55, 122.64, 120.39, 114.72 (6 C–Ar, 4 C–Thioph, CH=*C*HCO, C–4_{Triaz}, C–5_{Chol}, C–5_{Triaz}, C–6_{Triaz}), 62.22, 56.72, 56.51, 56.10, 49.97, 42.25, 39.50, 39.48, 37.06, 36.13, 35.84, 35.41, 32.62, 31.87, 31.62, 28.22, 27.99, 27.19, 24.20, 23.99, 22.83, 22.57, 20.58, 19.25, 18.62 (25 C), 11.82 (CH_3–18); EI–MS (*m/z*, %) for $C_{43}H_{57}N_3O_2S$ (679.42): 681.50 (M+2, 4%), 680.50 (M+1, 9%), 679.50 (M, 19%), 570.50 (3%), 422.50 (66%).

6-(4-{[(3β)-Cholest-5-en-3-yloxy]methyl}-1*H*-1,2,3-triazol-1-yl)hexan-1-ol (11a)

Yield: (90%) as creamy foam upon flash chromatography (toluene:acetone, 7:3). R_f 0.34 (toluene:acetone, 7:3); IR (ύ, cm^{-1}): 3345 (OH_{str}), 1071, 1023 (C–O–C_{str}); ^{1}H NMR (600 MHz, $CDCl_3$): δ 7.53 (s, 1H, H–5_{Triaz}), 5.35 (dd, 1H, J 2.4, 3.0 Hz, H–6_{Chol}), 4.68 (s, 2H, OCH_2), 4.34 (t, 2H, J 7.2 Hz, CH_2–1_{Hex}), 3.63 (t, 2H, J 6.6 Hz, CH_2–6_{Hex}), 3.35–3.30 (m, 1H, H–3_{Chol}), 2.42–2.39 (m, 1H), 2.27–2.23 (dd, 1H, J 11.4, 10.8 Hz), 2.02–1.81 (m, OH, 9H), 1.60−1.32 (m, 17H), 1.27–1.23 (m, 1H), 1.20–1.01 (m, 7H), 1.00 (s, 3H, CH_3–19_{Chol}), 0.91 (d, 3H, J 6.6 Hz, CH_3–21_{Chol}), 0.87, 0.86 (2d, 6H, J 3.0 Hz, CH_3–26_{Chol}, CH_3–27_{Chol}), 0.67 (s, 3H, CH_3–18_{Chol}); ^{13}C NMR (150 MHz, $CDCl_3$): δ

145.96 (C–4_{Triaz}), 140.67 (C–5_{Chol}), 122.13, 121.84 (C–5_{Triaz}, C–6_{Chol}), 78.96 (C–3_{Chol}), 62.54, 61.69 (2 OCH_2), 56.75, 56.14 (C–14_{Chol}, C–17_{Chol}), 50.17, 50.15, 42.32, 39.77, 39.52, 39.03, 37.16, 36.85, 36.18, 35.79, 32.33, 31.94, 31.88, 30.19, 28.31, 28.23, 28.02, 26.11, 25.08 (19 C), 24.29 (CH_2–15_{Chol}), 23.82 (CH_2–23_{Chol}), 22.83, 22.57 (CH_3–26_{Chol}, CH_3–27_{CHol}), 21.07 (CH_2–11_{Chol}), 19.37 (CH_3–19_{Chol}), 18.72 (CH_3–21_{Chol}), 11.86 (CH_3–18_{Cho5l}); EI–MS (*m/z*, %) for $C_{36}H_{61}N_3O_2$ (567.48): 568.10 (M+1, 46), 567.10 (M, 48), 564.10 (26), 555.10 (62.8), 505.10 (58.4).

11-(4-{[(3β)-Cholest-5-en-3-yloxy]methyl}-1*H*-1,2,3-triazol-1-yl)undecan-1-ol (11b)

Yield: (67%) as colorless mass upon flash chromatography (toluene:ethyl acetate, 3:2). R_f 0.25 (toluene:ethyl acetate, 3:2); Mp 94 ºC; ^{1}H NMR (600 MHz, $CDCl_3$): δ 7.52 (s, 1H, H–5_{Triaz}), 5.35 (dd, 1H, *J* 2.4, 3.0 Hz, H–6_{Chol}), 4.68 (s, 2H, OCH_2), 4.32 (t, 2H, *J* 7.8 Hz, CH_2–1_{Hex}), 3.63 (t, 2H, $J_{10,11}$ 3, 6.6, J_{gem} 10.2 Hz, CH_2–11_{Hex}), 3.35–3.30 (m, 1H, H–3_{Chol}), 2.42–2.39 (m, 1H), 2.27–2.22 (dd, 1H), 2.03–1.79 (m, OH, 8H), 1.60–1.43 (m, 9H), 1.34–1.26 (m, 19H), 1.18–1.04 (m, 6H), 1.03–0.96 (m, 2H), 1.00 (s, 3H, CH_3–19_{Chol}), 0.91 (d, 3H, *J* 6.6 Hz, CH_3–21_{Chol}), 0.87, 0.86 (2d, 6H, *J* 2.4, 3.0 Hz, CH_3–26_{Chol}, CH_3–27_{Chol}), 0.67 (s, 3H, CH_3–18_{Chol}); ^{13}C NMR (150 MHz, $CDCl_3$): δ 145.90 (C–4_{Triaz}), 140.66 (C–5_{Chol}), 122.05, 121.82 (C–5_{Triaz}, C–6_{Chol}), 78.90 (C–3_{Chol}), 62.98, 61.71 (2 OCH_2), 56.75, 56.14 (C–14_{Chol}, C–17_{Chol}), 50.33, 50.14, 42.31, 39.76, 39.51, 39.04, 37.16, 36.84, 36.18, 35.78, 32.76, 31.93, 31.88, 30.26, 29.47, 29.35, 29.34, 29.29, 28.92, 28.31, 28.23, 28.01, 26.44, 25.71 (24–C), 24.29 (CH_2–15_{Chol}), 23.82 (CH_2–23_{Chol}), 22.82, 22.57 (CH_3–26_{Chol}, CH_3–27_{CHol}), 21.06 (CH_2–11_{Chol}), 19.37 (CH_3–19_{Chol}), 18.71 (CH_3–21_{Chol}), 11.86 (CH_3–18_{Chol}). $C_{41}H_{71}N_3O_2$ (638.02)

(3β)-3-{[1-(6-Bromohex-1-yl)-1*H*-1,2,3-triazol-4-yl]methoxy}cholest-5-ene (12)

A mixture of **11a** (0.4 g, 0.7 mmol), PPh_3 (0.25 g, 0.9 mmol) in DCM (5 mL) was treated with CBr_4 (0.35 g, 1.0 mmol) and stirred at ambient temperature overnight. The reaction mixture was evaporated in vacuo and the residue was purified by flash chromatography (toluene:acetone, 7:1) to afford **12** (0.26 g, 59%) as faint greenish crystals. R_f 0.56 (toluene:acetone, 4:1); Mp. 126 ºC; ^{1}H NMR (600 MHz, $CDCl_3$): δ 7.55 (s, 1H, H–5_{Triaz}),

5.35 (dd, 1H, *J* 2.4 Hz, H–6_{Chol}), 4.70 (s, 2H, OCH_2), 4.35 (t, 2H, *J* 7.2 Hz, CH_2–1_{Hex}), 3.39 (t, 2H, *J* 6.6, J_{gem} 10.2 Hz, CH_2–6_{Hex}), 3.34–3.31 (m, 1H, H–3_{Chol}), 2.42–2.39, 2.27–2.23 (2m, 2H), 2.02–1.82 (m, 10H), 1.53–1.33 (m, 16H), 1.27–1.23 (m, 1H, *J* 4.2, 3.0 Hz), 1.16–1.05 (m, 7H), 1.00 (s, 3H, CH_3–19_{Chol}), 0.91 (d, 3H, $J_{20,21}$ 6.8 Hz, CH_3–21_{Chol}), 0.87, 0.86 (2d, 6H, *J* 2.4, 3.0 Hz, CH_3–26_{Chol}, CH_3–27_{Chol}), 0.67 (s, 3H, CH_3–18_{Chol}); ^{13}C NMR (150 MHz, $CDCl_3$): δ 145.71 (C–4_{Triaz}), 140.65 (C–5_{Chol}), 122.31, 121.85 (C–5_{Triaz}, C–6_{Chol}), 78.98 (C–3_{Chol}), 61.64 (OCH_2), 56.75, 56.14 (C–14_{Chol}, C–17_{Chol}), 50.22, 50.14, 42.32, 39.77, 39.52, 39.04, 37.16, 36.85, 36.18, 35.79, 33.55, 32.37, 31.94, 31.88, 30.09, 28.31, 28.23, 28.02, 27.49, 25.65 (20C), 24.29 (CH_2–15_{Chol}), 23.81 (CH_2–23_{Chol}), 22.83, 22.57 (CH_3–26_{Chol}, CH_3–27_{CHol}), 21.06 (CH_2–11_{Chol}), 19.37 (CH_3–19_{Chol}), 18.72 (CH_3–21_{Chol}), 11.86 (CH_3–18_{Chol}); EI–MS (*m/z*, %) for $C_{36}H_{60}BrN_3O$ (629.39): 630.00 (M+1, 55%), 629.00 (M, 64%), 623.00 (43%); 615.00 (47%), 584 (58%).

(3β)-3-(4-{[(3β)-Cholest-5-en-3-yloxy]methyl}-1*H*-1,2,3-triazol-1-yl)cholest-5-ene (13)

Yield: (96%) as amorphous colorless mass upon flash chromatography (toluene:ethyl acetate, 7:1). R_f 0.32 (toluene:ethyl acetate, 7:1); Mp 192–194 ºC; ^{1}H NMR (600 MHz, $CDCl_3$): δ 7.78 (s, 1H, H–5_{Triaz}), 5.50 (br.t, 1H, *J* 2.4, 4.8 Hz, H–6_{Chol}), 5.33 (br.t, 1H, *J* 3.0, 5.4 Hz, H–6_{Chol}), 4.89 (br.d, 1H, *J* 1.8 Hz), 4.70 (d, 2H, *J* 3.0 Hz, OCH_2), 3.28–3.24 (m, 1H), 2.94 (br.d, 1H, *J* 14.4 Hz), 2.52 (br.d, 1H, *J* 15.6 Hz), 2.40–2.38, 2.37–2.37 (2m, 1H), 2.25–2.20 (m, 2H), 2.14, 2.12, 2.10 (3 t, 1H, *J* 3.6, 2.7 Hz), 2.07–1.93 (m, 4H), 1.91–1.79 (m, 4H), 1.67–1.40 (m, 16H), 1.39–1.21 (m, 9H), 1.18–1.04 (m, 15H), 1.01–0.95 (m, 7H), 0.94–0.88 (m, 1H), 0.91, 0.89 (2d, 6H, $J_{20,21}$ 6.6 Hz, 2 CH_3–21), 0.86–0.85 (m, 12 H, 2CH_3–26, 2CH_3–27), 0.68, 0.67 (2s, 6H, 2 CH_3–18); ^{13}C NMR (150 MHz, $CDCl_3$): δ 145.00, 140.62, 138.17 (C–4_{Triaz}, 2 C–5_{Chol}), 124.37, 122.04, 121.86 (C–5_{Triaz}, 2 C–6_{Chol}), 78.46, 61.90, 56.74, 56.50, 56.34, 56.17, 50.20, 50.09, 42.34, 42.32, 39.86, 39.63, 39.53, 39.51, 39.00, 37.40, 37.06, 36.86, 37.06, 36.86, 36.24, 36.20, 35.91, 35.82, 35.40, 32.87, 31.96, 31.91, 31.67, 28.69, 28.27, 28.26, 28.04, 28.03, 27.26, 24.31,

24.24, 24.07, 23.86, 22.84, 22.83, 22.61, 22.59, 21.10, 20.64, 19.42, 19.31, 18.75, 18.66 (49 C), 11.88 (2 CH_3–18); $C_{57}H_{93}N_3O$ (835.73).

(3β)-Cholest-5-en-3-yl 3,4,6-tri-*O*-acetyl-2-deoxy-2-dimethylmaleimido-β-D-glucopyranoside (15)

A mixture of **1** (0.4 g, 1.0 mmol) and **14** (0.7 g, 1.2 mmol) in CH_3CN (5 mL) was stirred under N_2 at ambient temperature then TMSOTf (0.01 M, 1.0 mL, 1.0 mol %) was added dropwise. Stirring was continued for 15 min then Et_3N was added and the mixture was evaporated in vacuo. The residue was purified by flash chromatography (toluene:ethyl acetate, 6:1) and the fractions including the product were collected and acetylated (Pyr/Ac_2O, 2:1, 3 mL) then worked up and purified again under the same conditions. This acetylation removed the unreacted cholesterol and easily afforded pure **15** (0.6 g, 74%) as colorless crystals. R_f 0.46 (toluene:ethyl acetate, 4:1); Mp. 94 ºC; ^{1}H NMR (600 MHz, $CDCl_3$): δ 5.60 (dd, 1H, $J_{2,3}$ 10.8, $J_{3,4}$ 9.0 Hz, H–3_{Glu}), 5.31–5.29 (m, 1H, H–6_{Chol}), 5.30 (d, 1H, $J_{1,2}$ 8.4 Hz, H–1_{Glu}), 5.09 (dd, 1H, $J_{3,4}$ 9.0, $J_{4,5}$ 9.6 Hz, H–4_{Glu}), 4.29 (dd, 1H, J_{gem} 12.0, $J_{5,6}$ 4.8 Hz, H–6_{Glu}), 4.11 (dd, 1H, J_{gem} 12.0, $J_{5,6`}$ 2.4 Hz, H–6`$_{Glu}$), 4.05 (dd, 1H, $J_{1,2}$ 8.4, $J_{2,3}$ 10.8 Hz, H–2_{Glu}), 3.81–3.78 (2m, 1H, H–5_{Glu}), 3.47–3.43 (m, 1H, H–3_{Chol}), 2.09, 2.02, 1.96, 1.92 (4s, 15H, 3 $COCH_3$, 2 CH_3), 2.07–1.93 (m, 7H), 1.84–1.80 (m, 3H), 1.55–1.32 (m, 11 H), 1.28–1.22 (m, 1H), 1.14–0.96 (m, 6H), 0.94 (s, 3H, CH_3–19_{Chol}), 0.90 (d, 3H, J 6.8 Hz, CH_3–21_{Chol}), 0.86, 0.85 (2d, 6H, J 2.4, 3.0 Hz, CH_3–26_{Chol}, CH_3–27_{Chol}), 0.66 (s, 3H, CH_3–18_{Chol}); ^{13}C NMR (150 MHz, $CDCl_3$): δ 170.84, 170.30, 169.56, 163.76 (5C=O), 140.22 (C–5_{Chol}), 137.38 (C=$C_{Maleimido}$), 122.17 (C–6_{Chol}), 96.86 (C–1_{Glu}), 79.56 (C–3_{Chol}), 71.58 (C–5_{Glu}), 71.01 (C–3_{Glu}), 69.06 (C–4_{Glu}), 62.18 (C–6_{Glu}), 56.70, 56.11 (C–14_{Chol}, C–17_{Chol}), 54.68 (C–2_{Glu}), 50.07, 42.29, 39.71, 39.50, 38.69, 37.11, 36.62, 36.16, 35.76, 31.89, 31.82, 29.35, 28.21, 28.00 (14C), 24.27 (CH_2–15_{Chol}), 23.80 (CH_2–23_{Chol}), 22.82, 22.56 (CH_3–26_{Chol}, CH_3–27_{CHol}), 21.01 (CH_2–11_{Chol}), 20.81, 20.66, 20.56 (3 *C*H_3CO), 19.37 (CH_3–19_{Chol}), 18.70 (CH_3–21_{Chol}), 11.84 (CH_3–18_{Chol}), 8.84 ($2CH_{3Maleimid}$); EI–MS (*m/z*, %) for $C_{45}H_{67}NO_{10}$ (781.48): 783.00 (M+2, 26%), 782.00 (M+1, 24%), 781.00 (M, 44%), 755.00 (35%), 711 (41%).

(3β)-Cholest-5-en-3-yl 2-deoxy-2-dimethylmaleimido-β-D-glucopyranoside (16)

A mixture of **15** (0.23 g, 0.3 mmol) and NaOMe/MeOH (0.005 M, 20 mL) was stirred at ambient temperature for 75 min then neutralized with Amberlite IR 120 (H^+) resin, filtered and evaporated in vacuo. The residue was purified by flash chromatography using solvent gradient of toluene then (toluene:acetone 5:4) to afford **16** (0.16 g, 84%) as colorless mass. R_f 0.17 (toluene:acetone 5:4); Mp. 220 ºC; IR (ύ, cm^{-1}): 3524–3461 ($O–H_{str}$), 1701 ($C=O_{str}$); ^1H NMR (600 MHz, MeOH–d_4): δ 5.28–5.28 (m, 1H, $H–6_{Chol}$), 5.10 (d, 1H, $J_{1,2}$ 8.4 Hz, $H–1_{Glu}$), 4.05 (dd, 1H, $J_{1,2}$ 8.4, $J_{2,3}$ 10.8 Hz, $H–2_{Glu}$), 3.87 (dd, 1H, J_{gem} 12.0, $J_{5,6}$ 2.4 Hz, $H–6_{Glu}$), 3.70–3.67 (m, $2H_{Glu}$), 3.49–3.44 (m, 1H, $H–3_{Chol}$), 3.35–3.30 (m, 4H), 2.10–2.09, 2.08–2.07 (2m, 1H), 2.04–2.01 (m, 1H, J 3.6, 7.2 Hz), 1.96 (s, 6H, $2CH_{3Maleimide}$), 1.95–1.81 (m, 4H), 1.62–1.58 (m, 1H), 1.53–1.33 (m, 9H), 1.29–1.24 (m, 1H), 1.18–0.97 (m, 9H), 0.94 (s, 3H, $CH_3–19_{Chol}$), 0.92 (d, 3H, J 6.6 Hz, $CH_3–21_{Chol}$), 0.87, 0.86 (2d, 6H, J 2.4 Hz, $CH_3–26_{Chol}$, $CH_3–27_{Chol}$), 0.69 (s, 3H, $CH_3–18_{Chol}$); ^{13}C NMR (150 MHz, MeOH–d_4): δ 141.55 ($C–5_{Chol}$), 138.40 ($C=C_{Maleimid}$), 123.05 ($C–6_{Chol}$), 98.16 ($C–1_{Glu}$), 79.88, 78.25, 72.90, 72.55, 62.75, 58.56, 58.16, 57.57, 51.68, 43.51, 41.15, 40.71, 39.99, 38.44, 37.80, 37.39, 37.14, 33.23, 33.03, 30.54, 29.34, 29.18, 25.32, 24.96, 23.21, 22.95, 22.16 (27 C), 19.80, 19.24 ($CH_3–19_{Chol}$, $CH_3–21_{Chol}$), 12.31 (CH_3–18), 8.80 (2 $CH_{3\ Maleimide}$); EI–MS (*m/z*, %) for $C_{39}H_{61}NO_7$ (655.44): 651.00 (M–4, 23%), 634.00 (26%), 604.00 (26%), 502.00 (30%).

(3β)-Cholest-5-en-3-yl 2-Acetamido-2-deoxy-β-D-glucopyranoside (17)

A mixture of **16** (0.1 g, 0.15 mmol) and NaOH (0.2 g, 5.0 mmol) in dioxane/MeOH/H_2O (6:3:1, 10 mL) was stirred at ambient temperature for 8 h, then the acidity of the mixture was adjusted at pH 5 by 1 N HCl and stirring was continued overnight. The mixture was neutralized by Na_2CO_3 then evaporated in vacuo. The residue was stirred with Pyr/Ac_2O (2:1, 5 mL) overnight then evaporated in vacuo and the residue was stirred with NaOMe/MeOH (0.005 M, 20 mL) for 2 h. The mixture was neutralized with Amberlite IR (H^+) resin, filtered and evaporated in vacuo. The residue was purified by flash chromatography (toluene:acetone, 5:4) to afford **17** (30 mg, 37%) as amorphous colorless mass. R_f 0.17 (toluene:acetone 5:4); ^1H NMR (600 MHz, MeOH–d_4): δ 5.28–5.27 (m,

1H, H−6$_{Chol}$), 5.10 (d, 1H, $J_{1,2}$ 8.4 Hz, H−1$_{Glu}$), 4.05 (dd, 1H, $J_{1,2}$ 8.4, $J_{2,3}$ 10.8 Hz, H−2$_{Glu}$), 3.87 (dd, 1H, J_{gem} 12.0, $J_{5,6}$ 2.4 Hz, H−6$_{Glu}$), 3.70−3.67 (m, 2H), 2.10−2.09, 2.08−2.07 (2m, 1H), 2.03−2.01 (m, 1H), 1.96 (s, 3H, $COCH_3$), 1.95−1.81 (m, 6H), 1.60−1.25 (m, 13H), 1.18−0.99 (m, 10 H), 0.94 (s, 3H, CH_3−19), 0.92 (d, 3H, $J_{20,21}$ 6.0 Hz, CH_3−21), 0.87, 0.86 (2d, 6H, $J_{25,26}$ = $J_{25,27}$ 2.4 Hz, CH_3−26, CH_3−27), 0.69 (s, 3H, CH_3−18); ^{13}C NMR (150 MHz, $CDCl_3$): δ 183.10 (*C*OCH$_3$), 141.64 (C−5$_{Chol}$), 123.06 (C−6$_{Chol}$), 98.15 (C−1$_{Glu}$), 79.87, 78.25, 72.90, 72.54, 58.56, 58.16, 57.57, 51.68, 43.50, 41.14, 40.71, 39.99, 38.44, 37.80, 37.38, 37.14, 33.23, 33.03, 30.54, 29.34, 29.18, 25.32, 24.95, 23.20, 22.95, 22.16, 19.80, 19.24 (30 C), 12.30 (CH_3−18); EI–MS (*m/z*, %) for $C_{35}H_{59}NO_6$ (589.43): 590.00 (M+1, 30%), 589.00 (M, 30%), 572.00 (40%), 514.00 (43%).

6-Azidohex-1-yl 3,4,6-tri-*O*-acetyl-2-deoxy-2-dimethylmaleimido-β-D-glucopyranoside (18)

A mixture of **14** (0.7 g, 1.2 mmol) and **9a** (0.15 g, 1.0 mmol) in DCM (5 mL) was stirred under N_2 then treated with TMSOTf (0.01 M, 1.2 mL) and stirring was continued for 45 min. The mixture was neutralized with Et_3N and evaporated in vacuo. The residue was purified by flash chromatography (toluene:ethyl acetate, 4:1) to afford **18** (0.4 g, 71%) as syrup. R_f 0.5 (toluene:ethyl acetate, 2:1); IR (ύ, cm^{-1}): 2096 (N_{3str}), 1714 (C=O$_{str}$), 1223, 1034 (C–O–C$_{str}$); ^{1}H NMR (600 MHz, $CDCl_3$): δ 5.59 (dd, 1H, $J_{2,3}$ 10.2, $J_{3,4}$ 9.0 Hz, H−3$_{Glu}$), 5.18 (d, 1H, $J_{1,2}$ 8.4 Hz, H−1$_{Glu}$), 5.10 (dd, 1H, $J_{3,4}$ 9.0, $J_{4,5}$ 9.6 Hz, H−4$_{Glu}$), 4.29 (dd, 1H, J_{gem} 12.0, $J_{5,6}$ 4.8 Hz, H−6$_{Glu}$), 4.13 (dd, 1H, J_{gem} 12.0, $J_{5,6`}$ 2.4 Hz, H−6`$_{Glu}$), 4.05 (dd, 1H, $J_{1,2}$ 8.4, $J_{2,3}$ 10.2 Hz, H−2$_{Glu}$), 3.81–3.77 (m, 2H, CH_2−1$_{Hex}$), 3.41 (m, 1H, H−5$_{Glu}$), 3.21 (dd, 2H, J 7.2, 6.6 Hz, CH_2−6$_{Hex}$), 2.09, 2.01, 1.91 (3s, 9H, 3 $COCH_3$), 1.95 (s, 6H, 2 CH_3), 1.52–1.49 (m, 4H, CH_2−2$_{Hex}$, CH_2−5$_{Hex}$), 1.30–1.21 (m, 4H, CH_2−3$_{Hex}$, CH_2−4$_{Hex}$); ^{13}C NMR (150 MHz, $CDCl_3$): δ 170.79, 170.26, 169.52 (5 C=O), 98.20 (C−1$_{Glu}$), 71.35, 70.97, 69.79, 68.99, 62.08, 54.49, 51.39, 51.29, 29.12, 28.76, 26.32, 25.37 (13 C), 20.80, 20.66, 20.57 (3 CO*C*H$_3$), 8.81 (2 CH_3); EI–MS (*m/z*, %) for $C_{24}H_{34}N_4O_{10}$ (538.22): 539.10 (M+1, 77%), 538.10 (M, 100%), 520.10 (77%), 490.10 (71%).

6-(4-{[(3β)-Cholest-5-en-3-yloxy]methyl}-1*H*-1,2,3-triazol-1-yl)hex-1-yl 3,4,6-tri-*O*-acetyl-2-deoxy-2-dimethylmaleimido-β-D-glucopyranoside (19)

Compounds **10** and **18** were clicked according to the general procedure and the residue was purified by flash chromatography (toluene:acetone, 4:1) to afford **19** (67%) as syrup. R_f 0.36 (toluene:acetone, 4:1); ^{1}H NMR (600 MHz, $CDCl_3$): δ 7.49 (s, 1H, H−5_{Triaz}), 5.58 (dd, 1H, $J_{2,3}$ 10.8, $J_{3,4}$ 9.6 Hz, H−3_{Glu}), 5.34 (m, 1H, H−6_{Chol}), 5.17 (d, 1H, $J_{1,2}$ 8.4 Hz, H−1_{Glu}), 5.09 (dd, 1H, $J_{3,4}$ 9.6, $J_{4,5}$ 9.0 Hz, H−4_{Glu}), 4.66 (s, 2H, OCH_2), 4.29−4.26 (m, 3H), 4.13 (dd, 1H, J_{gem} 12.0, $J_{5,6}$ 2.4 Hz, H−6), 3.80−3.76 (m, 2H), 3.42−3.48 (m, 1H), 3.33−3.29 (m, 1H), 2.41−2.38 (2m, 1H), 2.60−2.22 (m, 1H), 2.09−1.89 (m, 8H), 2.08, 2.00, 1.90 (3s, 9H, 3 CH_3CO), 1.93 (s, 6H, 2 $CH_{3Maleimid}$), 1.86−1.80 (m, 3H), 1.57−1.42 (m, 8H), 1.34−1.24 (m, 9H), 1.17−1.04 (m, 7H), 0.99 (s, 3H, CH_3−19_{Chol}), 0.90 (d, 3H, J 6.6 Hz, CH_3−21_{Chol}), 0.85, 0.84 (2d, 6H, J 3.0 Hz, CH_3−26_{Chol}, CH_3−27_{Chol}), 0.66 (s, 3H, CH_3−18_{Chol}); ^{13}C NMR (150 MHz, $CDCl_3$): δ 170.76, 170.23, 169.51 (5 C=O), 145.97, 140.70 125.31, 122.09, 121.85 (C−5_{Chol}, C−6_{Chol}, C−4_{Triaz}, C−5_{Triaz}, C=$C_{Maleimid}$), 98.20 (H−1_{Glu}), 78.98 (C−3_{Chol}), 71.77, 70.98, 69.70, 68.98, 62.06, 61.72, 56.77, 56.16, 54.50, 50.17, 42.34, 39.79, 39.53, 39.05, 37.19, 36.87, 36.20, 35.80, 31.96, 31.90, 30.21, 29.40, 29.05, 28.33, 28.25, 28.03, 26.14, 25.25 (28 C), 24.31 (CH_2−15_{Chol}), 23.84 (CH_2−23_{Chol}), 22.84, 22.58 (CH_3−26_{Chol}, CH_3−27_{CHol}), 21.08 (CH_2−11_{Chol}), 20.82, 20.67, 20.57 (3 CO*C*H_3), 19.39 (CH_3−19_{Chol}), 18.74 (CH_3−21_{Chol}), 11.88 (CH_3−18_{Chol}), 8.85 (2 $CH_{3Maleimide}$); $C_{54}H_{82}N_4O_{11}$ (963.25).

6-(4-{[(3β)-Cholest-5-en-3-yloxy]methyl}-1*H*-1,2,3-triazol-1-yl)hex-1-yl 2-deoxy-2-dimethylmaleimido-β-D-glucopyranoside (20)

Compound **19** (0.24 g, 0.0.25 mmol) was treated with NaOMe and worked up as described for **16** then purified by flash chromatography (toluene:acetone, 1:1) to afford **20** (0.15 g, 75%) as colorless foam. R_f 0.21 (toluene:acetone, 5:4); ^{1}H NMR (600 MHz, MeOH−d_4): δ 7.92 (s, 1H, H−5_{Triaz}), 5.35 (dd, 1H, J 2.4, 3.0 Hz, H−6_{Chol}), 4.94 (d, 1H, $J_{1,2}$ 8.4 Hz, H−1_{Glu}), 4.63 (s, 2H, OCH_2), 4.35 (dd, 2H, J 7.2 Hz, H−3_{Glu}, H−4_{Glu}), 4.07 (dd, 1H, $J_{1,2}$ 8.4, $J_{2,3}$ 10.8 Hz, H−2_{Glu}), 3.88 (dd, 1H, J_{gem} 12.0, $J_{5,6}$ 1.8 Hz, H−6_{Glu}), 3.82−3.79 (m, 1H), 3.72−3.68 (m, 2H), 3.41−3.37 (m, 1H), 3.34−3.32 (m, 1H), 3.31−3.26

(m, 1H), 2.39–2.38, 2.37–2.36 (2m, 1H), 2.22–2.17 (m, 1H), 2.06–2.02 (m, 1H), 1.98–1.78 (m, 7H), 1.93 (s, 6H, 2$CH_{3maleimid}$), 1.64–1.34 (m, 12 H), 1.32–0.90 (m, 15H), 1.01 (s, 3H, CH_3–19_{Chol}), 0.93 (d, 3H, *J* 6.0 Hz, CH_3–21_{Chol}), 0.88, 0.87 (2d, 6H, *J* 1.8 Hz, CH_3–26_{Chol}, CH_3–27_{Chol}), 0.71 (s, 3H, CH_3–18_{Chol}); ^{13}C NMR (150 MHz, MeOH–d_4): δ 174.28 (2C=O), 146.53 (C–4_{Triaz}), 141.83 (C–5_{Chol}), 138.40 (C=$C_{Maleimid}$), 124.90 (C–5_{Triaz}), 122.90 (C–6_{Chol}), 99.80 (C–1_{Glu}), 80.18, 78.25, 72.76, 72.59, 70.18, 62.76, 61.93, 58.39, 58.20, 57.60, 51.74, 51.24, 43.53, 41.18, 40.73, 40.12, 38.44, 37.98, 37.41, 37.17, 33.27, 33.09, 31.32, 30.18, 29.39, 29.36, 29.19, 27.04, 26.45, 25.35, 24.99, 23.23, 22.98, 22.22 (34 C), 19.87 (CH_3–19_{Chol}), 19.28 (CH_3–21_{Chol}), 12.36 (CH_3–18_{Chol}), 8.63 (2 $CH_{3Maleimid}$); EI–MS (*m/z*, %) for $C_{48}H_{76}N_4O_8$ (836.56): 831.10(9.2%), 830.10 (9.8%), 825.10 (9.3%), 687.110.5%), 642.10 (10.5%).

6-Azidohex-1-yl *O*-(2,3,4,6-tetra-*O*-acetyl-α-D-glucopyranosyl)-(1→4)-2,3,6-tri-*O*-acetyl-β-D-glucopyranoside (22)

A mixture of **21** (0.68 g, 0.85 mmol) and **9a** (0.1 g, 0.7 mmol) in dry DCM (5 mL) was stirred under N_2 at ambient temperature, while TMSOTf (0.01 M, 2 mol %) was added dropwise. The mixture was neutralized by Et_3N after 1h then evaporated in vacuo. The residue was purified by flash chromatography (toluene:ethyl acetate, 3:2) to afford **22** (0.1 g, 19%) as colorless syrup. R_f 0.32 (toluene:ethyl acetate, 3:2); IR (ύ, cm^{-1}): 2096 (N_{3str}), 1742 (C=O_{str}), 1212 (C–$O_{Pyran\ str}$), 1027 (CH_2–O_{str}); ^1H NMR (600 MHz, $CDCl_3$): δ 5.40 (d, 1H, $J_{1,2}$ 4.2 Hz, H–1b), 5.35 (t, 1H, *J* 9.6, 10.2 Hz), 5.24 (t, 1H, *J* 9.0 Hz), 5.04 (t, 1H, *J* 10.2, 9.6 Hz), 4.84 (dd, 1H, *J* 10.2, 4.2 Hz), 4.80 (dd, 1H, *J* 7.8, 9.0 Hz), 4.53–4.45 (m, 2H), 4.25–4.20 (m, 2H), 4.02 (dd, 1H, *J* 2.4, 12.6 Hz, H–6), 4.00 (t, 1H, *J* 9.6, 9.0 Hz), 3.96–3.93 (m, 1H), 3.67–3.65 (m, 1H), 3.48–3.44 (m, 1H), 3.36 (dd, 1H, *J* 6.6, 13.2 Hz), 3.26–3.23 (m, 2H), 2.13, 2.12, 2.09, 2.06, 2.03, 2.01, 2.00, 1.99 (7s, 21H, 7 $COCH_3$), 1.59–1.55 (m, 4H, CH_2–2_{Hex}, CH_2–5_{Hex}), 1.37–1.32 (m, 4H, CH_2–3_{Hex}, CH_2–4_{Hex}); ^{13}C NMR (150 MHz, $CDCl_3$): δ 170.52, 170.51, 170.46, 170.23, 169.94, 169.57, 169.40 (7 *C*OCH_3), 100.23 (C–1_a), 95.45 (C–1_b), 75.37, 72.67, 72.15, 72.03, 69.94, 69.85, 68.28, 69.41, 67.95, 62.79, 61.45, 51.28, 29.18, 28.71, 26.32, 25.35 (10

C_{Glu}, 6 CH_2–Hex), 20.88, 20.81, 20.65, 20.60, 20.57, 20.54, 20.53 (7 CO*C*H_3); $C_{32}H_{47}N_3O_{18}$ (761.72).

6-(4-{[(3β)-Cholest-5-en-3-yloxy]methyl}-1*H*-1,2,3-triazol-1-yl)hex-1-yl *O*-(2,3,4,6-tetra-*O*-acetyl-α-D-glucopyranosyl)-(1→4)-2,3,6-tri-*O*-acetyl-β-D-glucopyranoside (23)

A mixture of **22** (0.22 g, 0.3 mmol) and **10** (0.15 g, 0.35 mmol) was clicked and worked up according to the general procedure. The residue was purified by flash chromatography (toluene:acetone, 4:1) to afford **23** (0.21 g, 62%) as colorless oil. R_f 0.34 (toluene:acetone, 4:1); ^{1}H NMR (600 MHz, $CDCl_3$): δ 7.52 (s, 1H, H–5_{Triaz}), 5.40 (d, 1H, $J_{1,2}$ 4.2 Hz, H–1_b), 5.37–5.34 (m, 2H), 5.24 (t, 1H, *J* 9.6, 9.0 Hz), 5.05 (t, 1H, *J* 10.2, 9.6 Hz), 4.84 (dd, 1H, $J_{1,2}$ 4.2, $J_{2,3}$ 10.8 Hz, H–2_b), 4.80 (dd, 1H, *J* 7.8, 9.0 Hz), 4.67 (s, 2H, OCH_2), 4.49 (d, 1H, $J_{1,2}$ 7.8 Hz, H–1_a), 4.47 (dd, 1H, J_{gem} 12.0, $J_{5,6}$ 2.4 Hz, H–6_{Glu}), 4.31 (t, 2H, *J* 7.2 Hz), 4.24 (d, 1H, J_{gem} 12.6, $J_{5,6}$ 3.6 Hz, H–6_{Glu}), 4.21 (dd, 1H, J_{gem} 12.0, $J_{5,6`}$ 4.2 Hz, H–6\`$_{Glu}$), 4.06 (dd, 1H, J_{gem} 12.6, $J_{5,6`}$ 2.4 Hz, H–6\`$_{Glu}$), 3.99 (t, 1H, *J* 9.6, 9.0 Hz), 3.97–3.94 (2m, 1H), 3.84–3.81 (m, 1H), 3.68–3.65 (m, 1H), 3.47–3.41 (m, 1H), 3.36–3.30 (m, 1H, H–3_{Chol}), 2.41–2.39 (2m, 1H, H–4_{Chol}), 2.26–2.22 (m, 1H, H–7_{Chol}), 2.13, 2.10, 2.04, 2.02, 2.00, 1.99 (6s, 21H, 7 $COCH_3$), 2.07–1.84 (m, 6 H–Chol), 1.57–1.04 (m, 28H, 20 H–Chol, 4 CH_2–Hex), 0.98 (s, 3H, CH_3–19), 0.90 (d, 3H, $J_{20,21}$ 6.6 Hz, CH_3–21), 0.86, 0.85 (2d, 6H, $J_{25,26}$ = $J_{25,27}$ 2.4 Hz, CH_3–26, CH_3–27), 0.67 (s, 3H, CH_3–18); ^{13}C NMR (150 MHz, $CDCl_3$): δ 170.59, 170.51, 170.28, 170.01, 169.64, 169.46 (7 *C*OCH_3), 140.68 (C–4_{Triaz}, C–5_{Chol}), 121.85 (C–5_{Triaz}, C–4_{Chol}), 100.30 (C–1_a), 95.52 (C–1_b), 78.98, 75.41, 72.72, 72.20, 72.12, 70.01, 69.86, 69.35, 68.48, 68.01, 62.82, 61.70, 61.50, 56.77, 56.15, 50.16, 42.33, 39.78, 39.53, 39.04, 37.18, 36.86, 36.20, 35.80, 31.95, 31.89, 30.21, 29.72, 29.17, 28.32, 28.25, 28.03, 26.20, 25.32, 24.31, 23.83, 22.84, 22.58, 21.08 (39 C), 20.94, 20.90, 20.72, 20.70, 20.64, 20.61 (7 CO*C*H_3), 19.39 (CH_3–19), 18.73 (CH_3–21), 11.88 (CH_3–18); $C_{62}H_{95}N_3O_{19}$ (1186.43).

6-(4-{[(3β)-Cholest-5-en-3-yloxy]methyl}-1*H*-1,2,3-triazol-1-yl)hex-1-yl *O*-(α-D-glucopyranosyl)-(1→4)-β-D-glucopyranoside (24)

Compound **23** (0.17 g, 0.14 mmol) was treated with NaOMe/MeOH then worked up as described for the synthesis of **16**. The residue was purified by flash chromatography (ethyl acetate:iPrOH:H_2O, 6:2.5:1) to afford **24** (0.1 g, 78%) as amorphous colorless mass. R_f 0.37 (ethyl acetate:iPrOH:H_2O, 6:2.5:1); ^{1}H NMR (600 MHz, MeOH–d_4): δ 7.96 (s, 1H, H–5_{Triaz}), 5.36–5.35 (m, 1H, H–6_{Chol}), 5.14 (d, 1H, $J_{1,2}$ 3.6 Hz, H–1_b), 4.62 (s, 2H, OCH_2), 4.40 (t, 2H, J 7.8, 6.0 Hz), 4.25 (2d, 1H, J 7.8, 13.8 Hz), 3.88–3.86 (m, 2H), 3.82–3.78 (m, 3H), 3.68–3.58 (m, 4H), 3.54–3.50 (m, 2H), 3.43 (dd, 1H, J 9.6, 3.6 Hz), 3.36–3.33 (m, 1H, H–3_{Chol}), 3.25 (t, 1H, J 9.6, 9.0 Hz), 3.21 (dd, 1H, J 9.0, 7.8 Hz), 2.39–2.37 (m, 1H, H–4_{Chol}), 2.20 (t, 1H, J 12.6, 12.0 Hz, H–7_{Chol}), 2.05–1.82 (m, 6H), 1.61–1.05 (m, 28 H, 20 H–Chol, 4 CH_2–Hex), 1.01 (s, 3H, CH_3–19), 0.93 (d, 3H, $J_{20,21}$ 6.6 Hz, CH_3–21), 0.88, 0.87 (2d, 6H, $J_{25,26} = J_{25,27}$ 2.4 Hz, CH_3–26, CH_3–27), 0.71 (s, 3H, CH_3–18); ^{13}C NMR (150 MHz, MeOH–d_4): δ 141.82 (C–4_{Triaz}, C–5_{Chol}), 122.90 (C–5_{Triaz}, C–4_{Chol}), 104.33 (C–1_a), 104.08 (C–1_b), 81.39, 80.23, 77.88, 76.60, 75.10, 74.81, 74.73, 74.69, 74.19, 71.52, 70.63, 62.77, 62.19, 58.19, 57.60, 51.73, 43.53, 41.18, 40.72, 40.11, 38.43, 37.98, 37.41, 37.16, 33.27, 33.08, 31.25, 30.52, 29.39, 29.36, 29.19, 27.21, 26.45, 25.35, 24.98, 23.23, 22.98, 22.21, 19.87, 19.28, 15.48 (41 C), 12.35 (CH_3–18); $C_{48}H_{81}N_3O_{12}$ (892.17).

General procedure for propargylation of **25** *and* **29**.

A solution of the proper lactoside (1.0 mmol) and propargyl bromide (5.0 mmol) in dry Et_2O/DMF (1:1, 10 mL) was stirred in ice bath under dry atmosphere, while NaH (60%, 13.0 mmol) was added portionwise. The mixture was allowed to reach ambient temperature gradually and stirring was continued overnight then treated carefully with MeOH (10 mL) and coevaporated with toluene-H_2O azeotrope in vacuo. The residue was taken in ethyl acetate (50 mL), washed with H_2O (3 × 10 mL), dried over Na_2SO_4, evaporated in vacuo and the residue was purified by flash chromatography using toluene as eluent.

Benzyl *O*-[2,4,6-tri-*O*-benzyl-3-*O*-(prop-2-yn-1-yl)-β-D-galactopyranosyl]-(1→4)-2,3,6-tri-*O*-benzyl-β-D-glucopyranoside (26)

Yield: (quant.) as syrup; IR (ύ, cm^{-1}): 2286 (≡C–H_{str}), 2113 (C≡C_{str}), 1059 (C–O–C_{str}); ^{1}H NMR (600 MHz, $CDCl_3$): δ 7.38–7.22 (m, 35H, 7 Ph), 5.01 (d, 1H, *J* 10.8 Hz, C<u>H</u>$_2$Ph), 4.96 (d, 1H, *J* 11.4, C<u>H</u>$_2$Ph), 4.95 (d, 1H, *J* 12.0 Hz, C<u>H</u>$_2$Ph), 4.91 (d, 1H, *J* 10.8, C<u>H</u>$_2$Ph), 4.77–4.71 (m, 4H), 7.66 (d, 1H, *J* 12.0 Hz, C<u>H</u>$_2$Ph), 4.58 (d, 1H, *J* 12.6 Hz, C<u>H</u>$_2$Ph), 4.57 (d, 1H, *J* 11.4 Hz, C<u>H</u>$_2$Ph), 4.48 (d, 1H, $J_{1,2}$ 7.8 Hz, H–1_{Glu}), 4.42 (d, 1H, $J_{1,2}$ 7.8 Hz, H–1_{Gal}), 4.41 (d, 1H, *J* 12.0 Hz, C<u>H</u>$_2$Ph), 4.34 (d, 1H, *J* 12.0 Hz, C<u>H</u>$_2$Ph), 4.29–4.28 (m, 2H), 4.23 (d, 1H, *J* 12.0 Hz, C<u>H</u>$_2$Ph), 3.95 (dd, 1H, *J* 9.6 Hz), 3.92 (d, 1H, J_{gem} 3.0 Hz, ≡C–C*H*H), 3.80 (dd, 1H, J_{gem} 10.8, $J_{5,6}$ 4.2 Hz, H–6), 3.72 (dd, 1H, J_{gem} 10.2, $J_{5,6}$ <1 Hz, H–6), 3.68 (dd, 1H, $J_{1,2}$ 7.8, $J_{2,3}$ 9.6 Hz, H–2), 3.57–3.45 (m, 4H), 3.39–3.34 (m, 3H), 2.45 (t, 1H, *J* 1.8, 2.4 Hz, ≡C–H); ^{13}C NMR (150 MHz, $CDCl_3$): δ 139.15, 139.08, 138.80, 138.64, 138.38, 138.13, 137.60 (7 C_{ter}–Ph), 129.79, 129.04, 128.39, 128.33, 128.29, 128.25, 128.17, 128.13, 128.09, 128.00, 127.90, 127.84, 127.82, 127.80, 127.74, 127.71, 127.70, 127.55, 127.48, 127.36, 127.11 (35 C_{sec}–Ph), 102.77 (C–1_{Glu}), 102.54 (C–1_{Gal}), 83.00, 82.00, 81.80, 80.45, 80.02, 75.41, 75.19, 75.17, 75.08, 74.75, 74.38, 74.19, 73.41, 73.19, 73.02, 71.00, 68.25, 68.10 (19C), 58.43 (≡C–*C*H_2); $C_{64}H_{66}O_{11}$ (1011.3).

Benzyl *O*-[2,3,6-tri-*O*-benzyl-4-*O*-(prop-2-yn-1-yl)-β-D-galactopyranosyl]-(1→4)-2,3,6-tri-*O*-benzyl-β-D-glucopyranoside (30)

Yield: (quant.) as syrup; R_f 0.11 (toluene); IR (ύ, cm^{-1}): 2292 (≡C–H_{str}), 2114 (C≡C_{str}), 1059 (C–O–C_{str}); ^{1}H NMR (600 MHz, $CDCl_3$): δ 7.38–7.22 (m, 35H, 7 Ph), 5.00 (d, 1H, *J* 10.2 Hz, C<u>H</u>$_2$Ph), 4.95 (d, 1H, *J* 12.6, C<u>H</u>$_2$Ph), 4.91 (d, 1H, *J* 10.8 Hz, C<u>H</u>$_2$Ph), 4.98–4.74 (m, 5H), 4.68 (d, 1H, *J* 12.0 Hz, C<u>H</u>$_2$Ph), 4.66 (d, 1H, *J* 12.0 Hz, C<u>H</u>$_2$Ph), 4.56 (dd, 1H, J_{gem} 12.0, $J_{5,6}$ 1.8 Hz, H–6), 4.50–4.36 (m, 7H), 4.04 (s, 1H, ≡C–C*H*H), 3.96 (dd, 1H, *J* 9.0, 7.8 Hz), 3.81 (d, 1H, *J* 10.2Hz), 3.74 (d, 1H, *J* 10.8 Hz), 3.68 (dd, 1H, *J* 9.0, 7.8 Hz), 3.63 (dd, 1H, *J* 8.4, 7.8 Hz), 3.56 (dd, 1H, *J* 9.0 Hz), 3.47 (m, 1H), 3.43–3.36 (m, 4H), 2.39 (d, 1H, *J* 1.8, 2.4 Hz, ≡C–H); ^{13}C NMR (150 MHz, $CDCl_3$): δ 139.10, 138.79, 138.68, 138.41, 138.27, 137.60, 134.50 (7 C_{ter}–Ph), 129.79, 129.04,

128.48, 128.42, 128.39, 128.30, 128.27, 128.11, 128.08, 127.91, 127.83, 127.80, 127.71, 127.668, 127.64, 127.58, 126.56, 127.48, 127.27 (35 C_{sec}–Ph), 102.61 ($C{-}1_{Glu}$), 102.55 ($C{-}1_{Gal}$), 83.00, 82.40, 81.90, 80.52, 80.08, 76.52, 75.45, 75.31, 75.22, 75.07, 74.46, 73.48, 73.14, 73.01, 72.90, 72.33, 71.00, 68.35 (19C), 59.49 (≡C–CH_2); $C_{64}H_{66}O_{11}$ (1011.3).

Benzyl *O*-[2,6-di-*O*-benzyl-3-*O*-(prop-2-yn-1-yl)-β-D-galactopyranosyl]-(1→4)-2,3,6-tri-*O*-benzyl-β-D-glucopyranoside (34)

A mixture of diol **33** (0.6 g, 0.7 mmol) and Bu_2SnO (0.2 g, 0.8 mmol) in dry MeOH (10 mL) was gently refluxed then evaporated in vacuo. The residue was refluxed, in toluene (10 mL), with TBAI (0.27 g, 0.7 mmol) and propargyl bromide (80 % w/w in toluene, 1.7 mmol). The mixture was evaporated in vacuo after 2 h and the residue was purified by flash chromatography using solvent gradient of petroleum ether, toluene then (toluene:acetone, 5:1) to afford **34** (0.57 g, 92%) as syrup; R_f 0.29 (toluene:acetone, 5:1); $C_{57}H_{60}O_{11}$ (921.17).

Benzyl *O*-(2,4,6-tri-*O*-benzyl-3-*O*-({1-[(3β)-cholest-5-en-3-yl]-1*H*-1,2,3-triazol-4-yl}methyl)-β-D-galactopyranosyl)-(1→4)-2,3,6-tri-*O*-benzyl-β-D-glucopyranoside (27)

A mixture of **3** (0.2 g, 0.5 mmol) and **26** (0.42 g, 0.41 mmol) was clicked and worked up according to the general procedure. The residue was purified by flash chromatography (toluene:ethyl acetate, 5:1) to afford **27** (0.53 g, 89%) as syrup. R_f 0.27 (toluene:ethyl acetate, 5:1); ^{1}H NMR (600 MHz, $CDCl_3$): δ 7.71(s, 1H, $H{-}5_{Triaz}$), 7.42–7.11 (m, 35, 7 Ph), 5.28 (m, 1H, $H{-}6_{Chol}$), 5.00 (d, 1H, J_{gem} 10.2 Hz, C$\underline{H}_2$Ph), 4.94 (d, 1H, J_{gem} 12.0 Hz, C$\underline{H}_2$Ph), 4.91 (d, 1H, J_{gem} 12.0 Hz, C$\underline{H}_2$Ph), 4.90 (d, 1H, J_{gem} 10.8 Hz, C$\underline{H}_2$Ph), 4.86–4.84 (m, 3H, OCH*H*, C$\underline{H}_2$Ph), 4.79 (d, 1H, J_{gem} 11.4 Hz, C$\underline{H}_2$Ph), 4.74 (d, 1H, J_{gem} 10.8 Hz, C$\underline{H}_2$Ph), 4.72 (d, 1H, J_{gem} 11.4 Hz, C$\underline{H}_2$Ph), 4.70 (d, 1H, J_{gem} 10.8 Hz, C$\underline{H}_2$Ph), 4.65 (d, 1H, J_{gem} 12.0 Hz, C$\underline{H}_2$Ph), 4.54 (d, 1H, J_{gem} 12.6 Hz, C$\underline{H}_2$Ph), 4.51 (d, 1H, J_{gem} 11.4 Hz, C$\underline{H}_2$Ph), 4.47 (d, 1H, $J_{1,2}$ 7.8 Hz, $H{-}1_{Glu}$), 4.43 (d, 1H, $J_{1,2}$ 8.4 Hz, $H{-}1_{Gal}$), 4.41 (d, 1H, J_{gem} 12.6 Hz, C$\underline{H}_2$Ph), 4.31 (d, 1H, J_{gem} 12.0 Hz, C$\underline{H}_2$Ph), 4.20 (d, 1H, J_{gem} 11.4

Hz, C$\underline{H}_2$Ph), 3.93 (t, 1H, *J* 9, 9.6 Hz), 3.86 (d, 1H, *J* 2.4 Hz, H–4_{Gal}), 3.77 (dd, 1H, J_{gem} 11.4, *J* 5,6 4.8 Hz, H–$6_{Sug.}$), 3.72–3.69 (m, 2H), 3.54–3.45 (m, 4H), 3.38–3.31 (m, 3H), 2.89 (d, 1H, *J* 15.6 Hz), 2.37–2.35 (m, 2H), 2.18 (d, 1H, *J* 13.8 Hz), 2.13–2.05 (m, 1H), 1.92–1.87 (m, 2H), 1.79–1.74 (m, 2H), 1.62 (d, 1H, *J* 13.8Hz), 1.56–1.52 (m, 1H), 1.44–1.33 (m, 8H), 1.23–1.12 (m, 4H), 1.05 (s, 3H, CH_3–19), 1.02–0.94 (m, 5H), 0.89, 0.88 (2d, s, 9H, $J_{25,26}$ = $J_{25,27}$ = 2.4 Hz, CH_3–26, CH_3–27, CH_3–21), 0.63 (s, 3H, CH_3–18); ^{13}C NMR (150 MHz, $CDCl_3$): δ 139.16, 138.99, 138.85, 138.65, 138.41, 138.18, 138.02, 137.61, 129.07, 128.43, 128.39, 128.36, 128.33, 128.29, 128.27, 128.26, 128.19, 128.14, 128.09, 128.00, 127.94, 127.91, 127.78, 127.77, 127.70, 127.65, 127.62, 127.55, 127.52, 127.47, 127.46, 127.15, 127.10, 125.33, 124.22, 121.92, (42 C–Ph, C–5_{Chol}, C–6_{Chol}, C–4_{Triaz}, C–5_{Triaz}), 102.71 (C–1_{Glu}), 102.50 (C–1_{Gal}), 83.02, 82.72, 81.81, 79.82, 75.38, 75.18, 75.09, 75.08, 74.79, 73.70, 73.33, 73.14, 72.97, 70.96, 68.28, 68.08, 64.84, 56.59, 56.31, 55.95, 50.02, 42.16, 39.55, 39.41, 37.04, 36.20, 35.77, 35.36, 32.75, 31.82, 31.55, 28.15, 28.06, 27.11, 24.13, 23.89, 22.87, 22.61, 20.59, 19.23, 18.70 (42 C), 11.81 (CH_3–18); $C_{91}H_{111}N_3O_{11}$ (1423.05).

Benzyl *O*-(2,3,6-tri-*O*-benzyl-4-*O*-({1-[(3β)-cholest-5-en-3-yl]-1*H*-1,2,3-triazol-4-yl}methyl)-β-D-galactopyranosyl)-(1→4)-2,3,6-tri-*O*-benzyl-β-D-glucopyranoside (31)

A mixture of **3** (0.2 g, 0.5 mmol) and **30** (0.5 g, 0.5 mmol) was clicked and worked up according to the general procedure. The residue was purified by flash chromatography (toluene:ethyl acetate, 9:1) to afford **31** (0.51 g, 74%) as syrup. R_f 0.17 (toluene:ethyl acetate, 9:1); ^{1}H NMR (600 MHz, $CDCl_3$): δ 7.75 (s, 1H, H–5_{Triaz}), 7.38–7.24 (m, 35H, 7 Ph), 5.34 (br.t, 1H, *J* 2.4, 4.8 Hz, H–5_{Chol}), 5.30 (s, 1H), 5.01 (d, 1H, J_{gem} 12.0 Hz, 0.5 C$\underline{H}_2$Ph), 4.96 (d, 1H, J_{gem} 10.2 Hz, 0.5 C$\underline{H}_2$Ph), 4.95 (d, 1H, J_{gem} 12.0 Hz, 0.5 C$\underline{H}_2$Ph), 4.90 (d, 1H, J_{gem} 11.4 Hz, 0.5 C$\underline{H}_2$Ph), 4.86 (d, 1H, J_{gem} 12.0 Hz, 0.5 C$\underline{H}_2$Ph), 4.80 (d, 1H, J_{gem} 10.8 Hz, 0.5 C$\underline{H}_2$Ph), 4.76 (d, 1H, J_{gem} 10.2 Hz, 0.5 C$\underline{H}_2$Ph), 4.73–4.68 (m, 10H, OCH_2, 2.5 C$\underline{H}_2$Ph), 4.65 (d, 1H, J_{gem} 11.4 Hz, 0.5 C$\underline{H}_2$Ph), 4.50 (d, 1H, $J_{1,2}$ 7.8 Hz, H–1_a), 4.45 (d, 1H, $J_{1,2}$ 7.8 Hz, H–1_b), 4.40 (d, 1H, J_{gem} 12.0 Hz, 0.5 C$\underline{H}_2$Ph), 4.36 (s, 1H), 4.02 (dd, 1H, $J_{3,4}$ 3.0, $J_{4,5}$ < 1.0 Hz, H–4_b), 3.96 (dd, 1H, *J* 9.6, 9.0 Hz, $H_{Sug.}$), 3.79

(dd, 1H, J_{gem} 10.8, $J_{5,6}$ 4.2 Hz, H–6$_{Sug.}$), 3.74 (dd, 1H, J_{gem} 10.8, $J_{5,6}$ 1.2 Hz, H–6$_{Sug.}$), 3.70 (dd, 1H, $J_{1,2}$ 7.8, $J_{2,3}$ 9.6 Hz, H–2$_{Sug.}$), 3.58–3.54 (m, 2H), 3.48–3.46 (m, 1H), 3.42–3.35 (m, 4H), 2.80 (br.d, 1H, J 14.4 Hz), 2.29 (d, 1H, J 15.6 Hz), 2.02–1.89 (m, 5H), 1.72–1.69 (m, 2H), 1.56–1.39 (m, 4H), 1.37–1.25 (m, 7H), 1.20–1.06 (m, 3H), 1.05–0.93 (m, 4H), 0.87, 0.86 (2d, 6H, $J_{25,26}$ = $J_{25,27}$ 2.4 Hz, CH_3–26, CH_3–27), (d, 3H, $J_{20,21}$ 6.6 Hz, CH_3–21), 0.63 (s, 3H, CH_3–18); ^{13}C NMR (150 MHz, $CDCl_3$): δ 144.86, 140.92, 138.85, 138.71, 138.43, 138.38, 138.19, 138.06, 137.60, 136.43, 134.50, 133.43, 130.14, 129.79, 129.03, 128.59, 128.44, 128.42, 128.41, 128.39, 128.38, 128.31, 128.29, 128.27, 128.08, 128.06, 127.87, 127.86, 127.79, 127.70, 127.68, 127.66, 127.61, 127.56, 127.52, 127.48, 127.33, 127.02, 124.10, 122.39 (42 C–Ar, C–4$_{Triaz}$, C–5$_{Chol}$, C–5$_{Triaz}$, C–6$_{Chol}$), 102.65 (C–1$_a$), 102.60 (C–1$_b$), 82.97, 82.26, 81.86, 80.33, 76.45, 75.57, 75.54, 75.27, 75.01, 73.61, 73.49, 73.12, 72.99, 72.46, 71.04, 68.36, 68.21, 66.71, 65.39, 56.45, 56.41, 56.16, 49.94, 42.21, 39.50, 36.98, 36.16, 35.80, 35.32, 32.67, 31.93, 31.61, 28.17, 28.05, 26.97, 24.18, 24.05, 22.86, 22.60, 20.57, 19.21, 18.69 (42 C), 11.80 (CH_3–18); $C_{91}H_{111}N_3O_{11}$ (1423.05).

Benzyl *O*-(2,6-di-*O*-benzyl-3-*O*-({1-[6-chloro-2-methylquinolin-4-yl]-1*H*-1,2,3-triazol-4-yl}methyl)-β-D-galactopyranosyl)-(1→4)-2,3,6-tri-*O*-benzyl-*β*-D-glucopyranoside (36)

A mixture of **34** (0.1 g, 0.45 mmol) and **35** (0.3 g, 0.32 mmol) was clicked and worked up according to the general procedure. The residue was purified by flash chromatography (toluene:ethyl acetate, 4:10) to afford **36** (0.28 g, 76%) as syrup. R_f 0.2 (toluene:ethyl acetate, 4:1); ^{1}H NMR (600 MHz, DMSO–d_6): δ 8.08 (d, 1H, J 9.0 Hz, Ar), 7.85 (t, 1H, J 3.6, 2.4 Hz, Ar), 7.80 (s, 1H, H–5$_{Triaz}$), 7.72 (dd, 1H, J 2.4, 9.0 Hz, Ar), 7.39–7.36 9 (m, 4H, Ar), 7.33–7.22 (24H, Ar), 7.15–7.13 (m, 2H, Ar), 7.08 (t, 1H, J 7.2, 7.8 Hz), 5.02–4.90 (m, 5H), 4.79, 4.71 (2d, 2H, J_{gem} 11.4 Hz, C$\underline{H}_2$Ph), 4.77, 4.72 (2d, 2H, J_{gem} 10.8 Hz, C$\underline{H}_2$Ph), 4.65, 4.59 (2d, 2H, J_{gem} 12.0 Hz, C$\underline{H}_2$Ph), 4.51–4.47 (m, 3H), 4.45–4.40 (m, 3H), 4.15 (d, 1H, J 3.0 Hz), 4.02 (t, 1 H, J 9.6 Hz), 3.83 (dd, 1H, $J_{5,6}$ 4.8, J_{gem} 11.4 Hz, H–6), 3.75 (dd, 1H, $J_{5,6}$ 1.8, J_{gem} 10.8 Hz, H–6), 3.68 (dd, 1H, J 7.2, 9.6 Hz), 3.63 (dd, 1H, J 7.8, 9.6 Hz), 3.58 (t, 1H, J 9.0 Hz), 3.53–3.46 (m, 3H), 3.39–3.38

(m, 2H), 2.79 (s, 3H, CH_3); ^{13}C NMR (150 MHz, $MeOH_{d4}$): δ 159.54, 147.80, 146.15, 140.09, 139.11, 138.59, 138.55, 138.31, 138.01, 137.52, 133.65, 131.72, 130.86, 129.06, 128.43, 128.40, 128.35, 128.29, 128.19, 128.12, 128.11, 128.00, 127.91, 127.74, 127.71, 127.69, 127.64, 127.58, 127.54, 127.47, 127.29, 124.25, 121.76, 121.23, 117.69 (47 C–Ar), 102.57 ($C{-}1_{Glu}$), 102.52 ($C{-}1_{Gal}$), 82.92, 81.85, 81.72, 79.49, 76.57, 75.34, 75.25, 75.18, 75.02, 73.56, 73.25, 72.68, 71.01, 68.57, 68.32, 66.31, 63.47 (17 C), 25.34 (CH_3); $C_{67}H_{67}ClN_4O_{11}$ (1139.83).

Benzyl *O*-(4-*O*-acetyl-2,6-di-*O*-benzyl-3-*O*-({1-[11-acetoxyundec-1-yl]-1*H*-1,2,3-triazol-4-yl}methyl)-β-D-galactopyranosyl)-(1→4)-2,3,6-tri-*O*-benzyl-β-D-glucopyranoside (39)

A mixture of **34** (0.16 g, 0.75 mmol) and **9b** (0.5 g, 0.54 mmol) was clicked and worked up according to the general procedure. The residue was purified by flash chromatography (toluene:acetone, 3:1) to afford **38** (0.44 g, 71%) as syrup; R_f 0.41 (toluene:acetone, 5:3). Compound **38** (15.0 mg, 13.0 μmol) was stirred with Ac_2O/pyridine (2:1, 1 mL) at ambient temperature overnight then coevaporated with toluene–H_2O azeotrope in vacuo. The residue was purified by flash chromatography (toluene:acetone, 7:1) to afford **39** (14.0 mg, 90%) as colorless mass. R_f 0.71 (toluene:acetone, 5:2); ^{1}H NMR (600 MHz, $CDCl_3$): δ 7.42–7.16 (m, 31H, 6 Ph, $H{-}5_{Triaz}$), 5.46 (d, 1H, $J_{3,4}$ = $J_{4,5}$ <1.0 Hz, H–4b), 4.95 (d, 1H, *J* 10.2 Hz, C<u>H</u>$_2$Ph), 4.94 (d, 1H, *J* 12.6 Hz, C<u>H</u>$_2$Ph), 4.89 (d, 1H, J_{gem} 10.8 Hz, C<u>H</u>$_2$Ph), 4.80 (d, 1H, *J* 12.6 Hz, C<u>H</u>$_2$Ph), 4.74–4.68 (m, 6H), 4.65 (d, 1H, J_{gem} 12.0 Hz, C<u>H</u>$_2$Ph), 4.57 (d, 1H, J_{gem} 12.0 Hz, C<u>H</u>$_2$Ph), 4.49–4.44 (m, 3H), 4.39 (d, 1H, J_{gem} 12.0 Hz, C<u>H</u>$_2$Ph), 4.23 (d, 1H, *J* 12.0 Hz, C<u>H</u>$_2$Ph), 4.21 (dd, 1H, *J* 7.2 Hz), 4.16 (dd, 1H, *J* 7.2 Hz), 4.05 (m, 2H, *J* 6.6, 7.2 Hz), 3.97 (t, 1H, *J* 9.6, 9.0 Hz), 3.79 (dd, 1H, J_{gem} 10.8, *J* 5,6 4.2 Hz, H–6), 3.72 (dd, 1H, J_{gem} 10.8, $J_{5,6}$ 1.8 Hz, H–6), 3.54 (t, 1H, *J* 9.0 Hz), 3.49–3.42 (m, 4H), 3.36–3.34 (m, 1H), 3.31–3.30 (m, 1H), 2.04, 2.03 (2s, 6H, $2COCH_3$), 1.78 (t, 2H, *J* 6.6, 7.2 Hz, CH_2), 1.61 (t, 2H, *J* 7.2, 7.8 Hz, CH_2), 1.32–1.17 (m, 14H, 7 CH_2); ^{13}C NMR (150 MHz, $CDCl_3$): δ 171.29, 170.26, (2*C*OCH_3), 145.14 ($C{-}4_{Triaz}$), 139.05, 138.63, 138.56, 138.21, 137.86, 137.50, 128.39, 128.38, 128.34, 128.28, 128.26, 128.09, 127.98, 127.92, 127.88, 127.71, 127.70, 127.68, 127.61, 127.57, 127.49, 127.31,

122.68 (36 C–Ar, C–5_{Triaz}), 102.49 (C–1_a), 102.32 (C–1_b), 82.83, 81.75, 79.54, 79.37, 76.46, 75.26, 75.14, 75.06, 75.03, 73.39, 73.20, 71.54, 70.98, 68.15, 67.08, 66.87, 64.64, 63.92, 50.24 (10 C–sugar, 6 CH_2Ph, 3 CH_2), 30.26, 29.45, 29.44, 29.37, 29.22, 28.99, 28.58, 26.41, 25.89, 21.04, 20.92 (2 COCH_3, 9 CH_2), $C_{72}H_{87}N_3O_{14}$ (1218.62).

Benzyl *O*-(3-*O*-({1-[11-hydroxyundec-1-yl]-1*H*-1,2,3-triazol-4-yl}methyl)-β-D-galactopyranosyl)-(1→4)-β-D-glucopyranoside (40)

Compound **38** (0.34 g, 0.3 mmol) was stirred with Pd/C–10% (50.0 mg) in deaereated MeOH/AcOH (5:1, 6 mL) under H_2 overnight. The mixture was filtered through celite, washed with MeOH, evaporated in vacuo and the residue was purified by flash chromatography (ethyl acetate:iPrOH:H_2O, 5:1.5:0.5) to afford **40** (0.11 g, 62%) as colorless amorphous mass. R_f 0.28 (ethyl acetate:iPrOH:H_2O, 5:1.5:0.5); ^{1}H NMR (600 MHz, MeOH–d_4): δ 8.05 (s, 1H, H–5_{Triaz}), 5.11 (d, 0.55H, $J_{1,2}$ 3.6 Hz, H–1_α), 4.84 (dd, 1H, J_{gem} 12.6 , *J* 5,6` < 1 Hz, H–6`), 4.76 (dd, 1H, J_{gem} 12.6, *J* 5,6` 2.4 Hz, H–6`), 4.50 (d, 0.45H, $J_{1,2}$ 7.8 Hz, 1–H_β), 4.41 (t, 2H, *J* 7.8, 8.4 Hz, C$\underline{H}_2$OH), 4.06 (m, 1H), 3.89–3.86 (m, 2H), 3.82–3.78 (m, 3H), 3.74–3.70 (m, 2H), 3.69–3.65 (m, 1H), 3.59–3.58 (m, 1H), 3.56 (dd, 1H, *J* 7.8, < 1.0 Hz), 3.52 (t, 2H, *J* 6.6 Hz, N–CH_2), 3.45–3.43 (m, 2H), 1.90 (q, 2H, *J* 7.2 Hz, –C$\underline{H}_2$$CH_2$N), 1.50 (q, 2H, –C$\underline{H}_2$$CH_2$OH), 1.32–1.29 (m, 14H, 7 CH_2); ^{13}C NMR (150 MHz, MeOH–d_4): δ 145.96 (C–5_{Triaz}), 125.25 (C–4_{Triaz}), 105.01 (C–1_b), 98.08 (C–$1_{a\beta}$), 93.67 (C–$1_{a\alpha}$), 82.81, 81.20, 76.90, 76.54, 76.47, 75.95, 73.55, 73.27, 71.75, 66.99, 66.95, 63.72, 63.04, 62.62, 62.59, 62.14, 62.05, 51.69, 33.68, 31.27, 30.73, 30.62, 30.61, 30.57, 30.37, 30.12, 27.48, 26.97 (22 C). $C_{26}H_{47}N_3O_{12}$ (593.76).

NMR spectral data

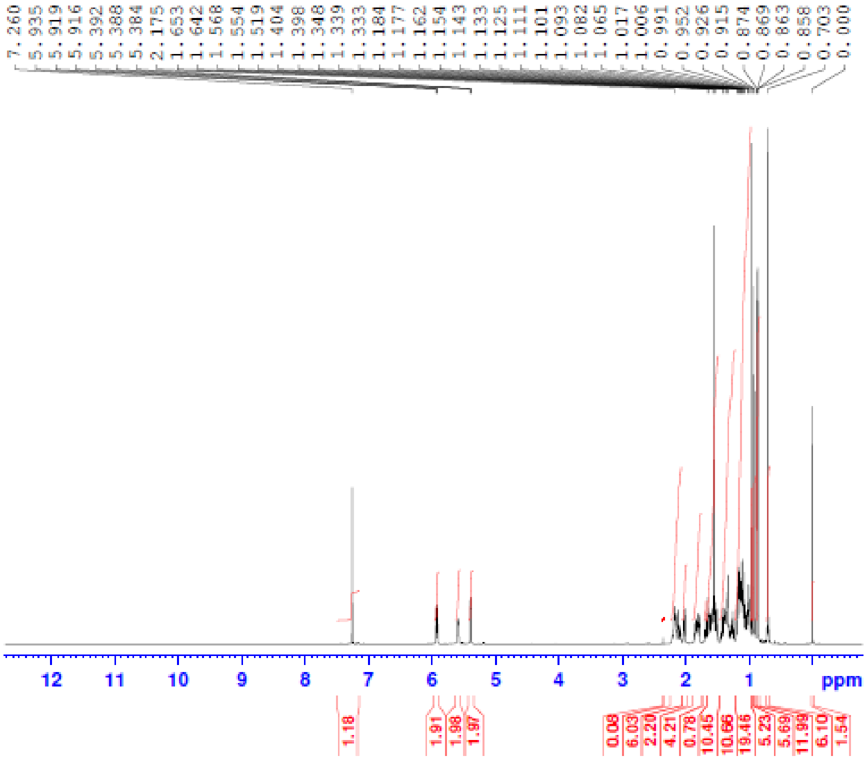

Fig. S1. ^{1}H NMR spectrum of compound **2**.

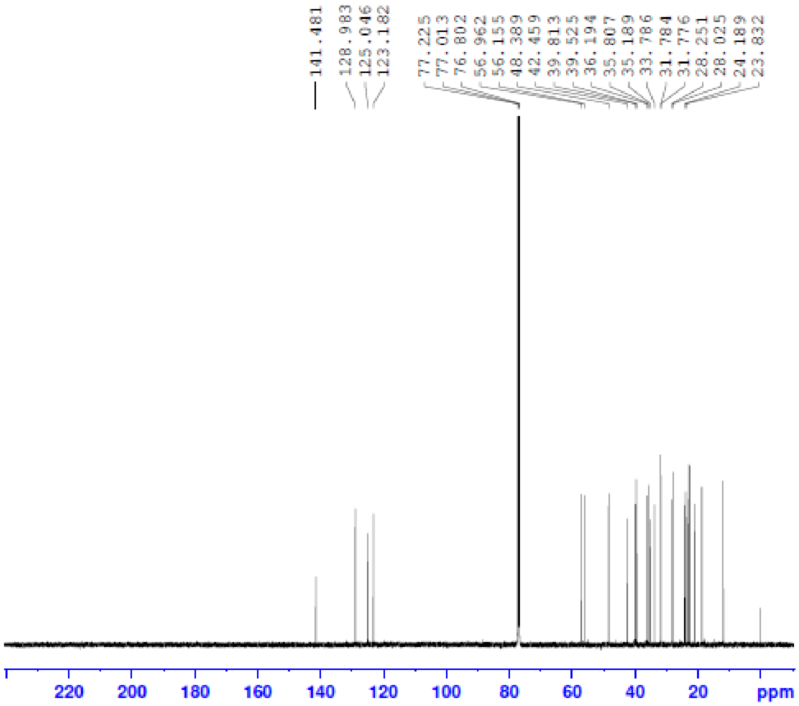

Fig. S2. ^{13}C NMR spectrum of compound **2**.

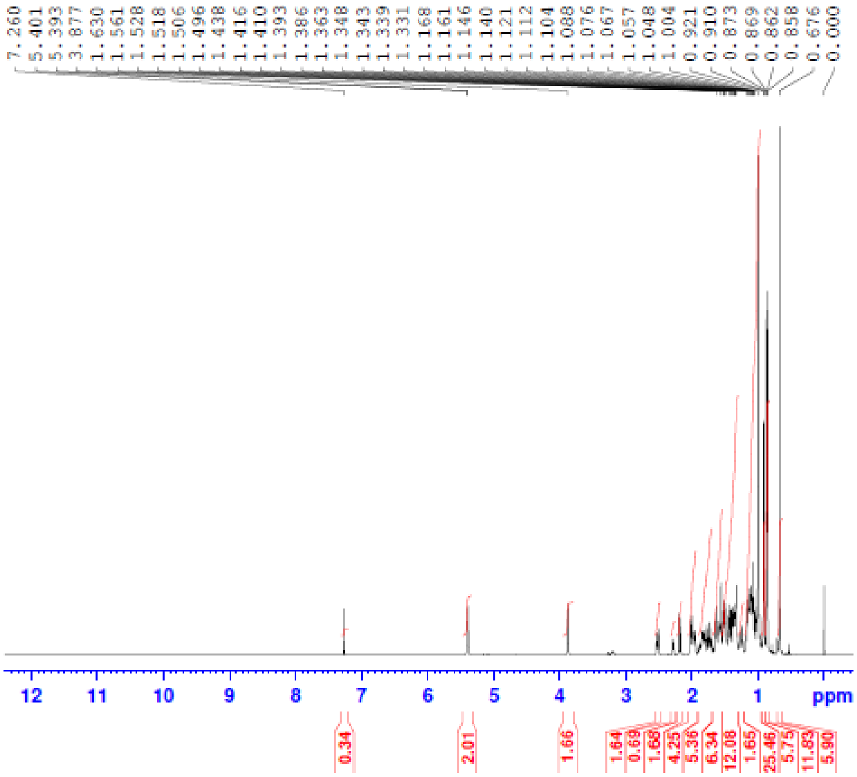

Fig. S3. ^{1}H NMR spectrum of compound **3**.

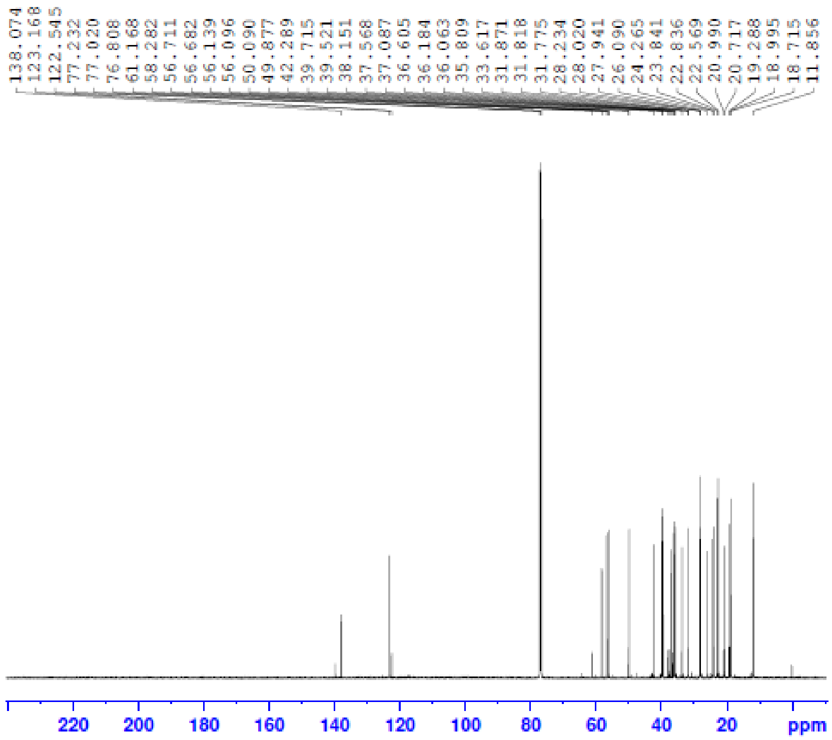

Fig. S4. ^{13}C NMR spectrum of compound **3**.

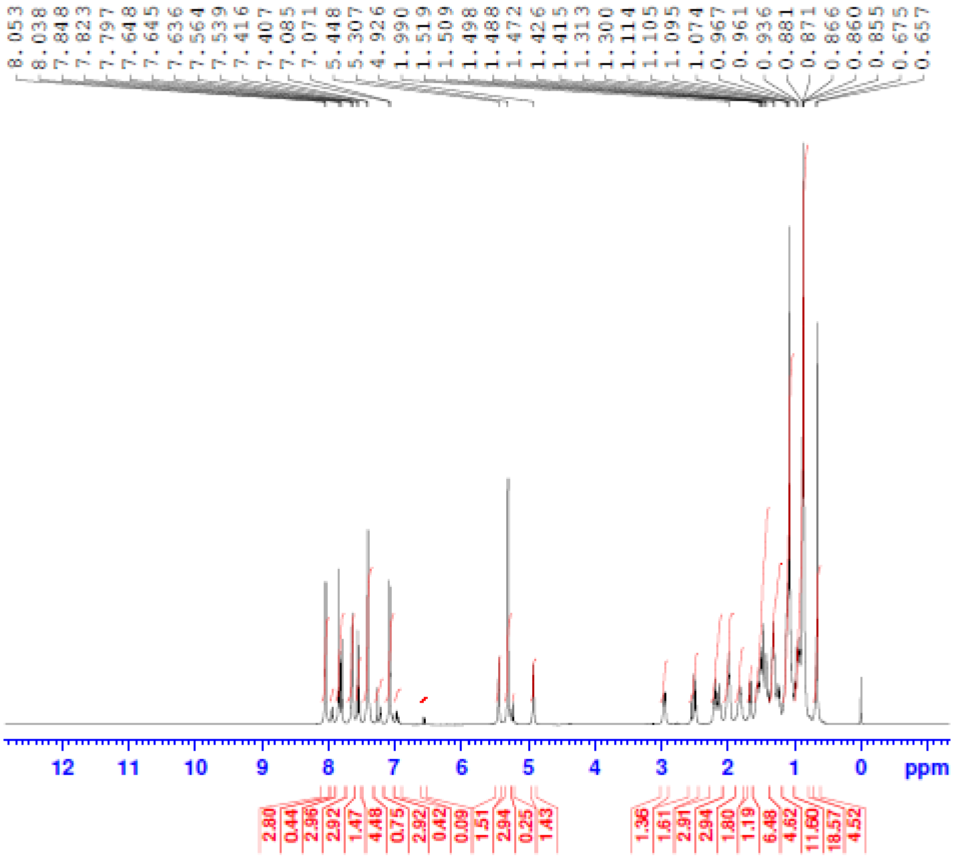

Fig. S5. ^{1}H NMR spectrum of compound **6a**.

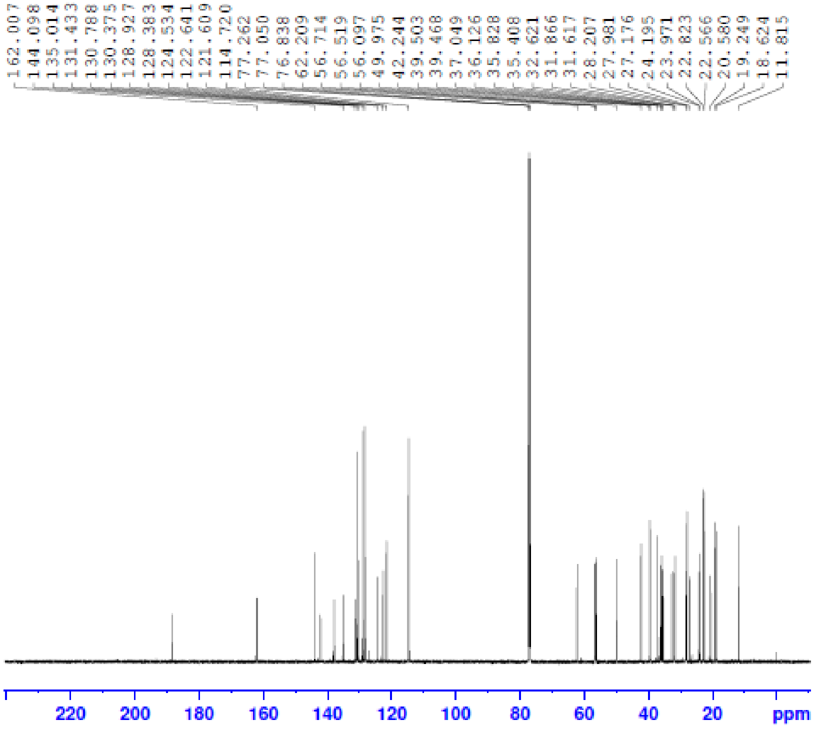

Fig. S6. ^{13}C NMR spectrum of compound **6a**.

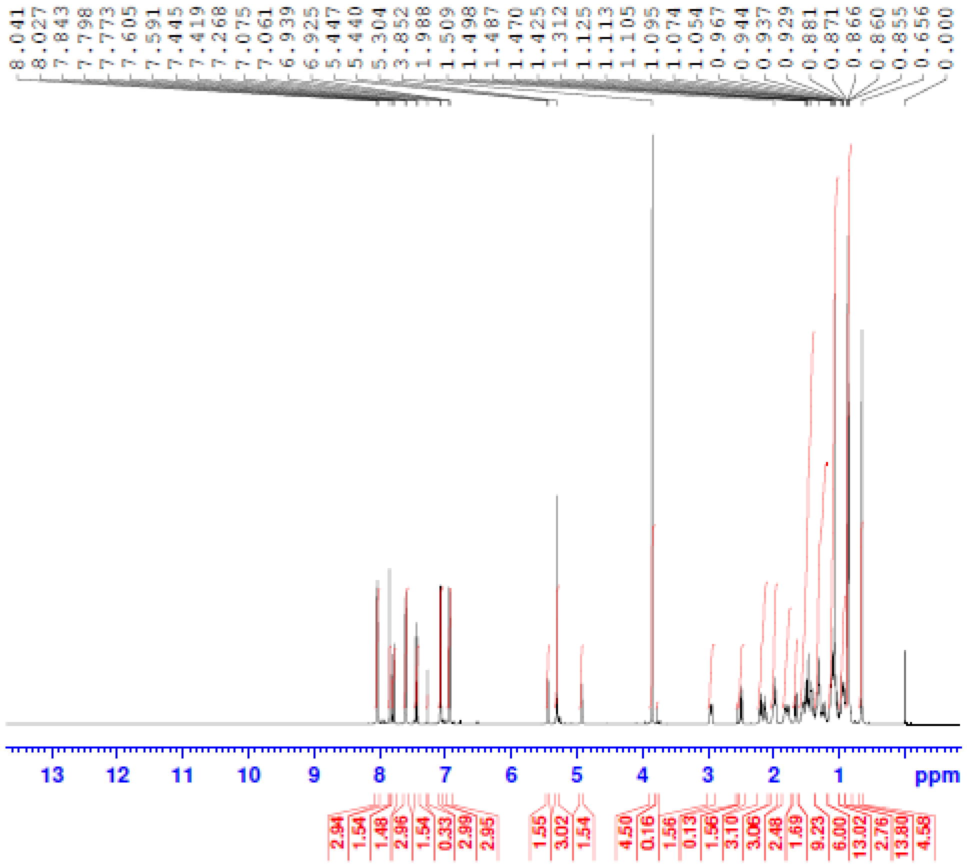

Fig. S7. ^{1}H NMR spectrum of compound **6b**.

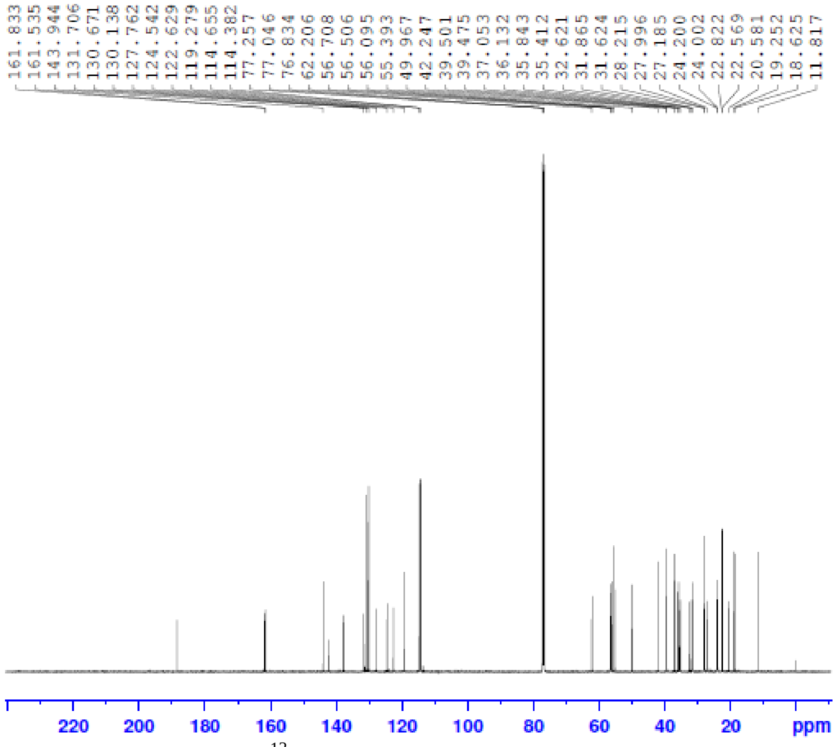

Fig. S8. ^{13}C NMR spectrum of compound **6b**.

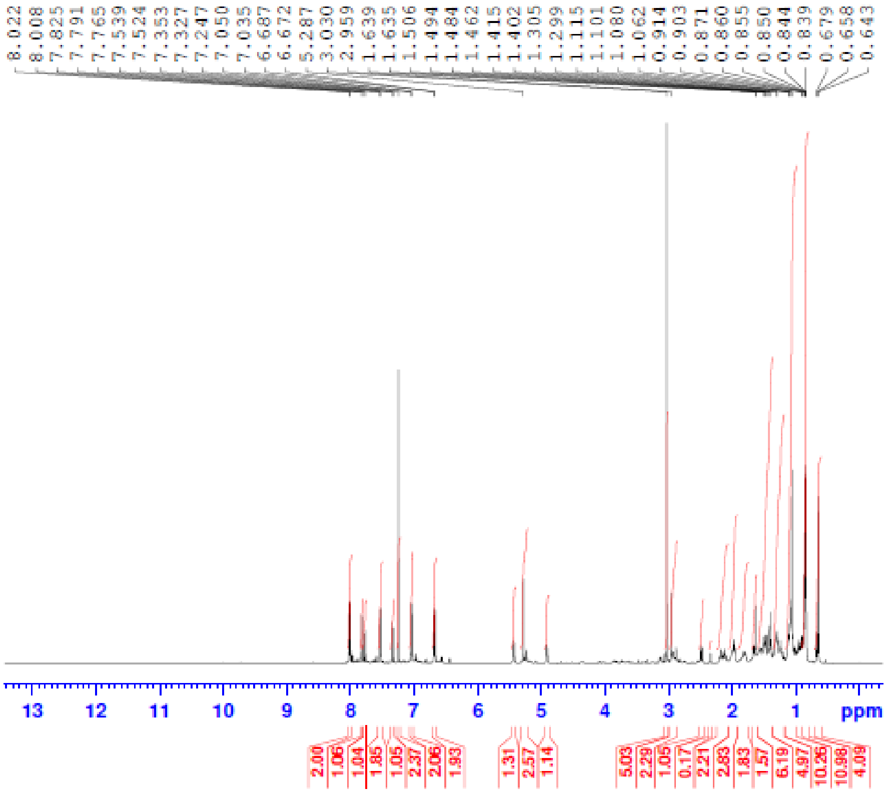

Fig. S9. ^{1}H NMR spectrum of compound **6c**.

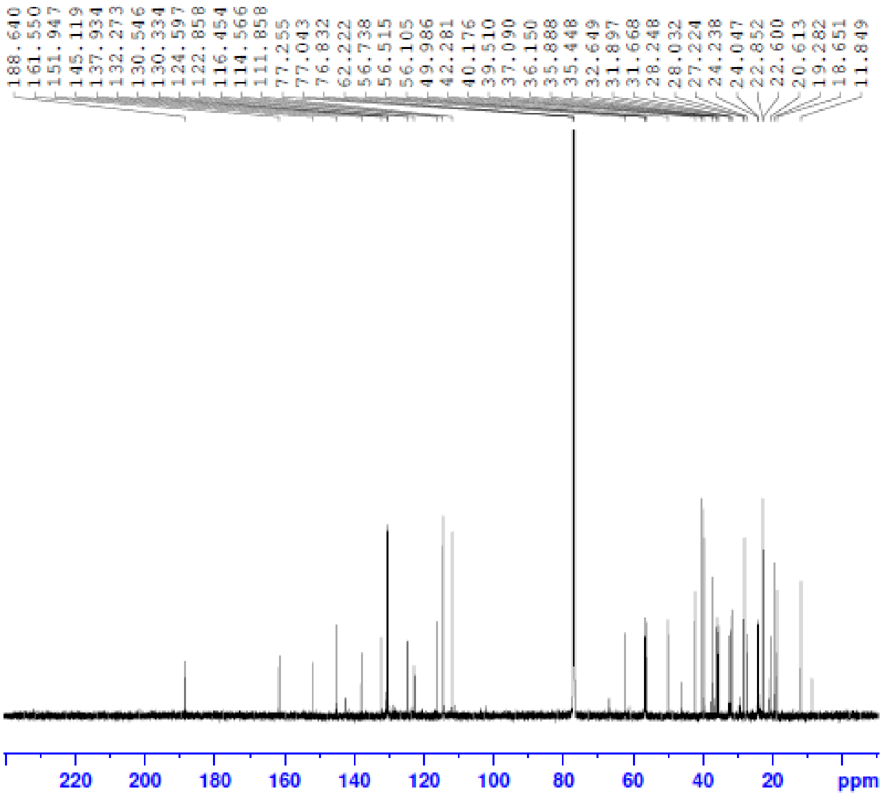

Fig. S10. ^{13}C NMR spectrum of compound **6c**.

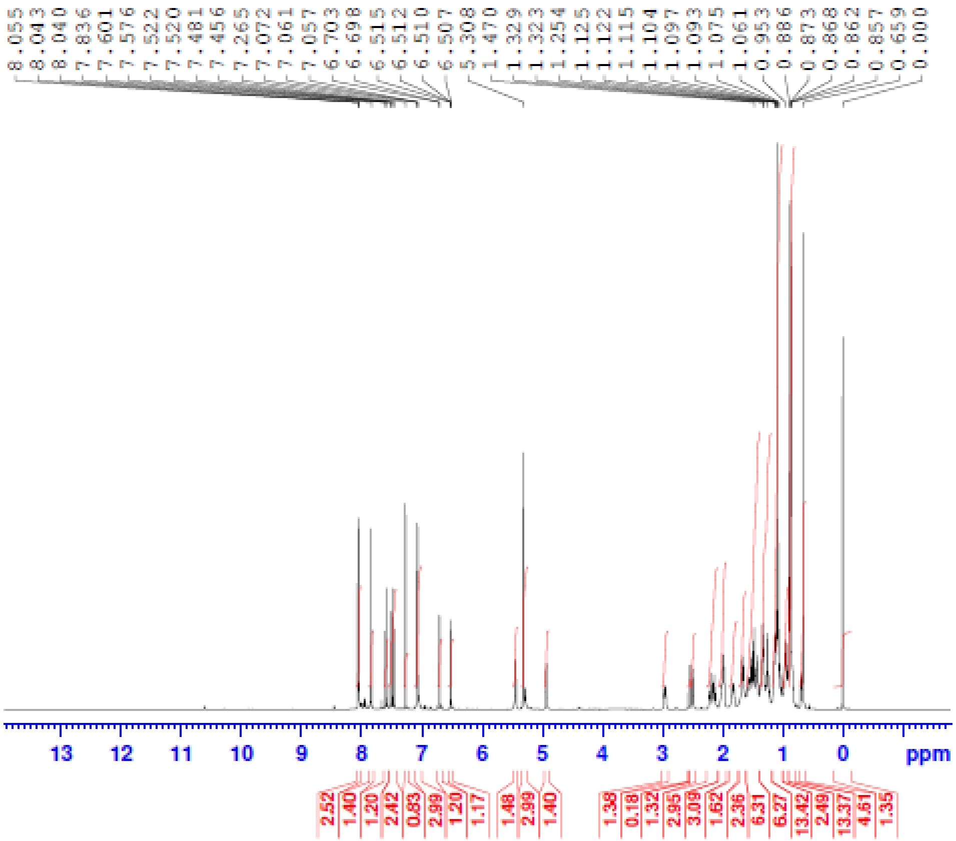

Fig. S11. ^{1}H NMR spectrum of compound **7a**.

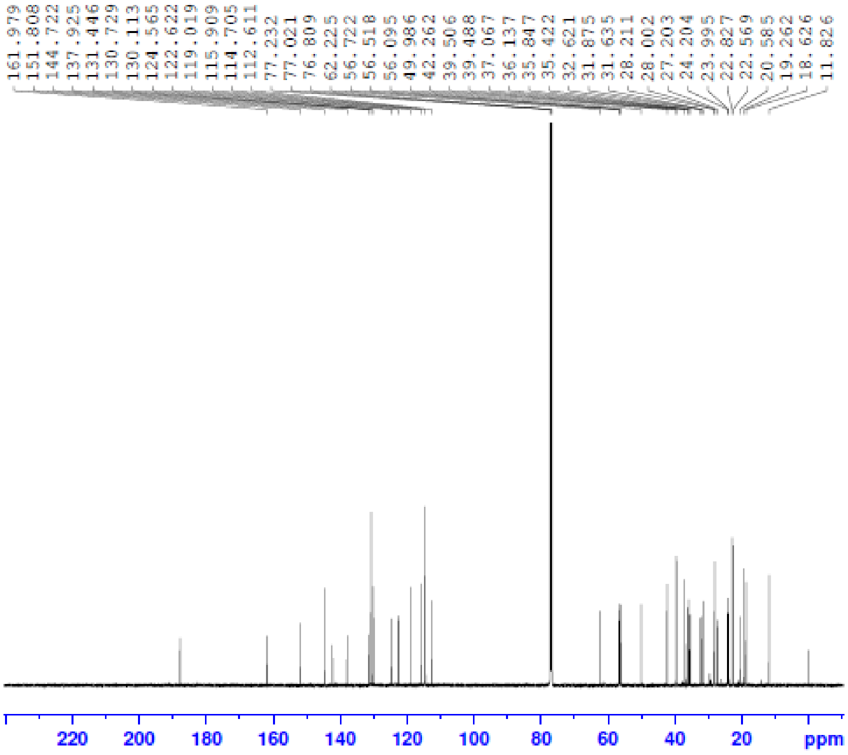

Fig. S12. ^{13}C NMR spectrum of compound **7a**.

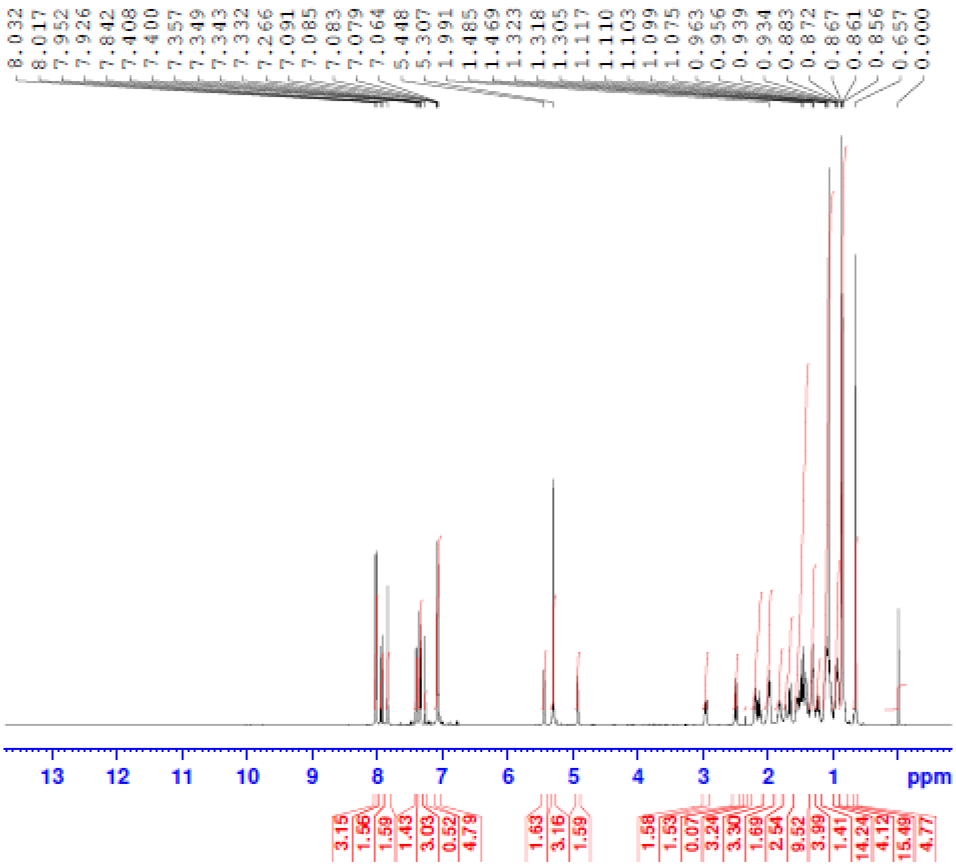

Fig. S13. ^{1}H NMR spectrum of compound **7b**.

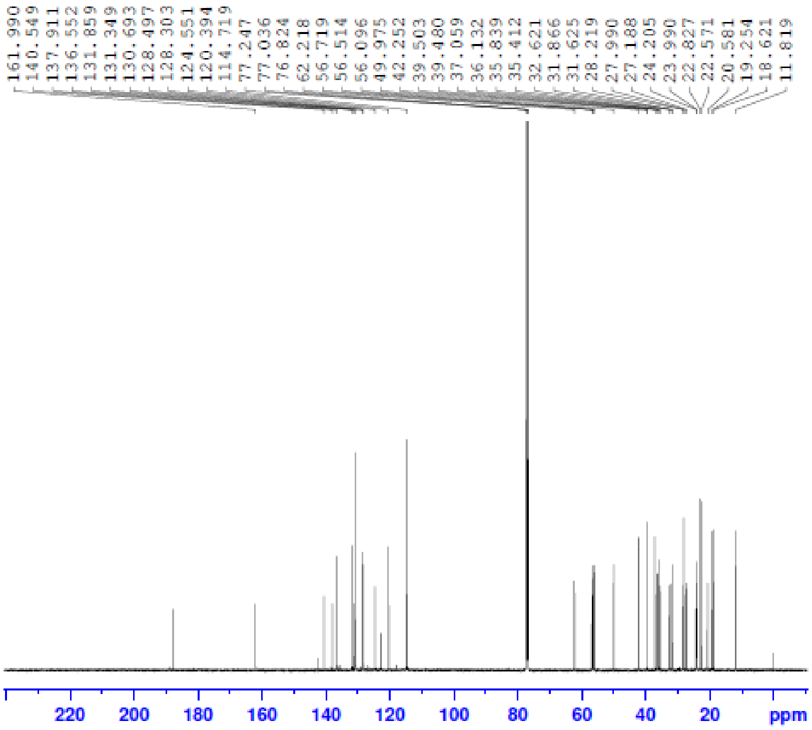

Fig. S14. ^{13}C NMR spectrum of compound **7b**.

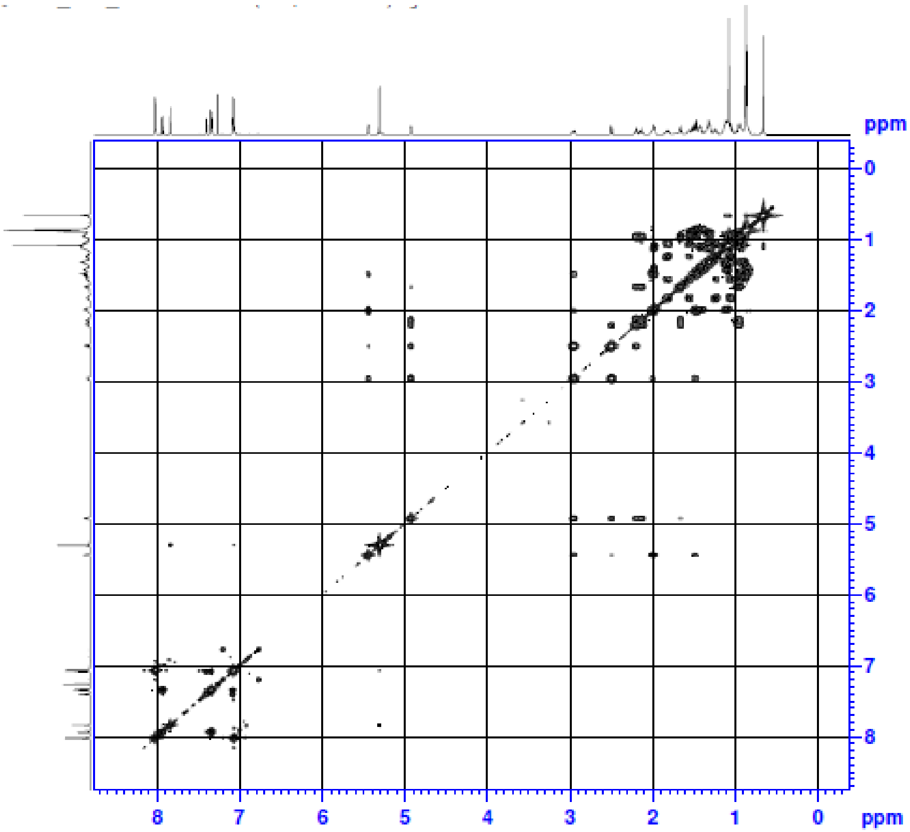

Fig. S15. COSY spectrum of compound **7b**.

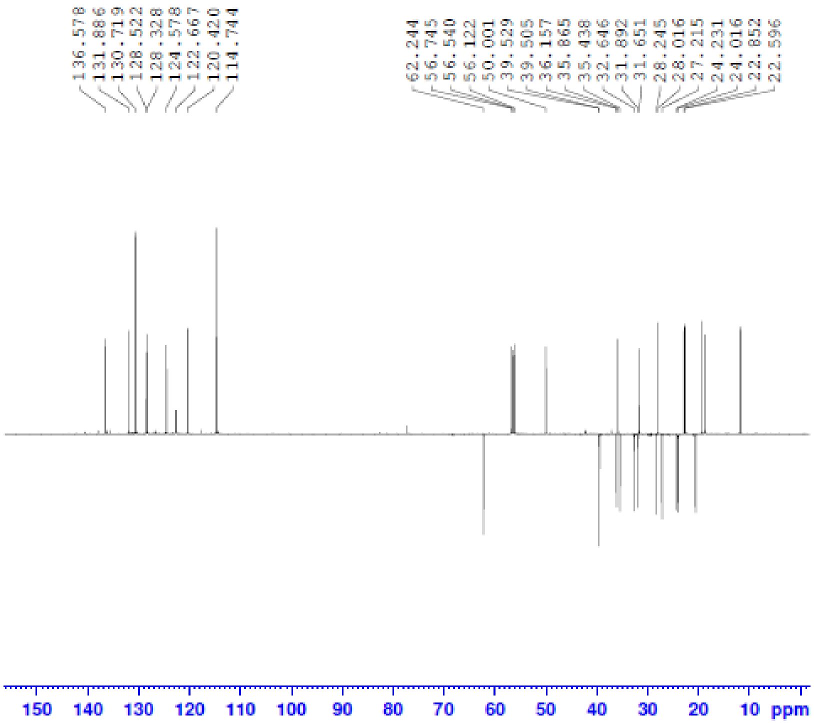

Fig. S16. DEPT−135° spectrum of compound **7b**.

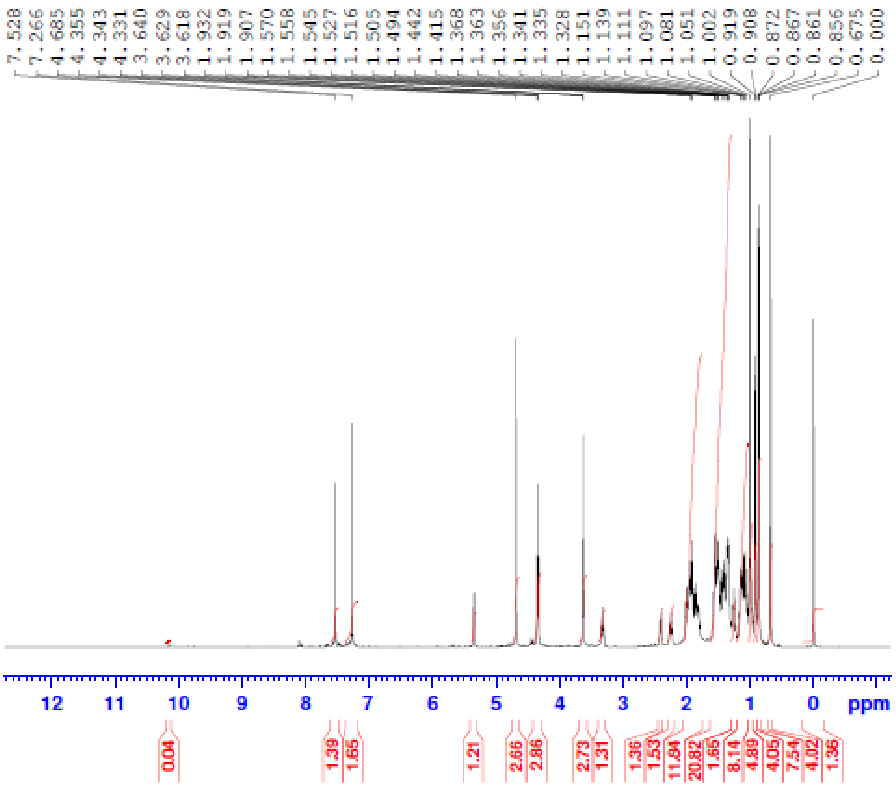

Fig. S17. ^{1}H NMR spectrum of compound **11a**.

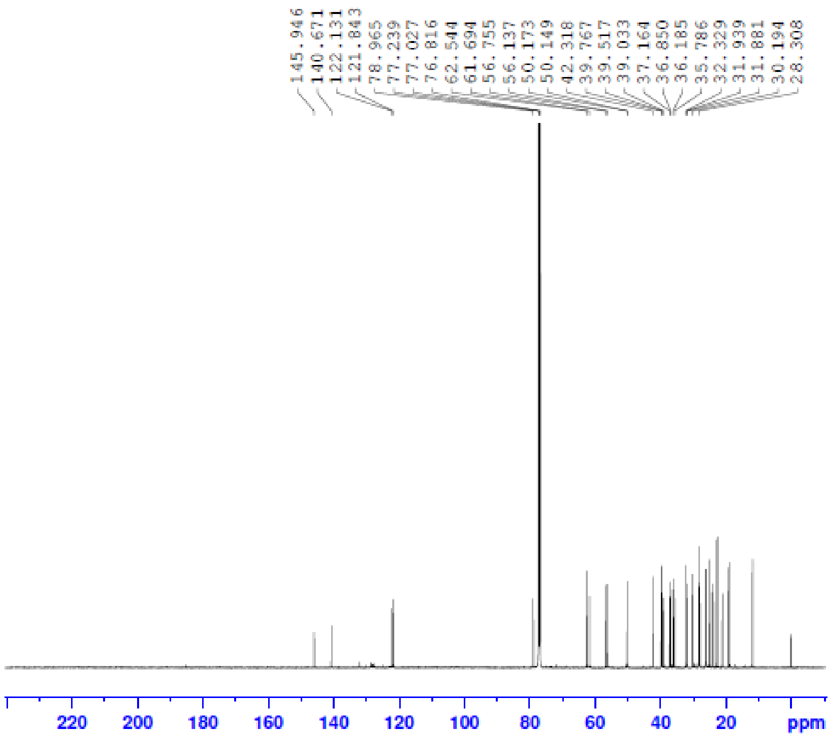

Fig. S18. ^{13}C NMR spectrum of compound **11a**.

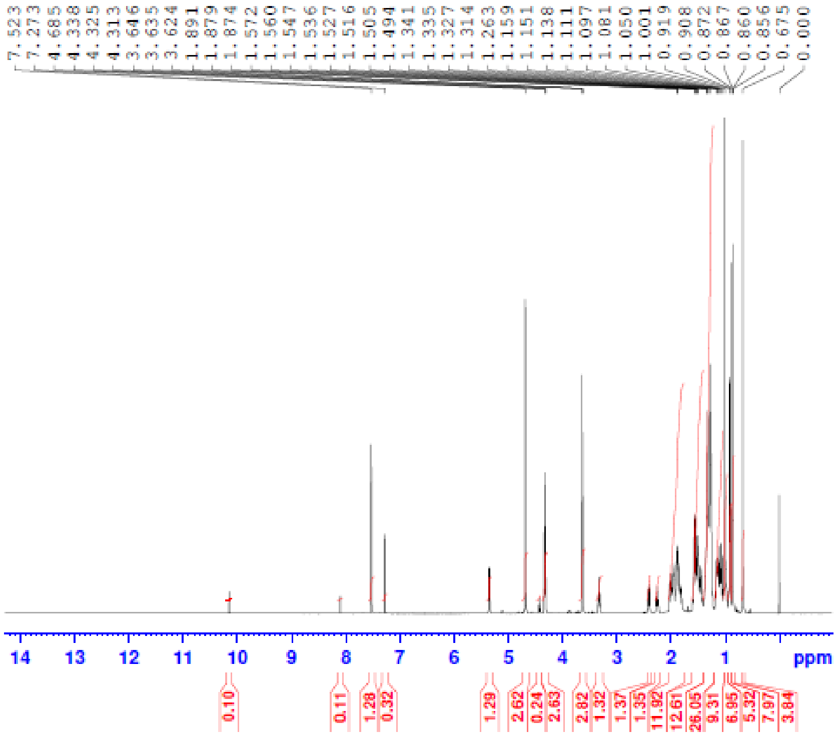

Fig. S19. ^{1}H NMR spectrum of compound **11b**.

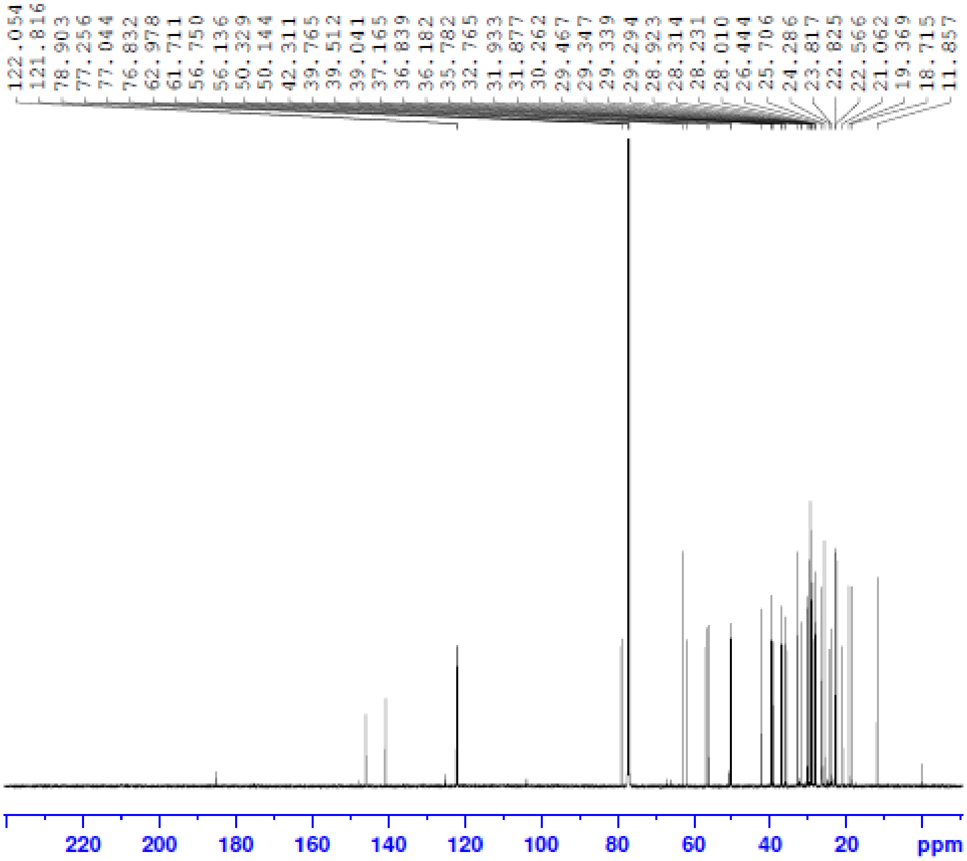

Fig. S20. ^{13}C NMR spectrum of compound **11b**.

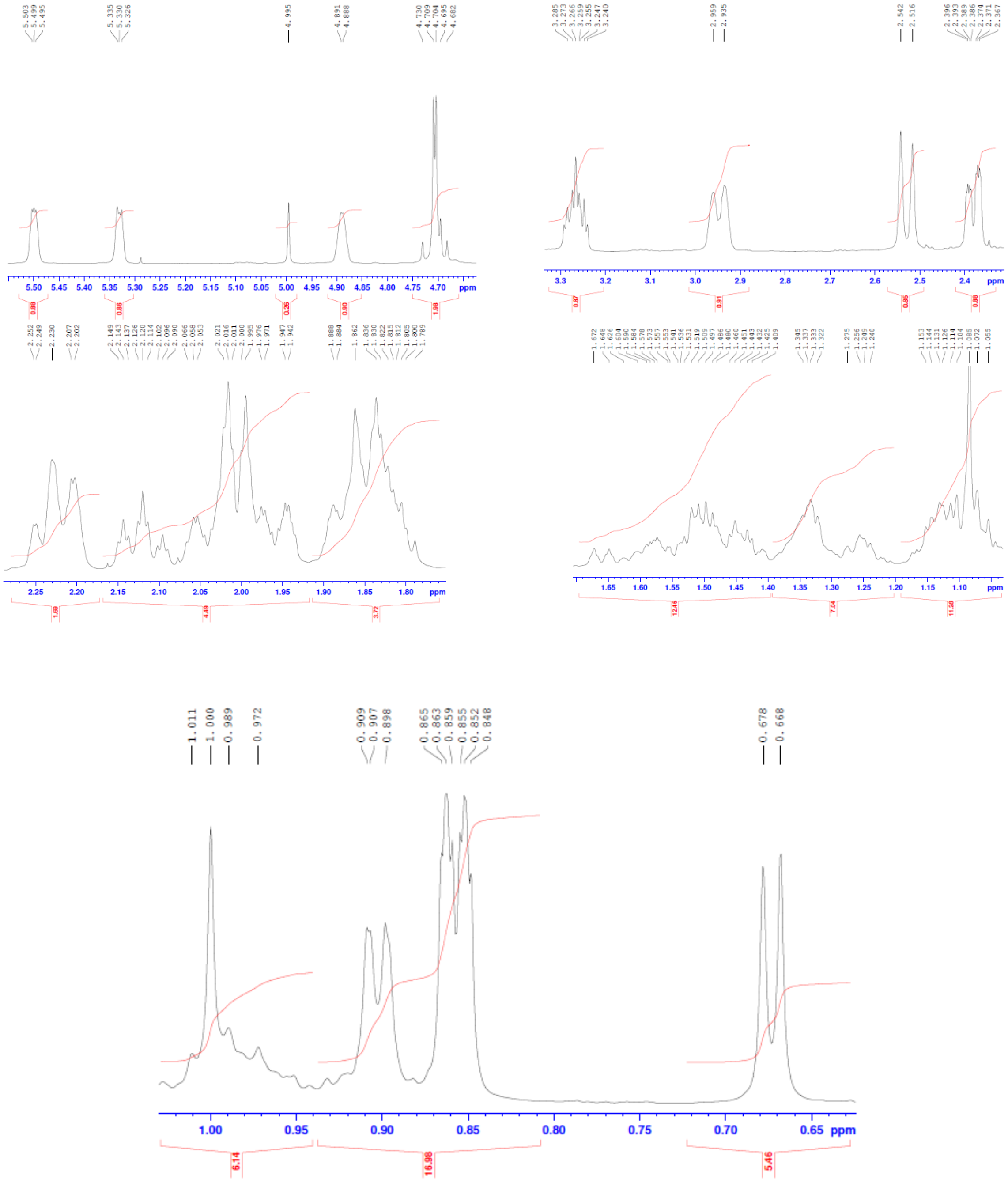

Figs. S21. ^{1}H NMR spectrum of compound **13**.

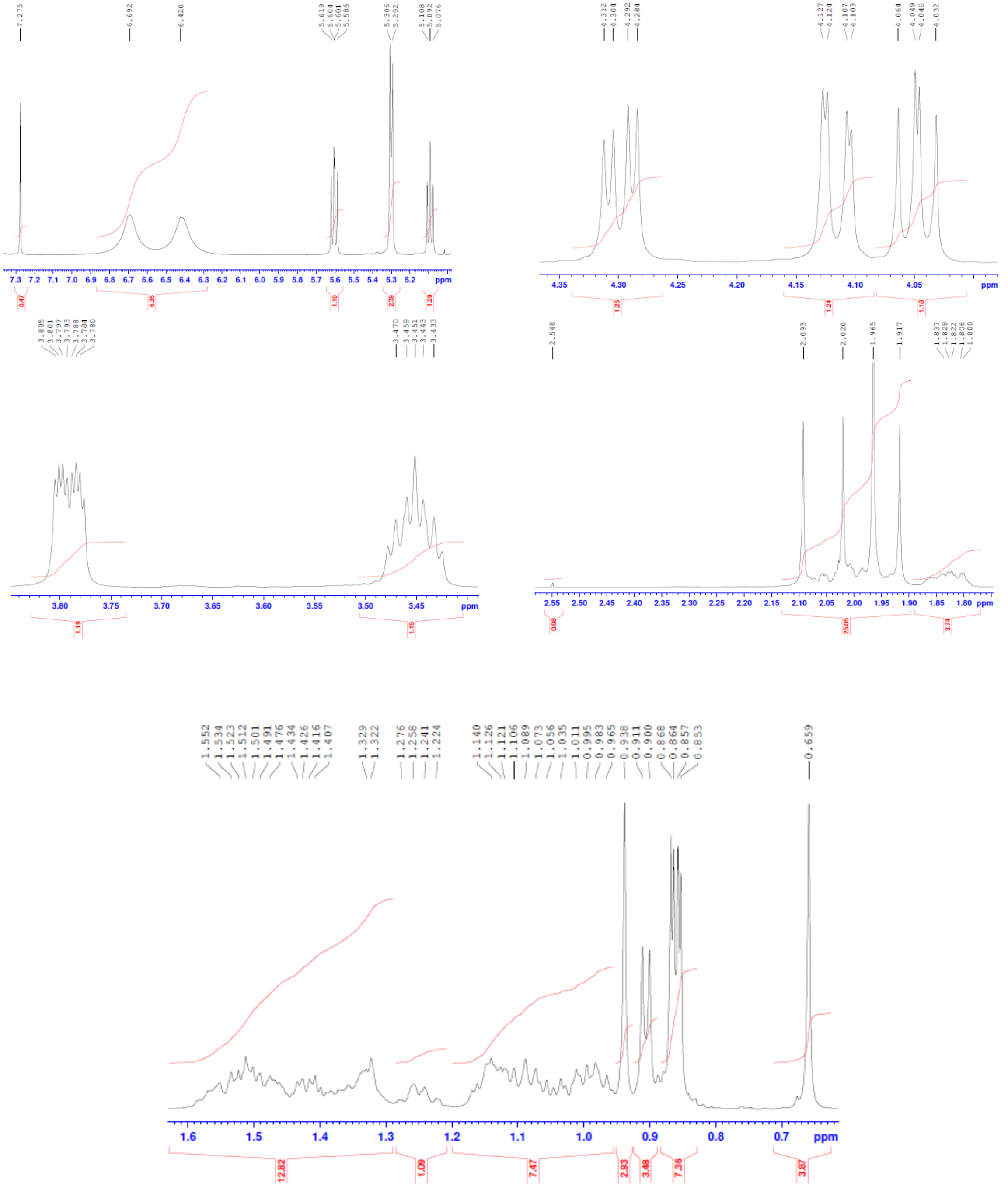

Figs. S22. ^{1}H NMR spectrum of compound **15**.

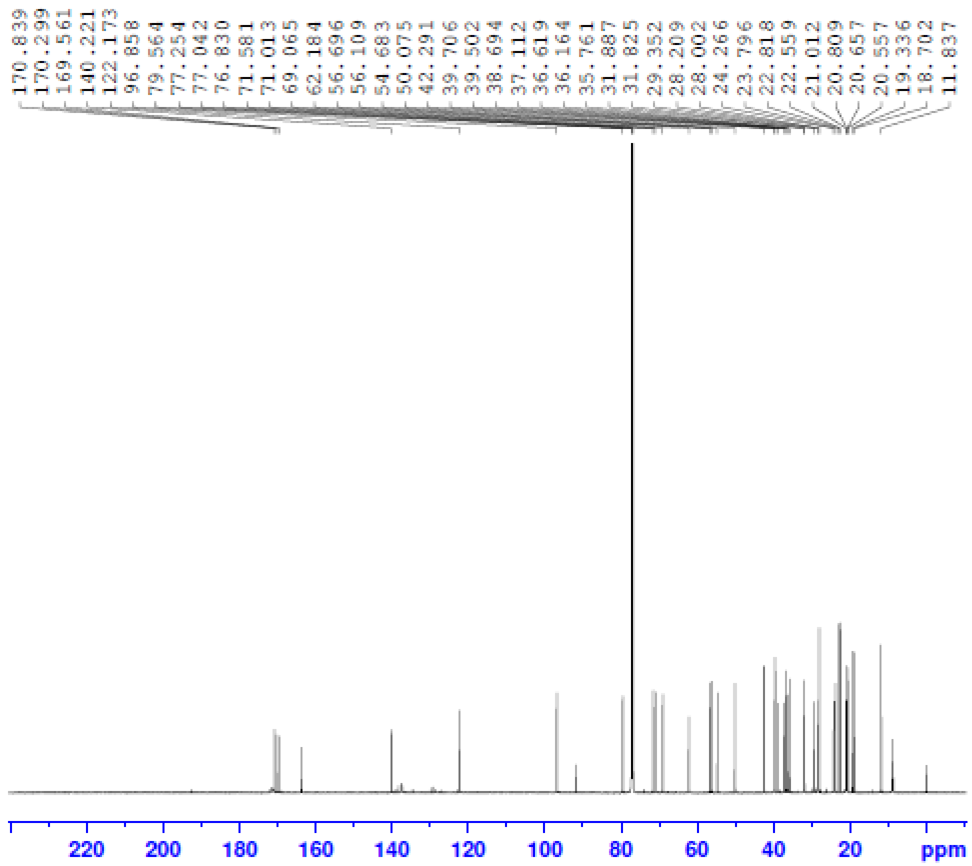

Figs. S23. ^{13}C NMR spectrum of compound **15**.

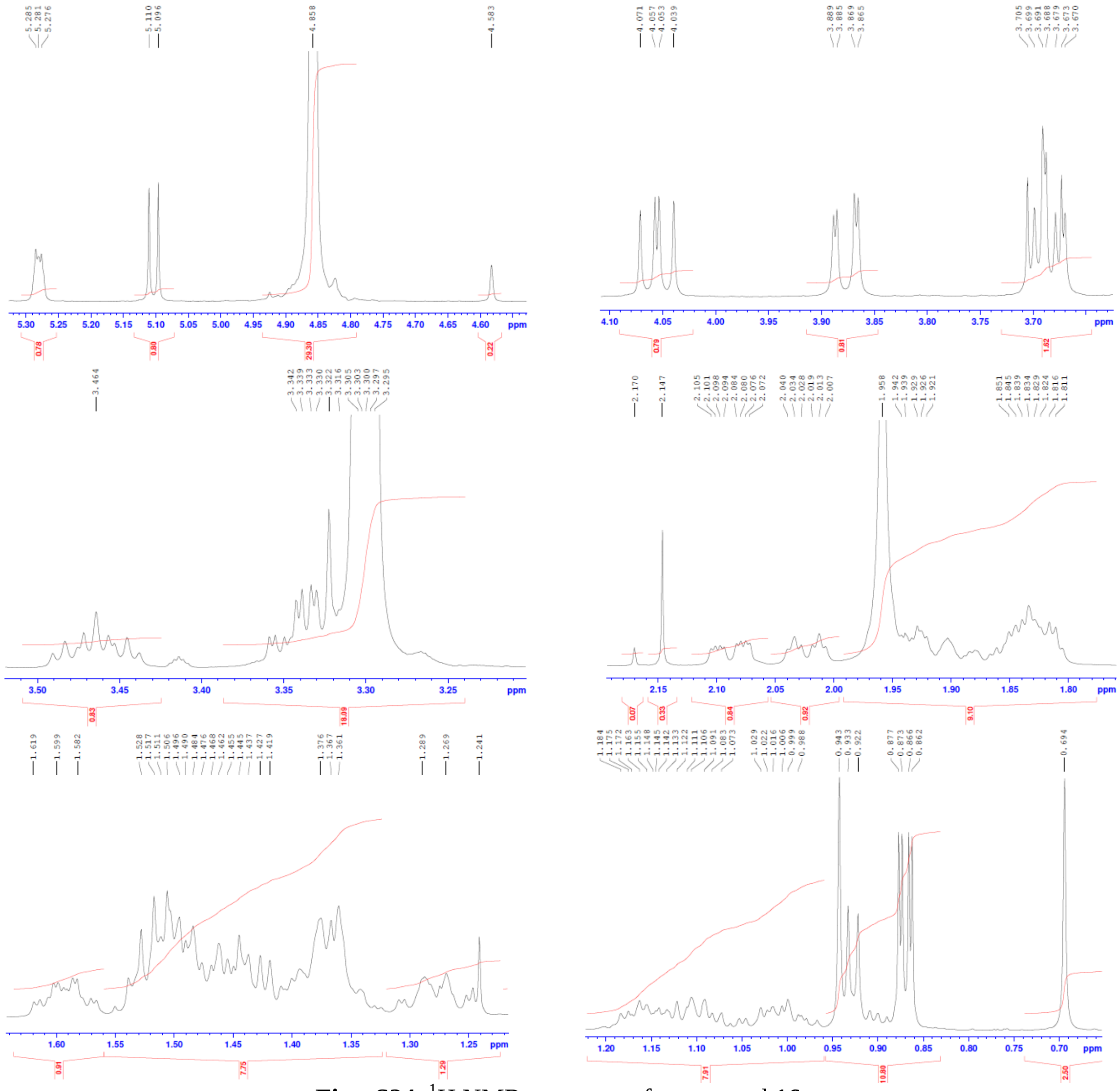

Figs. S24. ^{1}H NMR spectrum of compound **16**.

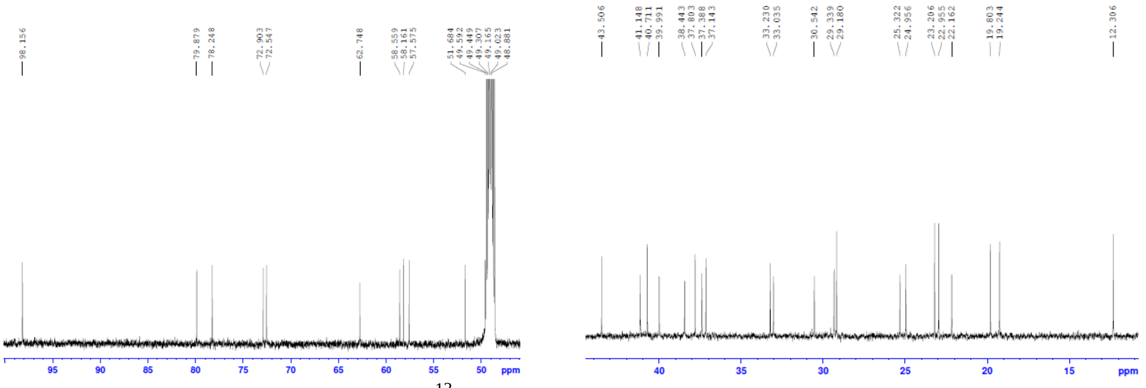

Figs. S25. ^{13}C NMR spectrum of compound **16**.

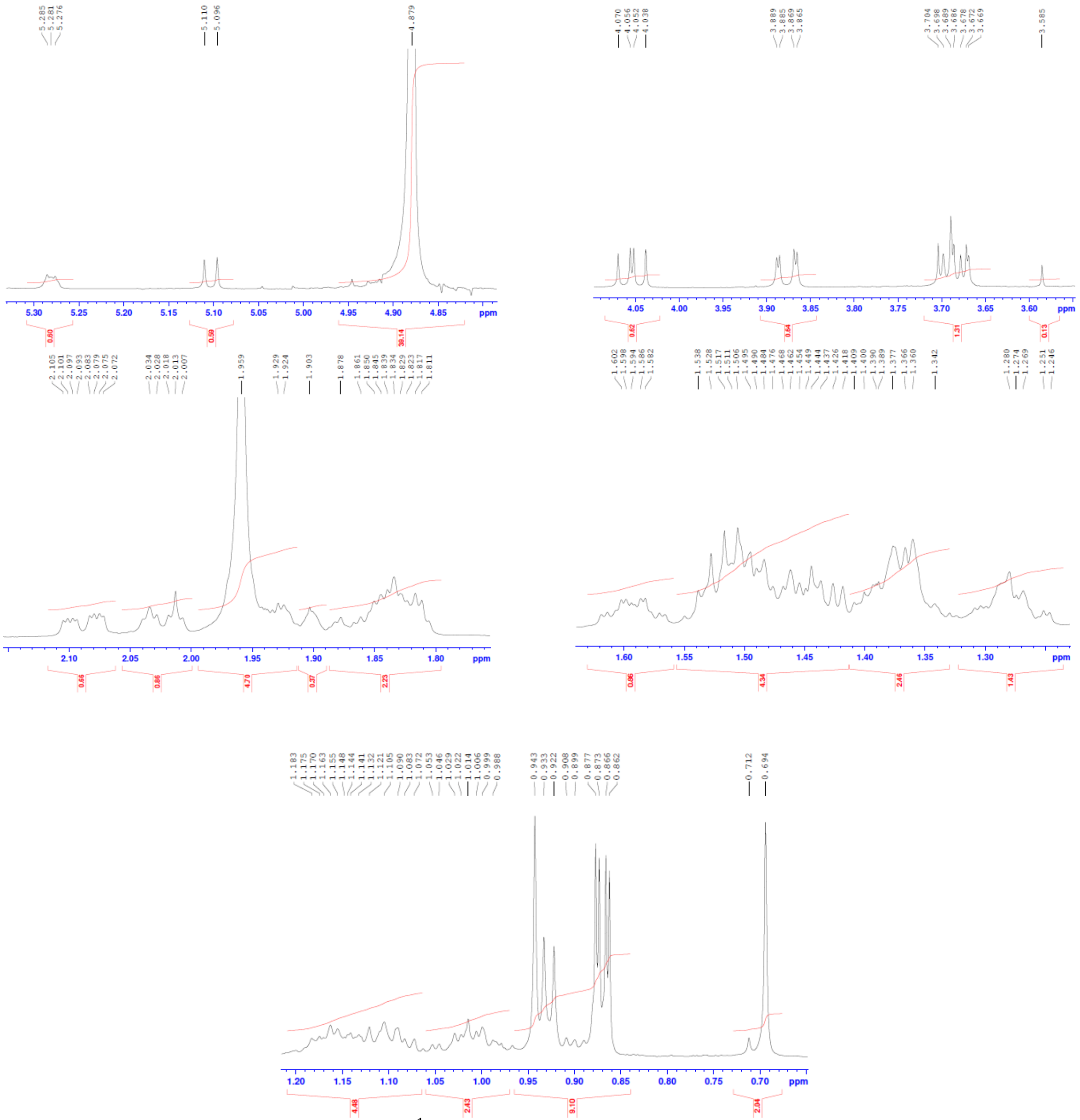

Figs. S26. ^{1}H NMR spectrum of compound **17**.

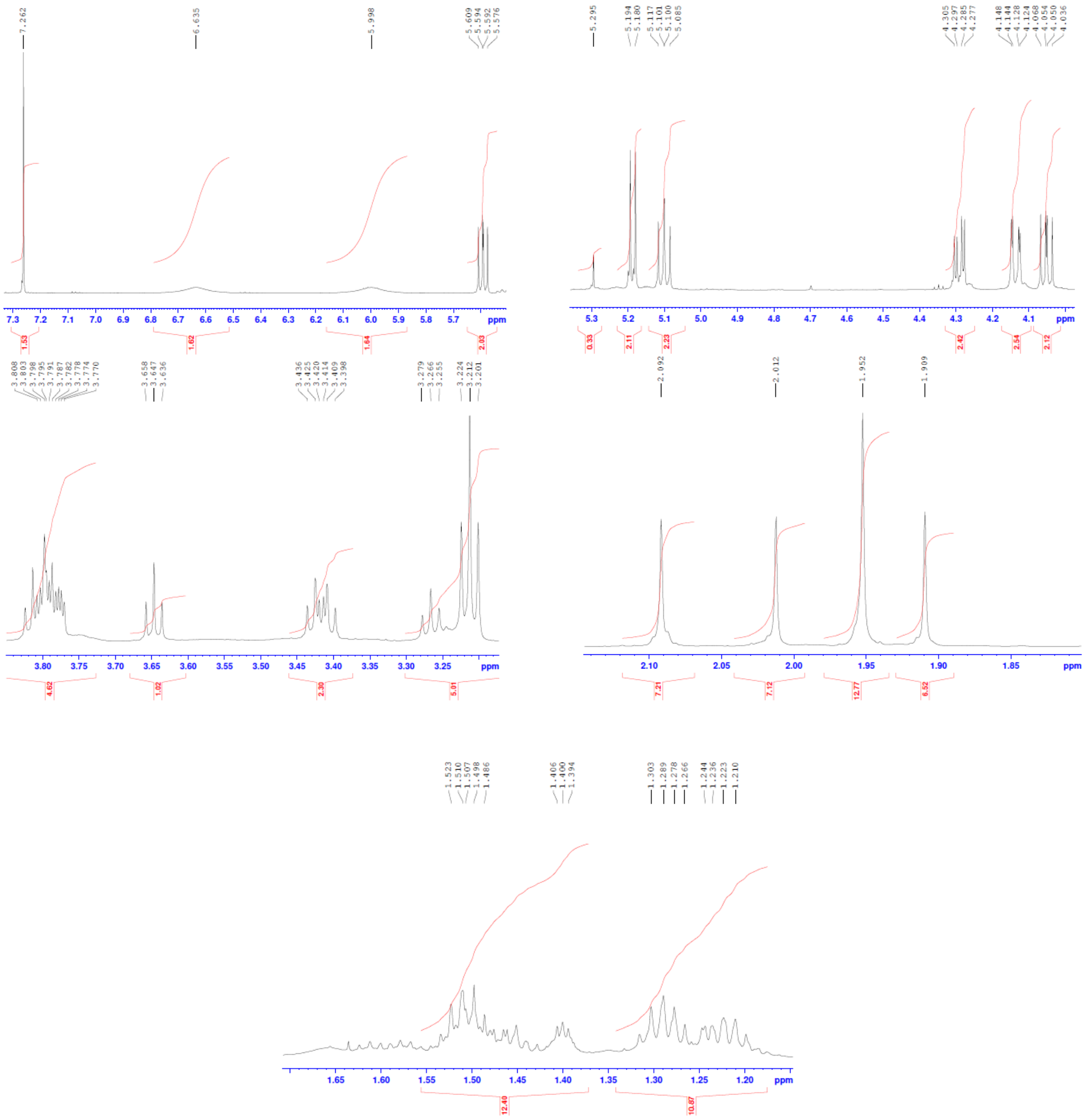

Figs. S27. ^{1}H NMR spectrum of compound **18**.

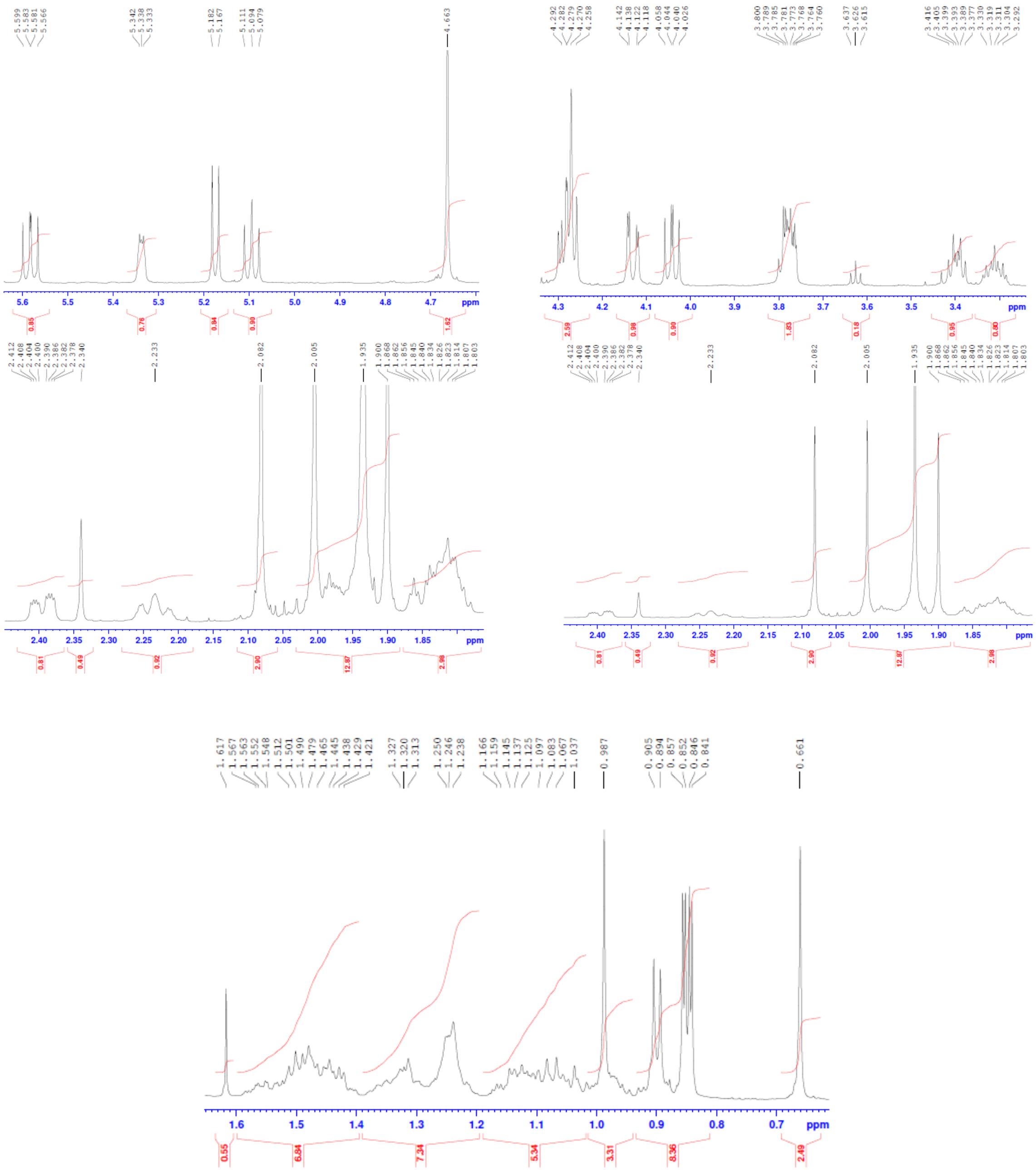

Figs. S28. ¹H NMR spectrum of compound **19**.

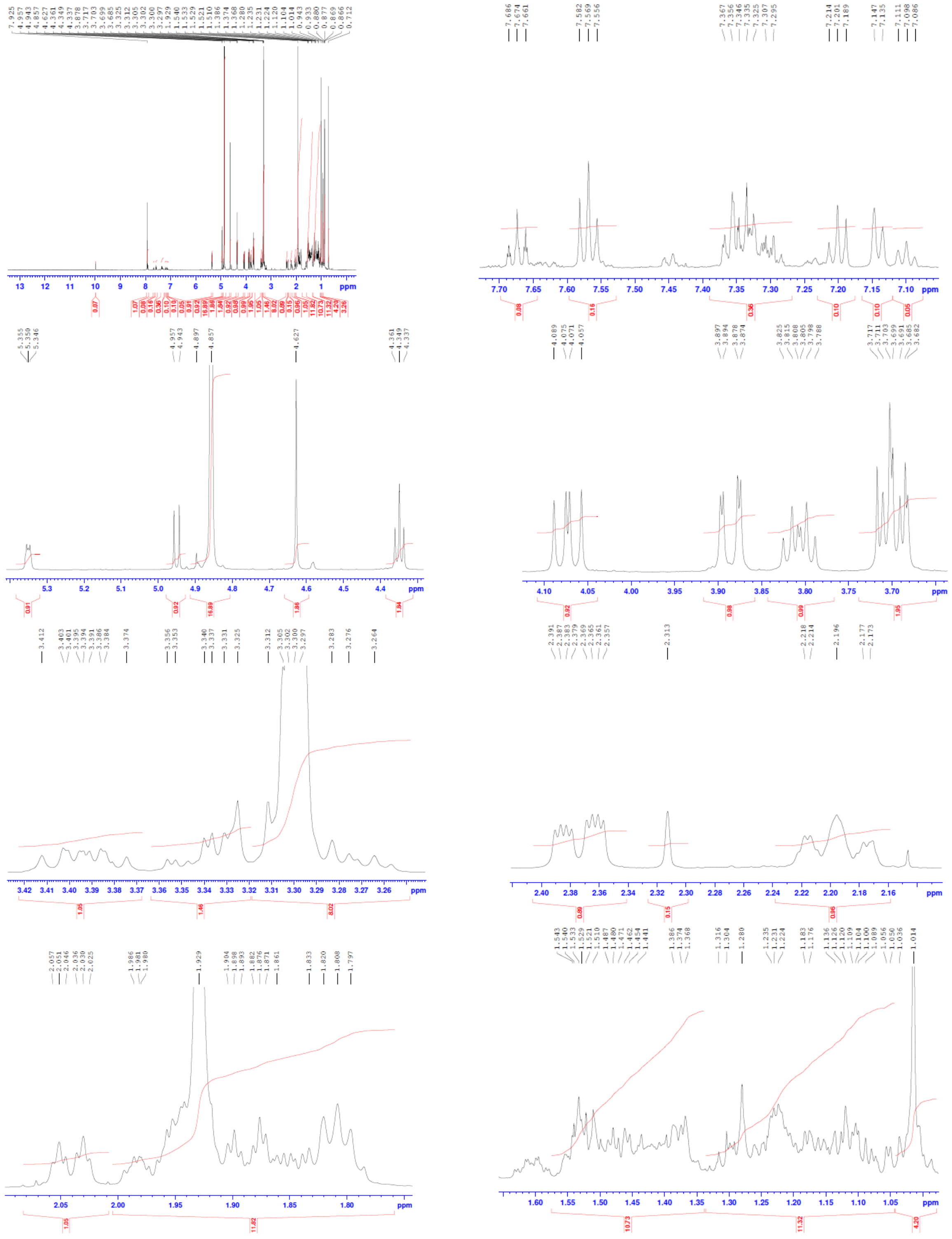

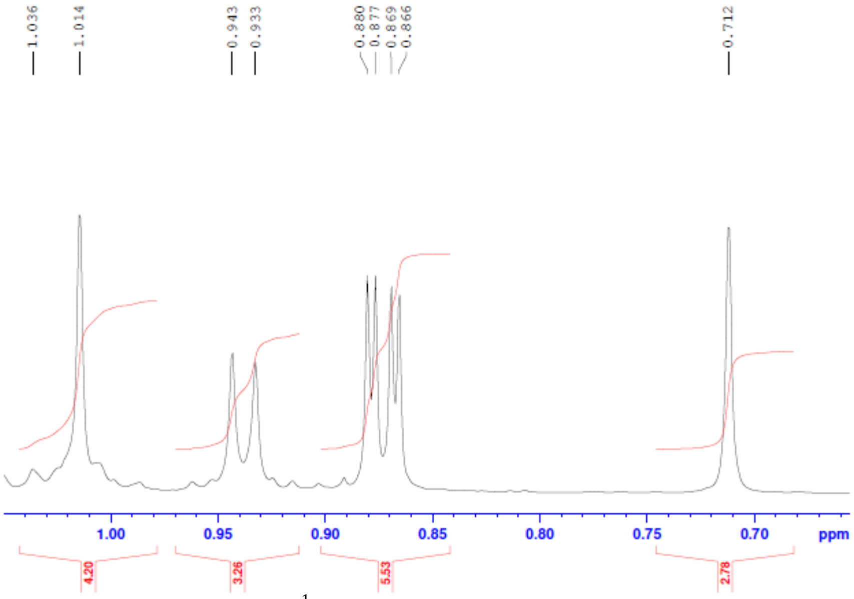

Figs. S29. ^{1}H NMR spectrum of compound **20**.

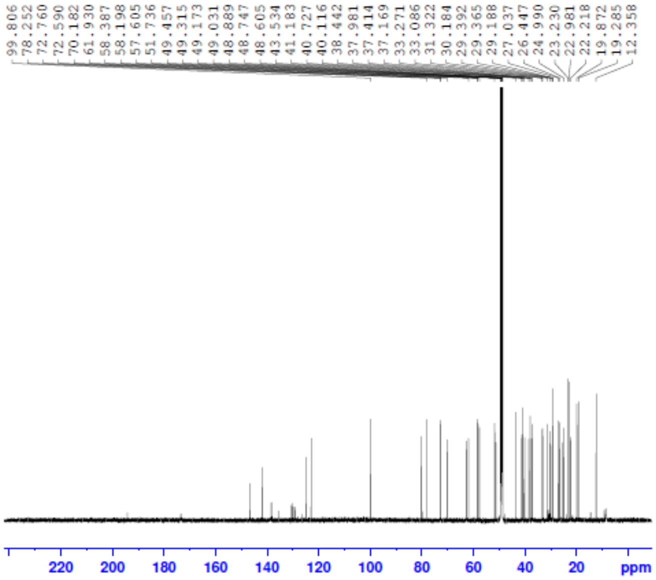

Fig. S30. ^{13}C NMR spectrum of compound **20**.

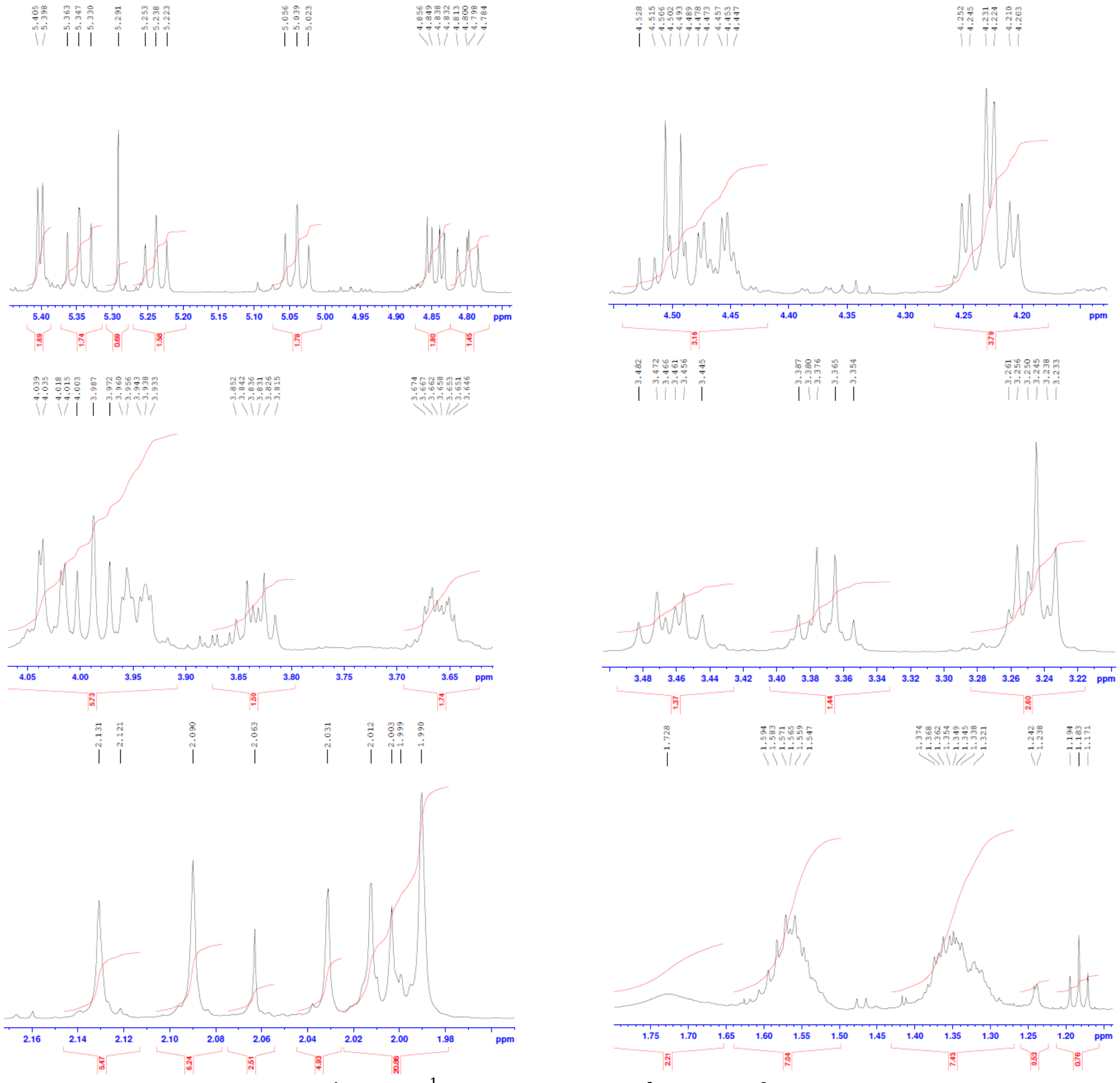

Figs. S31. ^{1}H NMR spectrum of compound **22**.

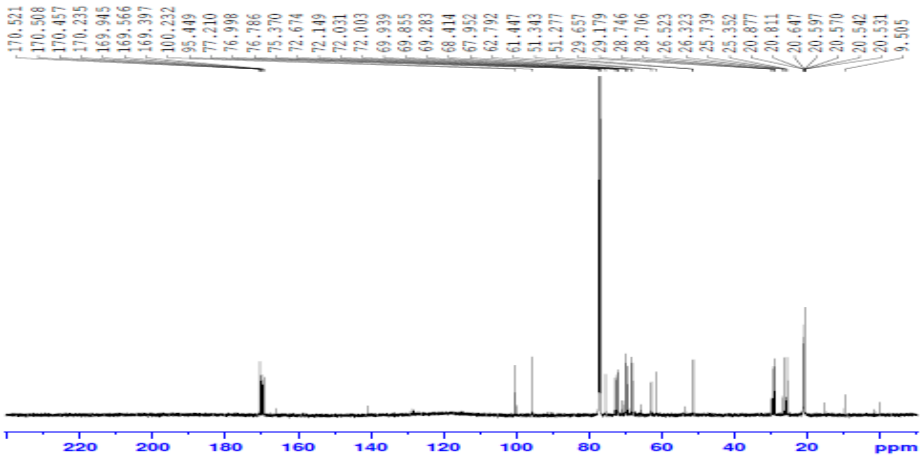

Fig. S32. ^{13}C NMR spectrum of compound **22**.

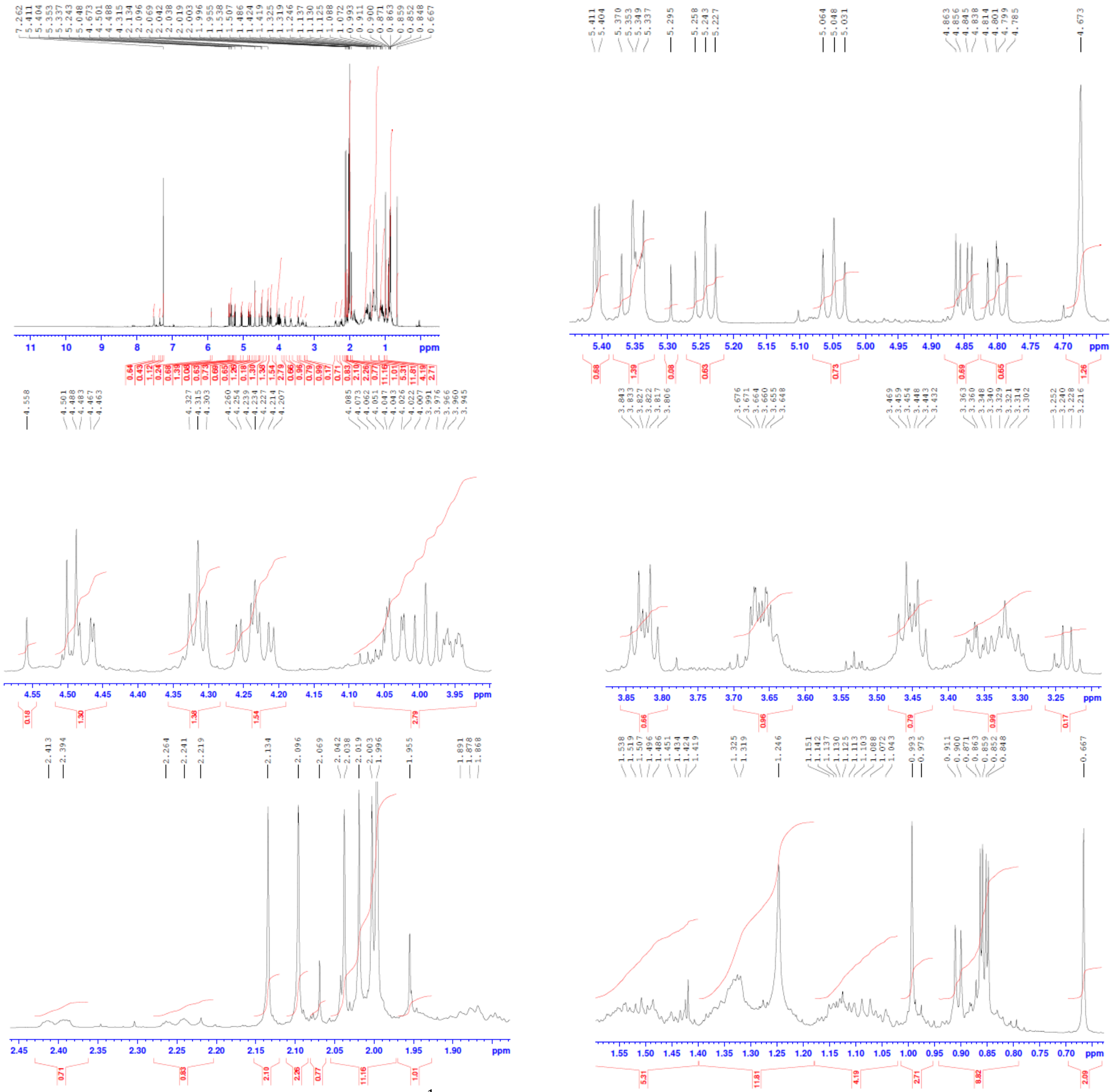

Figs. S33. ^{1}H NMR spectrum of compound **23**.

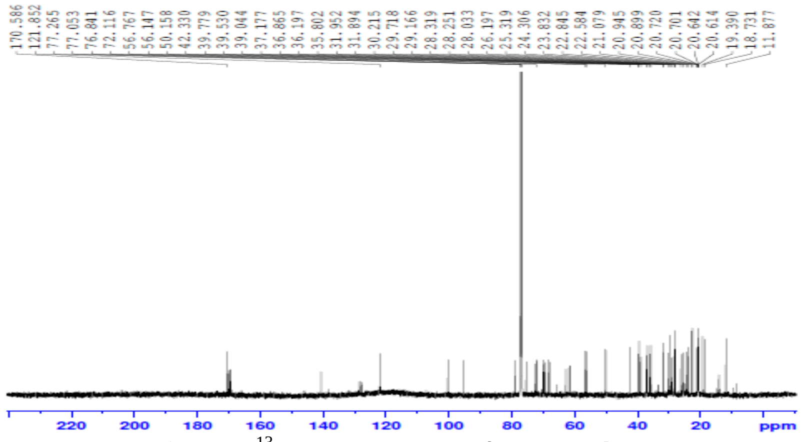

Fig. S34. ^{13}C NMR spectrum of compound **23**.

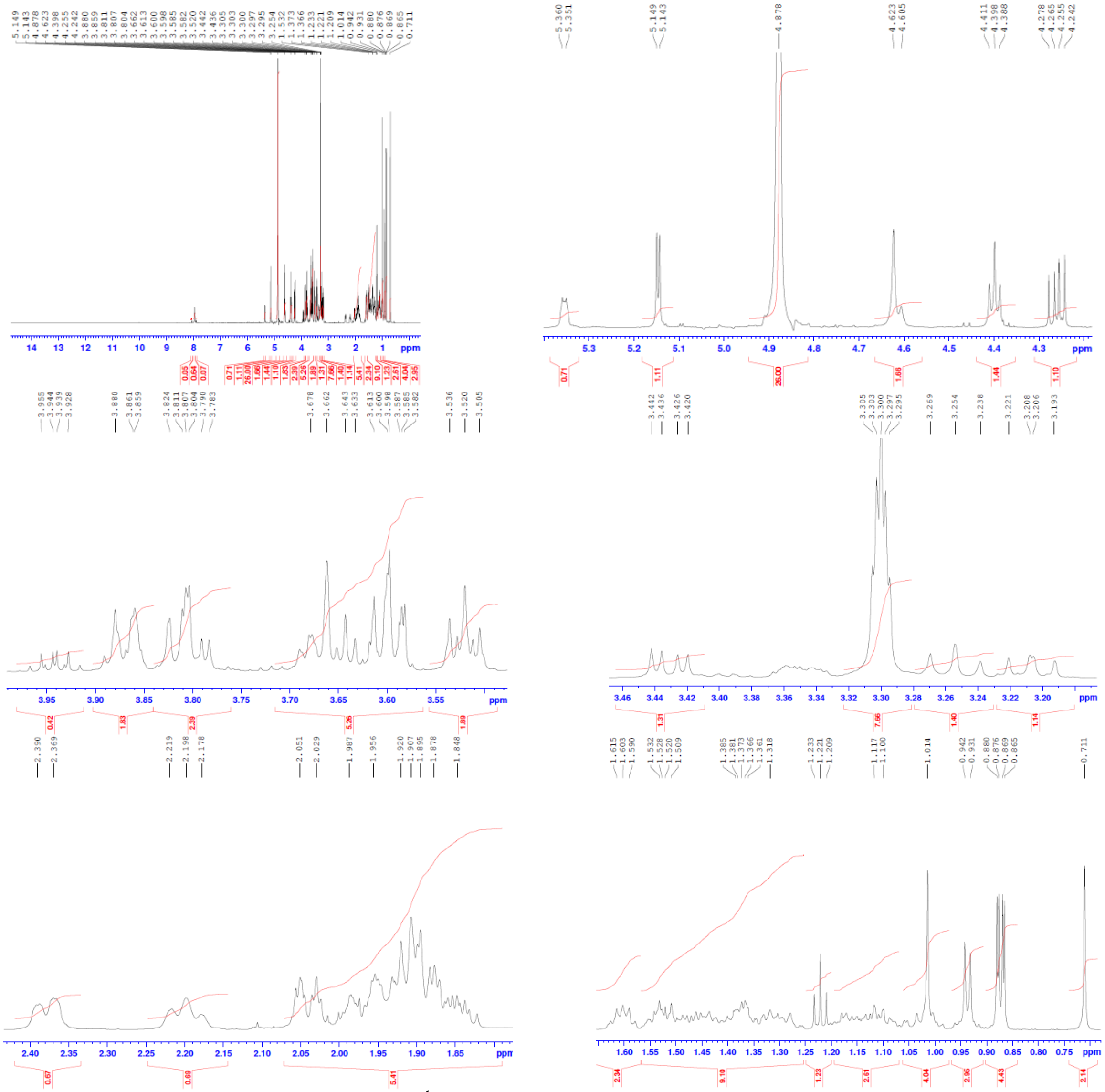

Figs. S35. ^{1}H NMR spectrum of compound **24**.

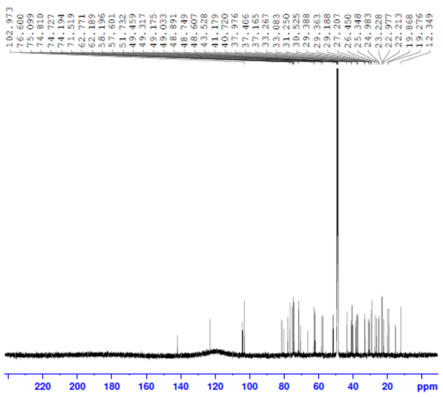

Fig. S36. ^{13}C NMR spectrum of compound **24**.

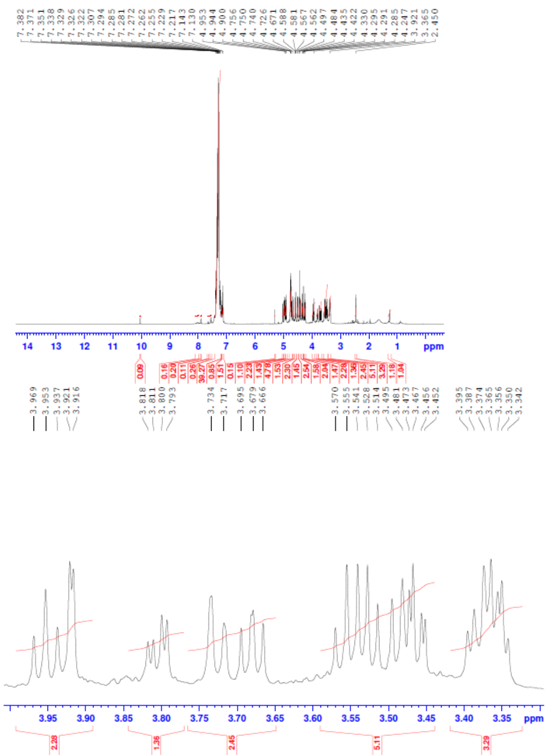

Figs. S37. ^{1}H NMR spectrum of compound **26**.

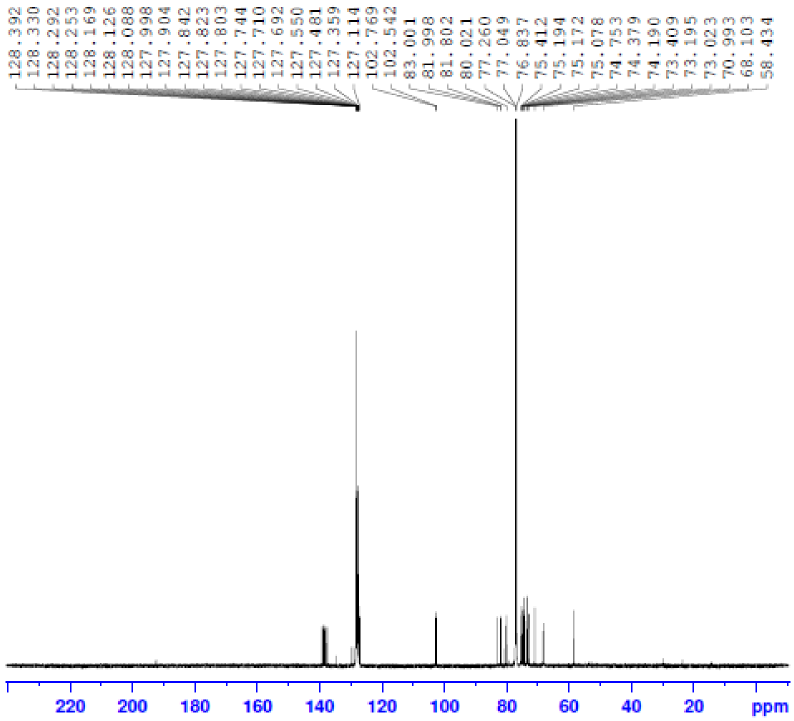

Fig. S38. ^{13}C NMR spectrum of compound **26**.

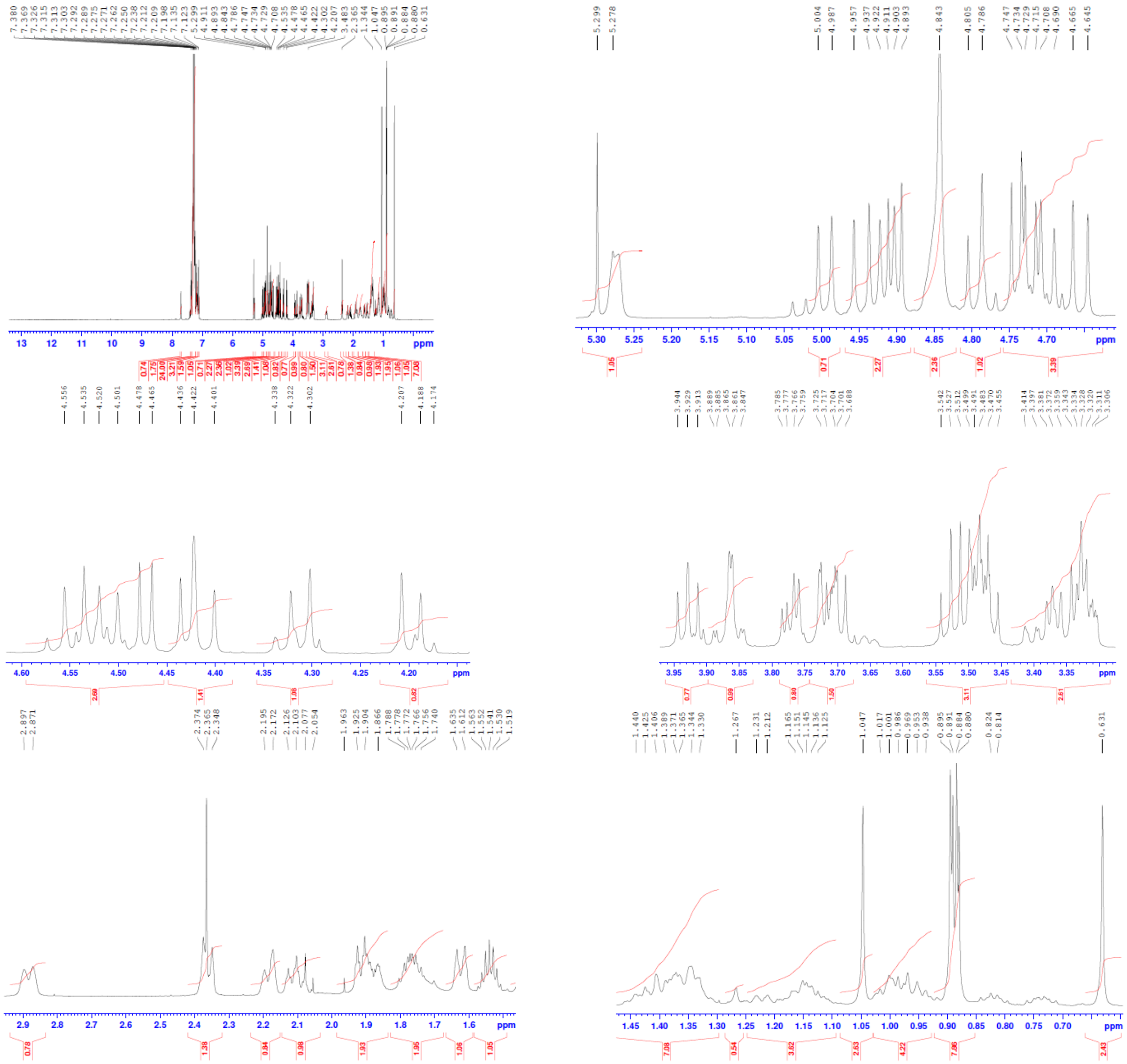

Figs. S39. ^{1}H NMR spectrum of compound **27**.

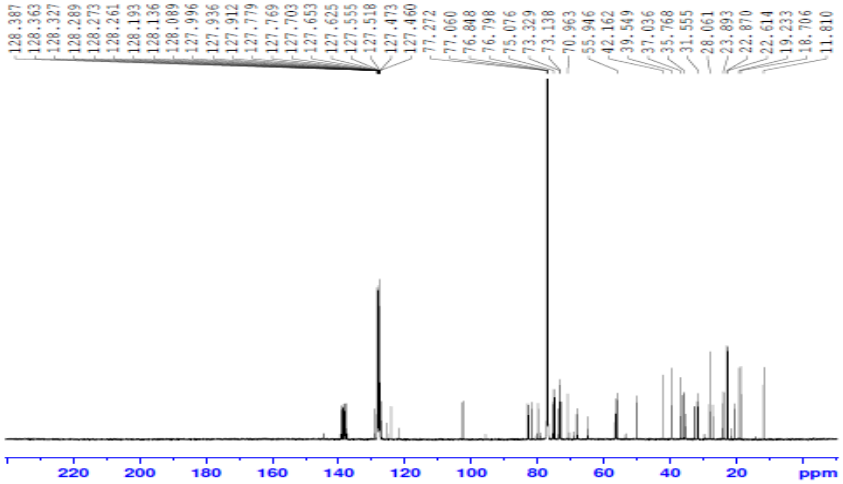

Figs. S40. ^{13}C NMR spectrum of **27**.

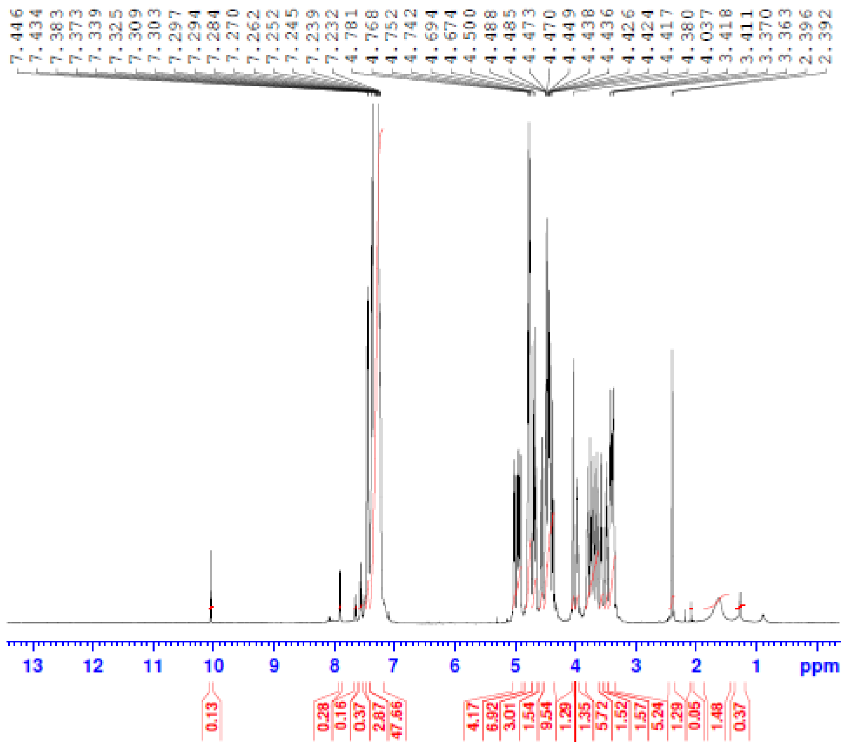

Fig. S41. ^{1}H NMR spectrum of compound **30**.

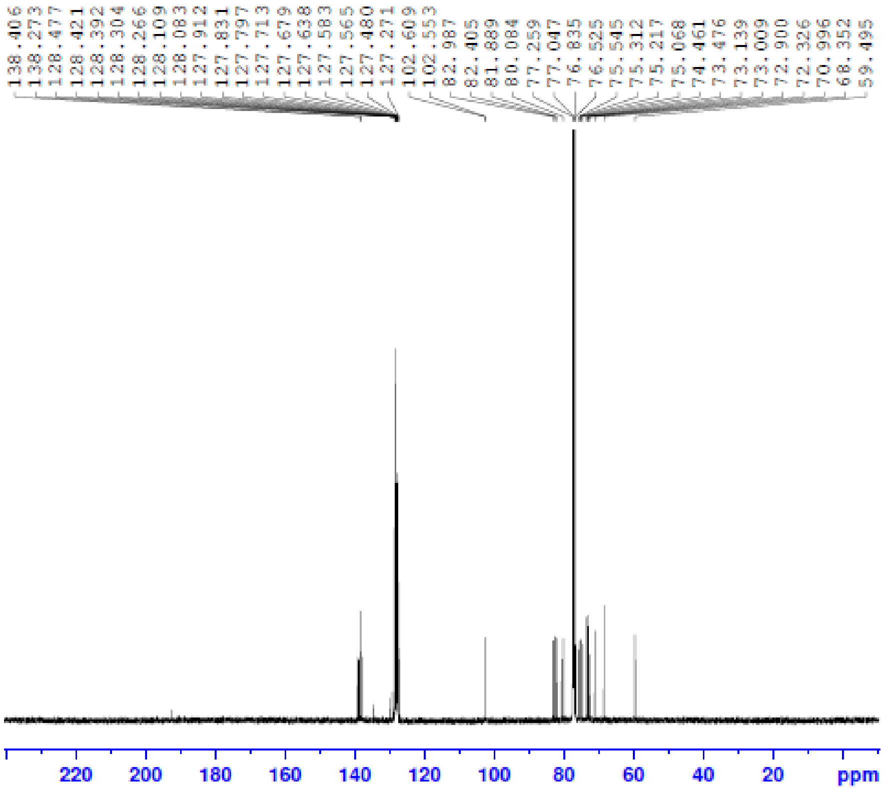

Fig. S42. ^{13}C NMR spectrum of compound **30**.

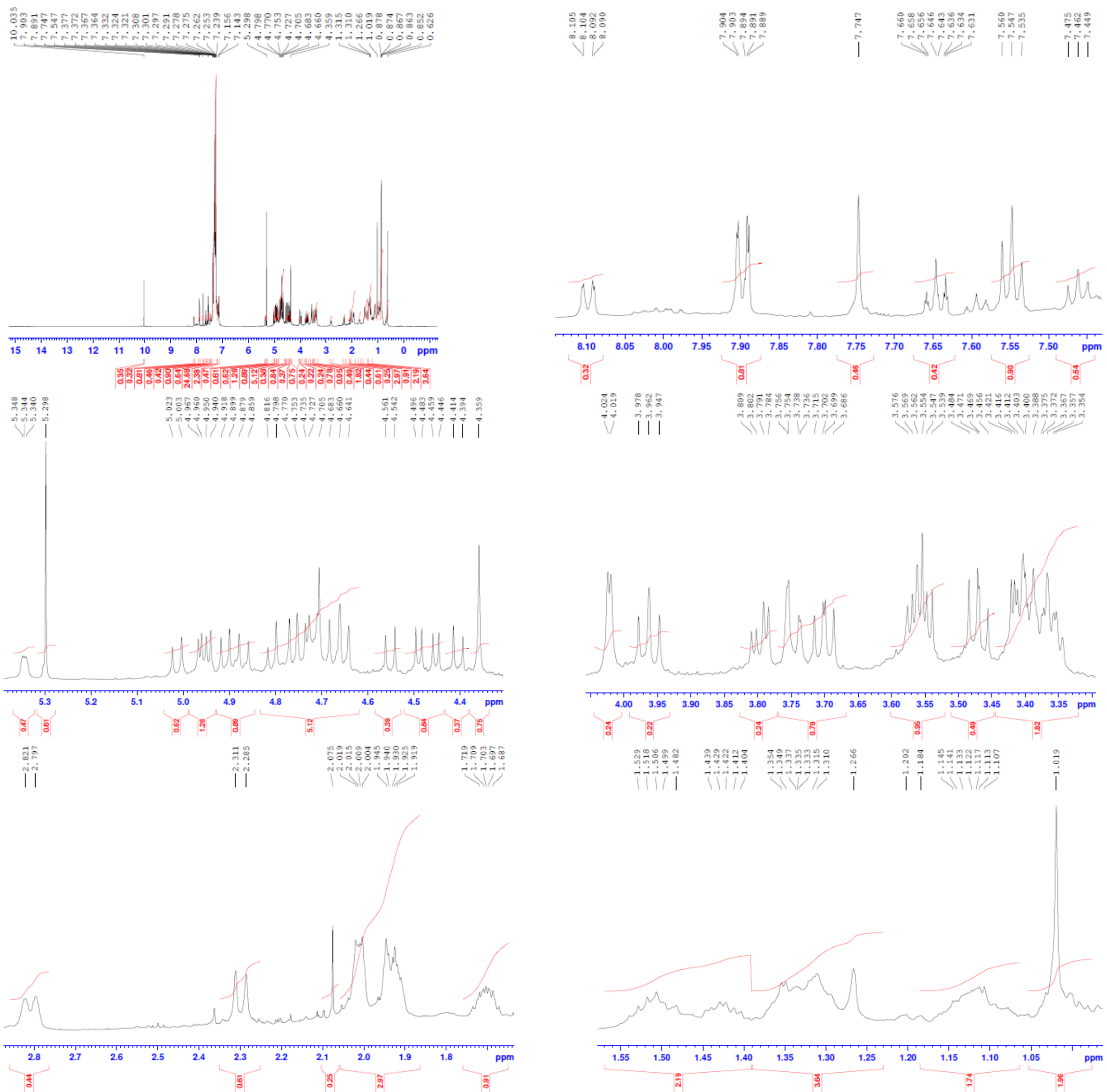

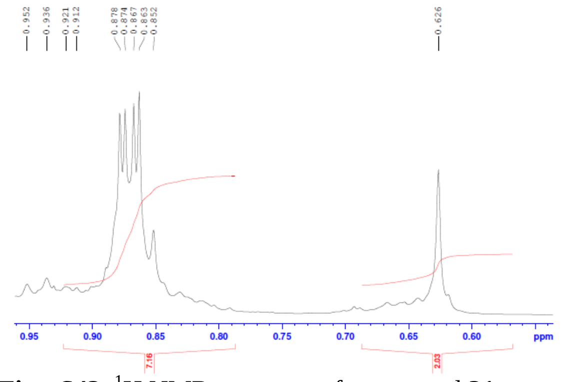

Figs. S43. ^{1}H NMR spectrum of compound **31**.

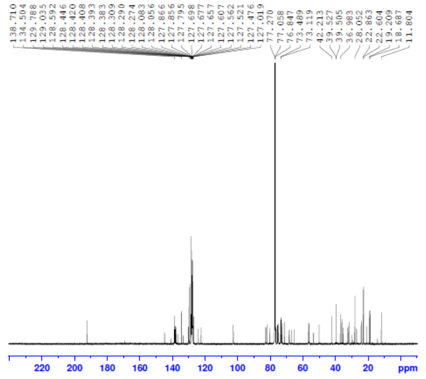

Fig. S44. ^{13}C NMR spectrum of compound **31**.

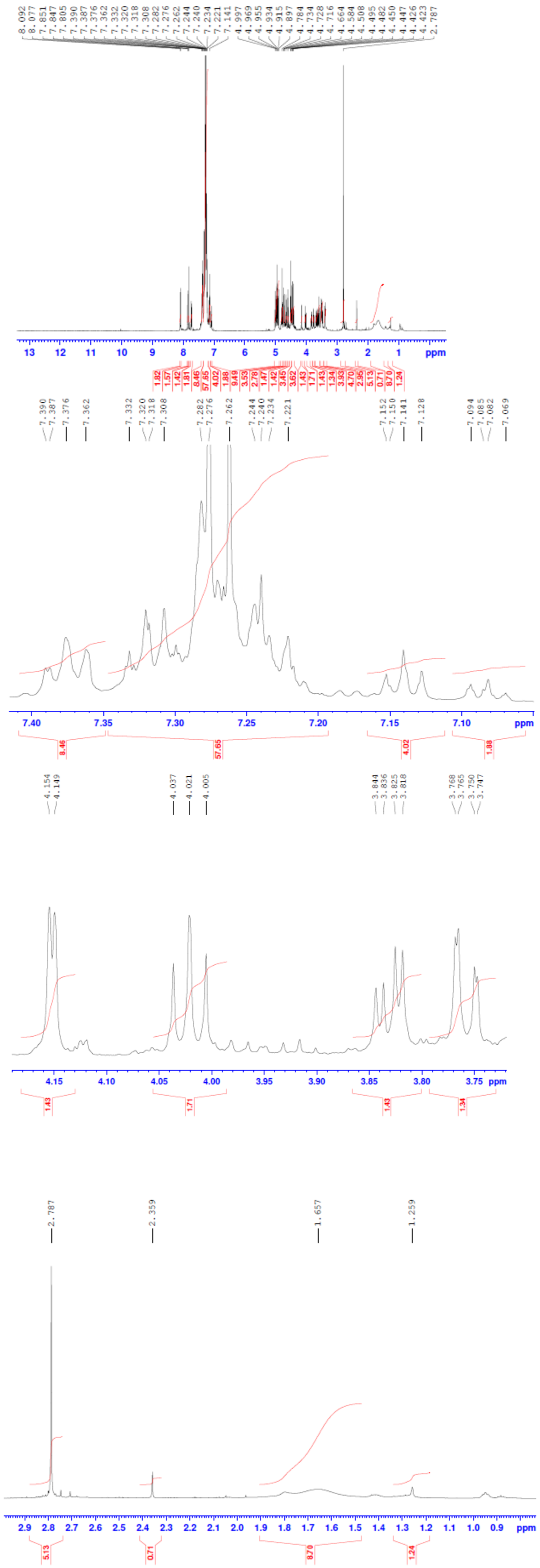

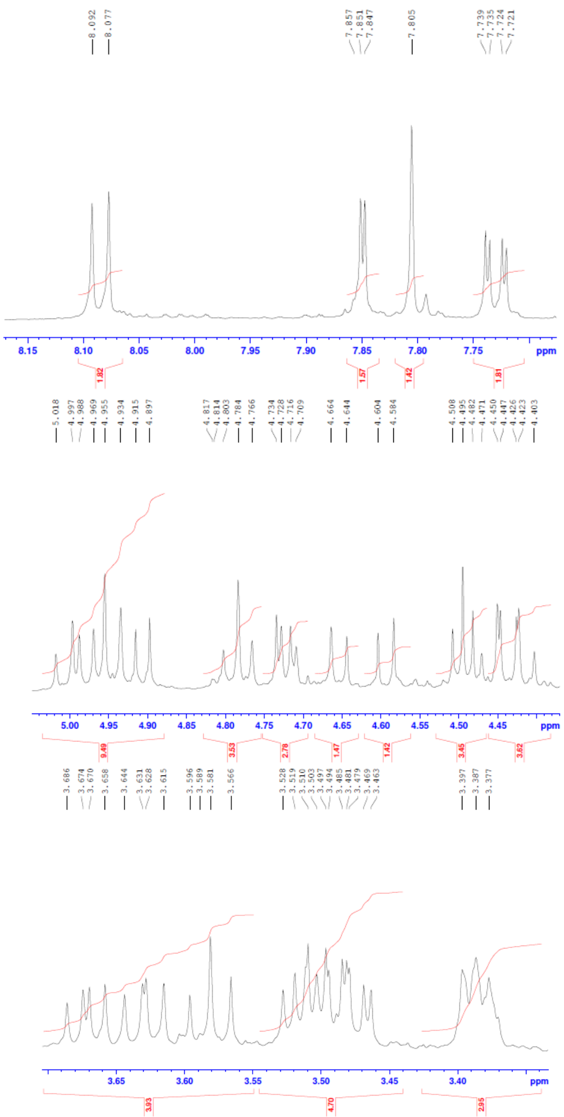

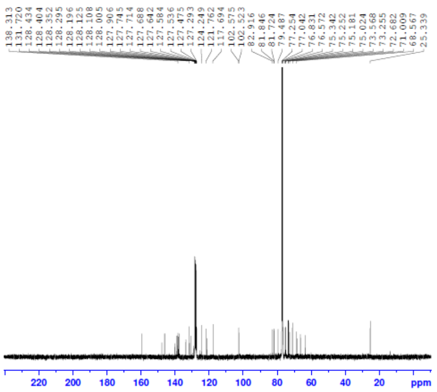

Figs. S45. ^{1}H NMR spectrum of compound **36**.

Fig. S46. ^{13}C NMR spectrum of compound **36**.

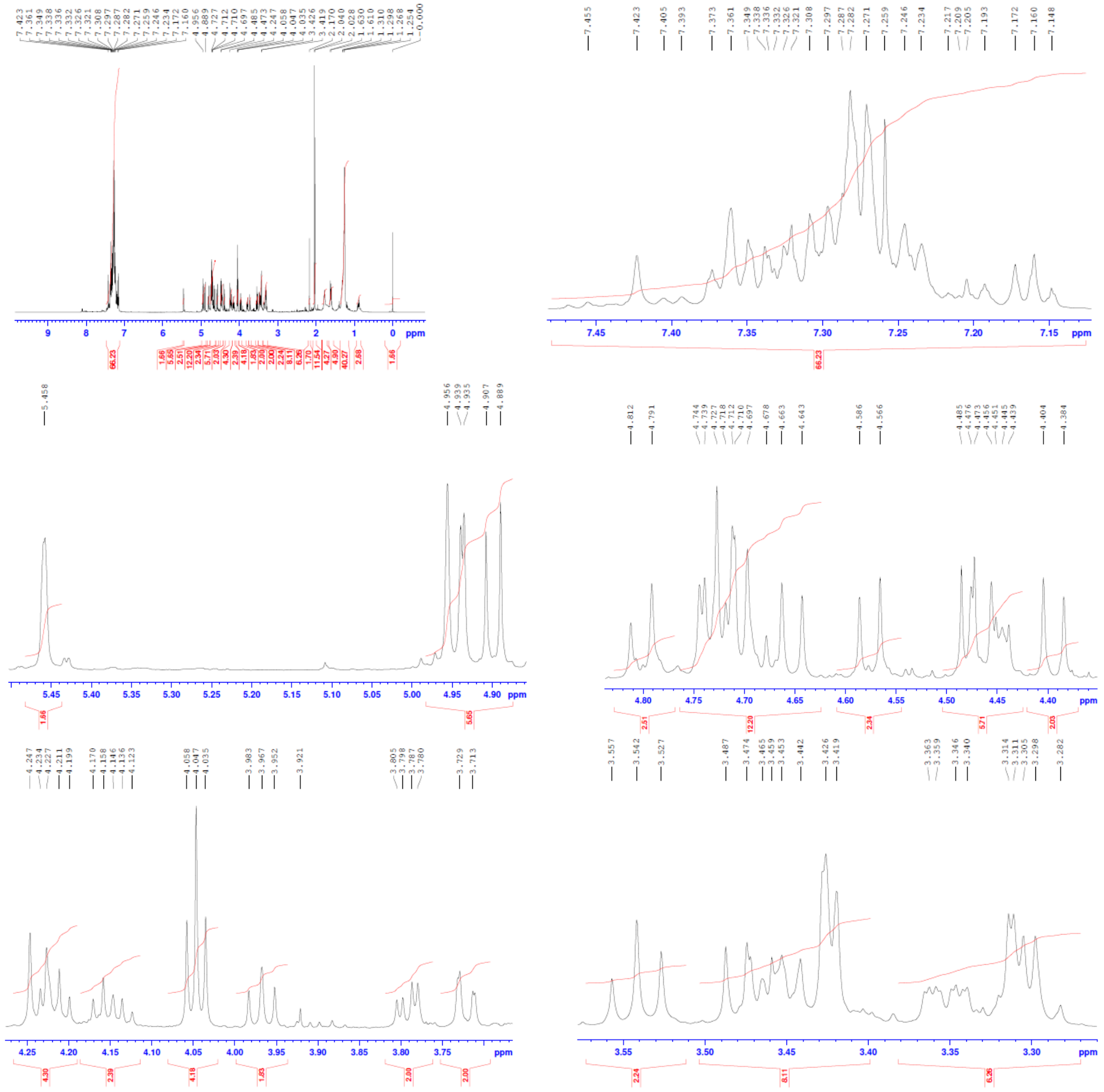

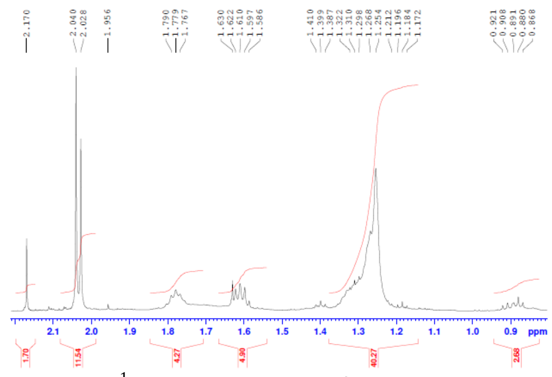

Figs. S47. ^{1}H NMR spectrum of compound **39**.

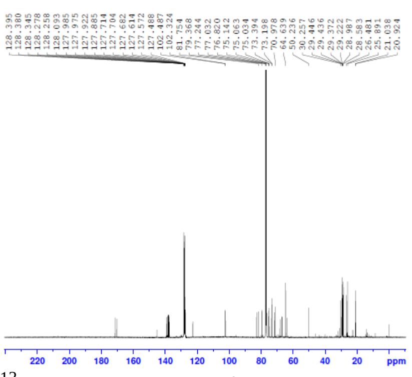

Fig. S48. ^{13}C NMR spectrum of compound **39**.

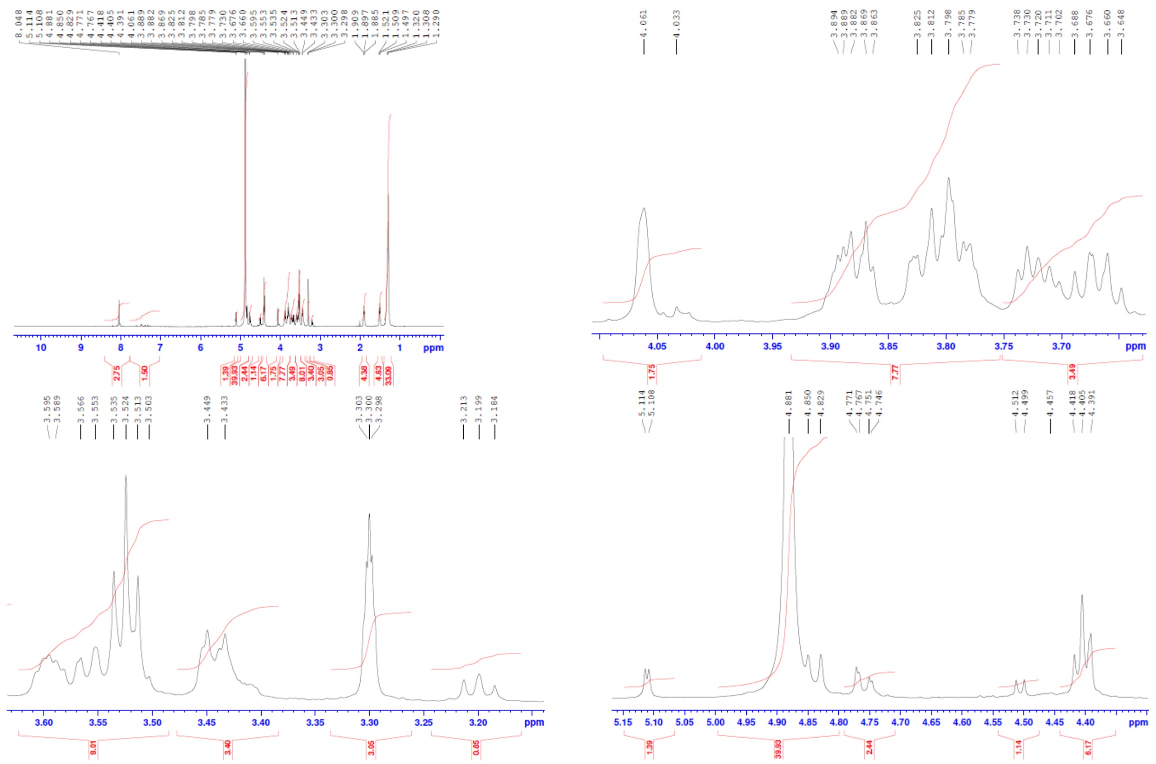

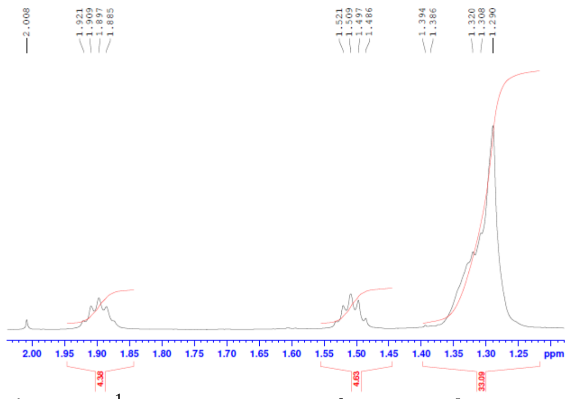

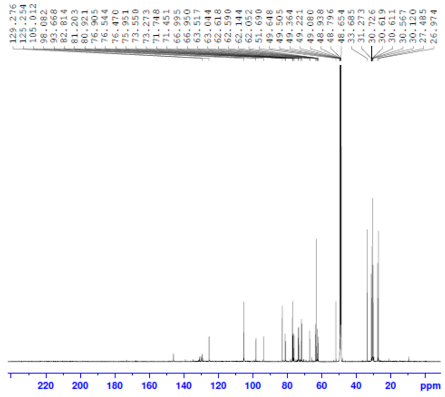

Figs. S49. ^{1}H NMR spectrum of compound **40.** **Fig. S50**. ^{13}C NMR spectrum of compound **40**.

Biology

Antimicrobial screening

The antimicrobial activity of target cholesterol derivatives was determined using a modified Kirby–Bauer disc diffusion method in the Microanalytical Unit, Faculty of Science at Cairo University. Ampicillin and amphotericin B were used as bacterial and fungal positive controls, respectively, while DMSO was used as solvent and negative control as well. Four microbial species were considered, *E. coli* (G^- bacteria), *S. aureus* (G^+ bacteria), *A. flavus* (filamentous fungi) and *C. albicans* (yeast).

Briefly, 100 μL of the test organism were grown in 10 mL of fresh media until they reached a count of 108 cells/mL for bacteria and 105 cells/mL for fungi. 100 μL of the microbial suspension was spread onto Müller–Hinton agar plates. Paper discs (Schleicher & Schüll, Spain) with a diameter of 8.0 mm were impregnated 10 μL of the test compound (4.0 mM) and controls were treated similarly. Plates were incubated for 48 h at 35–37 °C for bacterial strains, 25 °C for *A. flavus* and 30 °C for *C. albicans*. Inhibition zone diameters were measured with slipping calipers. Measurements were taken in triplicates.

Cytotoxicity screening

In vitro anticancer-drug discovery screening of newly synthesized triazoles was performed using the colorimetric cytotoxicity assay for anticancer-drug screening using prostate cancer cell line PC3 at the National Cancer Institute, Cairo University. Cells were plated in 96-multiwell plates (10^4 cells per well) for 24 h to allow attachment of cells to the walls. Monolayer cells were incubated with test compounds individually at concentrations of 5.0, 10.0, 20.0, 30.0, 40.0 and 50.0 μM at 37 °C under 5% CO_2 atmosphere. After 48 h, cells were fixed, washed then stained with sulforhodamine-B stain. Excess stain was washed with acetic acid and attached stain was recovered with *tris*-EDTA buffer. Color intensity was measured in an ELISA reader. The relation between surviving fraction and drug concentration was plotted to get the survival curve for each compound and measurements were taken in triplicates.

Statistical analysis

All results were subjected to one-way ANOVA and the means were compared according to the Student–Newman–Keuls (SNK) multiple range test ($p \leq 0.05$).

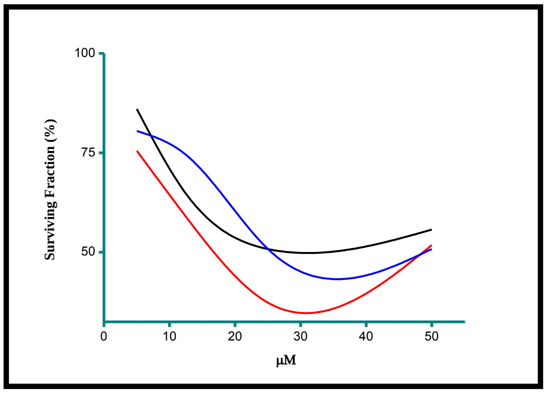

Fig. S51. Kill curve of compd. **6c** against PC3 cell line.

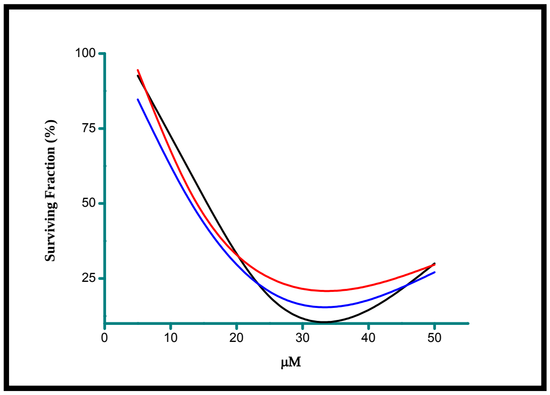

Fig. S52. Kill curve of compd. **11a** against PC3 cell line.

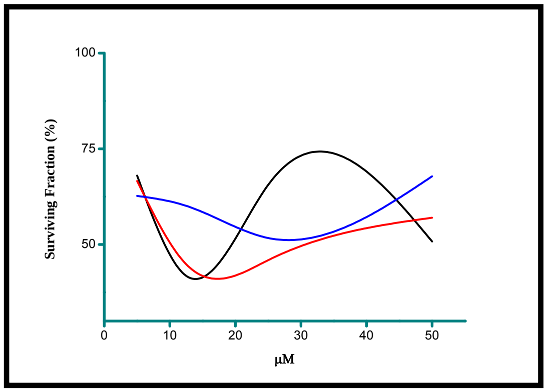

Fig. S53. Kill curve of compd. **12** against PC3 cell line.

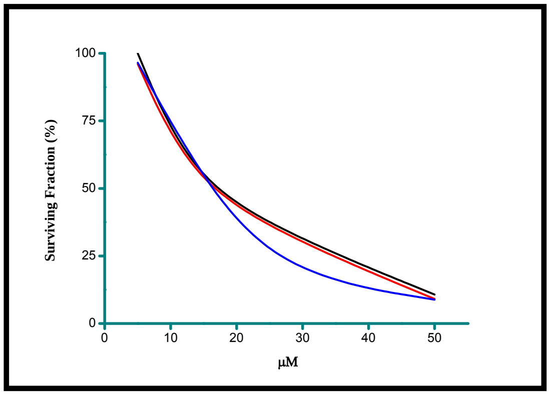

Fig. S54. Kill curve of compd. **13** against PC3 cell line.

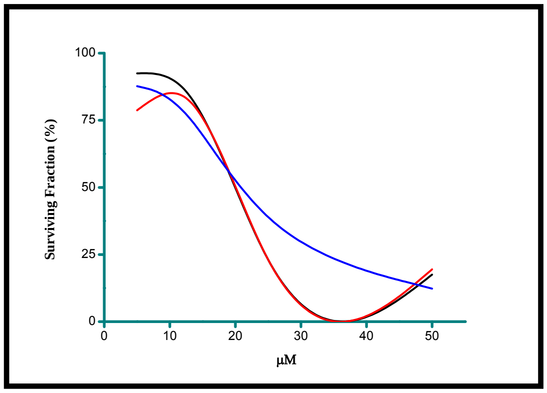

Fig. S55. Kill curve of compd. **16** against PC3 cell line.

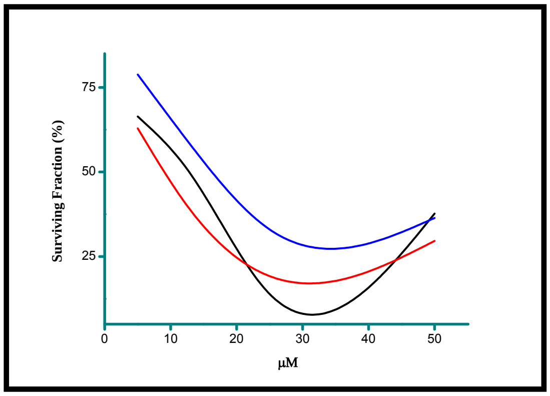

Fig. S56. Kill curve of compd. **17** against PC3 cell line.

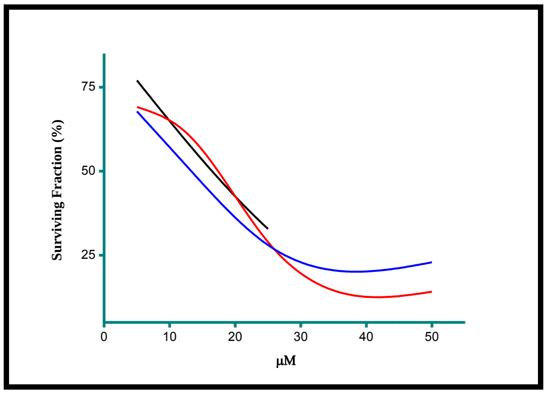

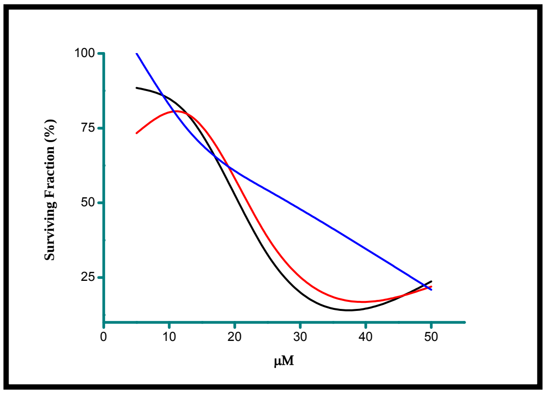

Fig. S57. Kill curve of compd. **24** against PC3 cell line.

Fig. S58. Kill curve of compd. **27** against PC3 cell line.

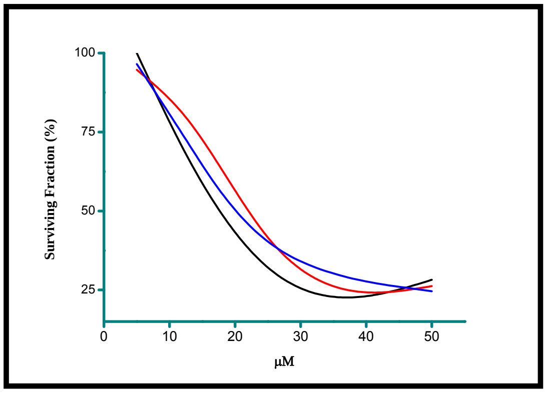

Fig. S59. Kill curve of compd. **40** against PC3 cell line.
